# Structural and mechanism-based engineering of sulfotransferase CHST15 for the efficient synthesis of chondroitin sulfate E

**DOI:** 10.1128/aem.01573-24

**Published:** 2024-12-04

**Authors:** Zhonghua Wang, Wei Song, Wanqing Wei, Hejia Qi, Weiwei Meng, Jia Liu, Xiaomin Li, Cong Gao, Liming Liu, Guipeng Hu, Yiwen Zhou, Jing Wu

**Affiliations:** 1 School of Life Sciences and Health Engineering, Jiangnan University 66374 , Wuxi, China; 2 School of Biotechnology, Jiangnan University School of Biotechnology 546332 , Wuxi, China; 3 Key Laboratory of Industrial Biotechnology, Ministry of Education, Jiangnan University 546774 , Wuxi, China; Kyoto University, Kyoto, Japan

**Keywords:** chondroitin sulfate A, chondroitin sulfate E, whole‐cell catalys, enzyme engineering

## Abstract

Natural chondroitin sulfate (CS), extracted from animal cartilage, is widely used in the pharmaceuticals and foods. However, contamination with animal-derived heteropolysaccharides presents significant challenges, including potential immune responses. To address this, we developed a green and efficient method for synthesizing chondroitin sulfate E (CSE) via enzymatic synthesis, identifying *Ec*CHST15, a sulfotransferase that catalyzes the conversion of chondroitin sulfate A (CSA) to CSE. We investigated the novel catalytic mechanism of CHST15 through quantum mechanical (QM) calculations and experimental validation, confirming its alignment with the SN2 reaction mechanism. Subsequently, we enhanced the catalytic efficiency of CHST15 using protein engineering, improving the catalytic efficiency from 18.1% in the wild type (WT) to 62.5% in the M7 mutant—a 3.5-fold increase. Finally, we constructed a six-enzyme cascade whole-cell catalyst, achieving a 72.2% conversion of 15 g/L CSA to produce CSE within 24 h. These findings offer a promising strategy for the industrial production of CSE.

**IMPORTANCE:**

Current methods for obtaining chondroitin sulfate (CS) primarily rely on tissue extraction and chemical synthesis. However, these approaches are hindered by contamination risks from animal-derived heteropolysaccharides and the technical challenges inherent in complex chemical synthesis, limiting the scalability of industrial CS production. To address this, we developed a green and efficient enzymatic biosynthesis method for chondroitin sulfate E (CSE). By identifying and engineering the sulfotransferase CHST15 from *Erpetoichthys calabaricus*, we created a mutant (*Ec*CHST15^M7^) with a 3.5-fold increase in catalytic efficiency toward chondroitin sulfate A (CSA) compared to the wild-type enzyme. Additionally, we constructed a six-enzyme cascade whole-cell biocatalyst, achieving a 72.2% conversion rate from CSA to CSE. This study opens new avenues for the industrial-scale production of CSE through sustainable enzymatic processes.

## INTRODUCTION

Chondroitin sulfate (CS), a member of the glycosaminoglycan family, consists of glucuronic acid (GlcUA) and N-acetylgalactosamine (GalNAc), linked alternately by β−1,3 and β−1,4 glycosidic linkages. Sulfotransferases commonly sulfate the C-2 position of GlcUA and the C-4 and C-6 positions of GalNAc to varying degrees, resulting in diverse CS structures, including CSA [GlcA-GalNAc (4S)], CSC [GlcA-GalNAc (6S)]], CSD [GlcA (2S)-GalNAc (6S)], and CSE [GlcA-GalNAc (4,6S)] (1). CS exhibits diverse physiological functions, with its sulfation pattern serving as a crucial determinant of these functions. For instance, CSA and CSC serve as viable alternatives to traditional non-steroidal drugs for osteoarthritis treatment (2). Furthermore, CSD and CSE have been documented to facilitate neurite outgrowth in primary neurons, contributing to brain development (3). Beyond medical applications, CS also functions as a food additive, offering emulsification and antioxidation properties, or is incorporated into dietary supplements for pain relief (4). The United States Food and Drug Administration and the European Union have categorized CS as a novel food resource. Presently, commercial CS is predominantly obtained through chemical extraction from animal cartilage tissues, sourced from various animals such as pigs, cattle, and chickens. However, the heterogeneity of raw materials, coupled with the potential immune response triggered by contamination with heteropolysaccharides from animals and the associated risk of interspecific diseases, pose substantial challenges to the accessibility of commercial CS (5, 6). Therefore, there exists a compelling imperative to advance the biosynthesis of CS (7, 8).

In recent years, advancements in enzyme engineering and synthetic biology have systematically addressed the essential prerequisites for CS biosynthesis. The biological synthesis of CS encompasses three primary challenges: the synthesis of precursor substances, the provision of sulfate donor PAPS, and the regulation of sulfate transferase expression and activity. In addressing the issue of precursor synthesis, various recombinant strains, such as *Escherichia coli* (9) and *Bacillus subtilis* (10), have been employed as industrial strains for chondroitin precursor production. Remarkably, research on chondroitin synthesis by microorganisms has reached a relatively advanced stage, enabling large-scale production at the gram level in fermenters. For instance, Zhao et al. employed *E. coli* as the host cells and conducted fed-batch fermentation in a 5-L bioreactor, achieving a chondroitin production of 9.2 g L^−1^ (11). In addressing the availability of sulfate donor PAPS, the enzymes ATP sulfonylase and APS kinase are pivotal in converting ATP into PAPS (12). Aryl sulfotransferase (ASTIV) complements this process by regenerating PAPS from the by-product PAP, thereby effectively recycling PAPS (9, 13). Furthermore, addressing the challenges posed by eukaryotic sulfotransferases, which require post-translational modification leading to limited solubility and reduced activity in heterologous expression, is of utmost importance. Protein engineering is necessary to fundamentally alter the expression and catalytic properties of sulfotransferases. Badri and colleagues utilized the PROSS server to predict CHST11 mutants, with the aim of enhancing the enzyme’s stability and solubility. The predicted mutants (K117R/H127E/S238Y/A245G) resulted in a threefold increase in the catalytic efficiency of CHST11 (14). This highlights the potential of more highly targeted enzyme engineering to further improve *in vivo* sulfation. In addition, the work of Badri et al. was the first to propose and complete the recombinant production of chondroitin, providing a blueprint for the subsequent recombinant production of CS. CS is a macromolecular polysaccharide with a complex molecular structure, which cannot cross the cell membrane and enter the cell directly. Therefore, we hypothesized that sulfotransferases with excellent catalytic properties could be obtained through further protein engineering modification by rational design. And the sulfotransferase was displayed on the cell surface using cell surface display technology, thus realizing the whole-cell biosynthesis of CSE.

This study primarily focused on the screening and engineering of sulfotransferase CHST15, which is responsible for catalyzing the synthesis of CSE from CSA. Initially, through a database mining, we identified an efficient sulfotransferase CHST15 in catalyzing CSA to CSE. Following this, advanced techniques such as Alphafold and molecular dynamics (MD) simulation were employed to determine the structure of CHST15 and the enzyme-ligand complex. Utilizing these structural insights, strategic modifications were made to the cofactor binding cavity to alleviate steric hindrance, thereby enhancing catalytic efficiency. In the next phase, the catalytic mechanism of CHST15 was elucidated using quantum mechanics/molecular mechanics (QM/MM). Building on this understanding, further modifications were introduced to the substrate binding cavity to enhance catalytic efficiency by reducing the energy barrier of the transition state. Finally, a dual-cycle module involving ATP and PAPS was designed to ensure an ample supply of sulfate donors, resulting in a cascade whole-cell catalyst (denoted as P1) that includes six enzymes. This comprehensive approach, from the screening and engineering of CHST15 to the construction and optimization of the whole-cell catalyst, provides a systematic methodology for enhancing CSE synthesis.

## RESULTS

### Screening sulfotransferase for chondroitin sulfate E synthesis

**Fig 1:**
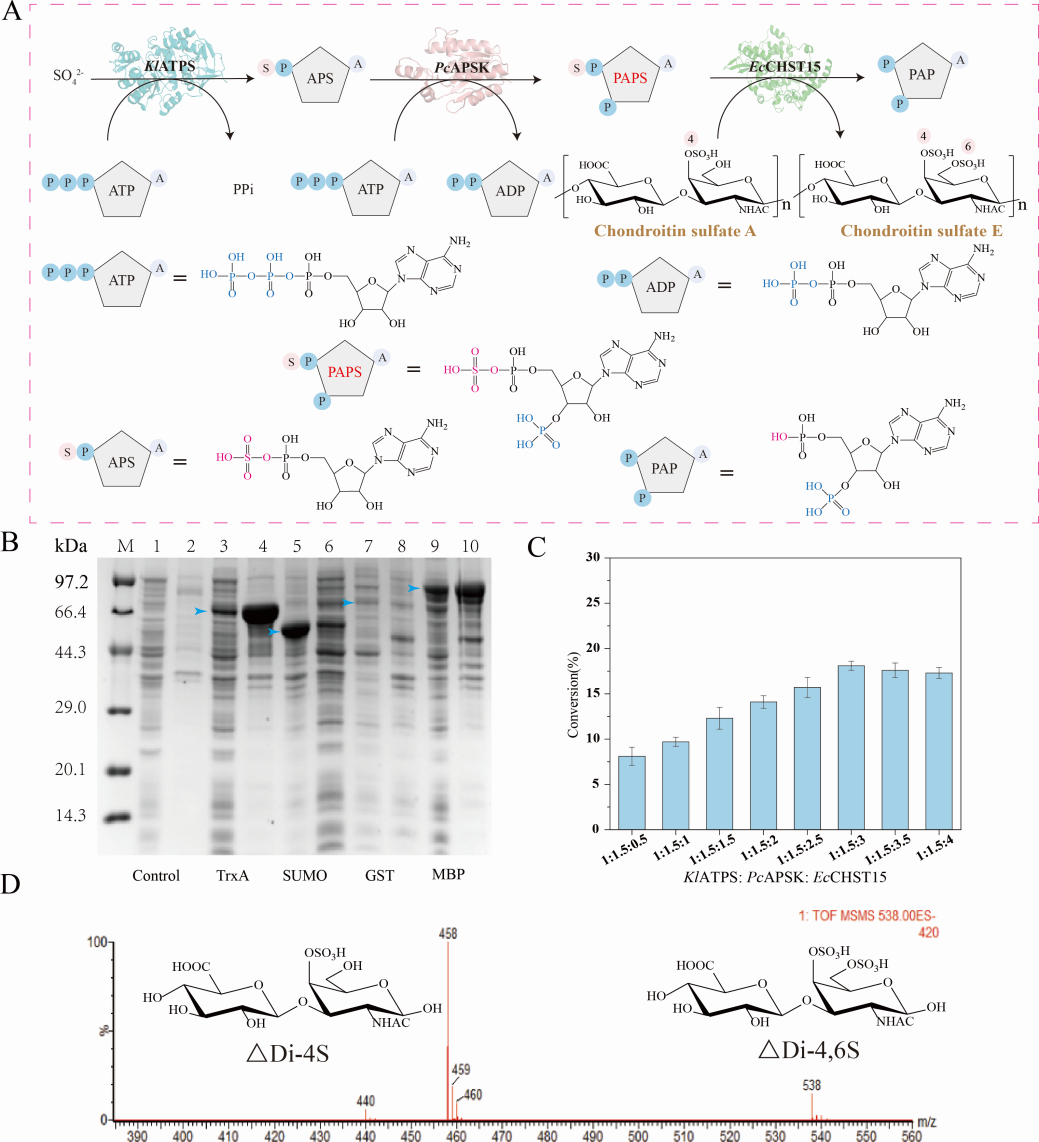
Construction of *in vitro* cascade path. (**A**) Cascade pathway of CSE synthesis *in vitro*. (**B**) SDS-PAGE of solubilization label co-expressed with *Ec*CHST15.M, marker. (1, 2) Supernatants and precipitates of BL21(DE3) cell lysates expressing *Ec*CHST15; (3, 4) supernatants and precipitates of BL21(DE3) cell lysates expressing TrxA-*Ec*CHST15; (5, 6) supernatants and precipitates of BL21(DE3) cell lysates expressing SUMO-*Ec*CHST15; (7, 8) supernatants and precipitates of BL21(DE3) cell lysates expressing SUMO-*Ec*CHST15; (9, 10) supernatants and precipitates of BL21(DE3) cell lysates expressing MBP-*Ec*CHST15. (**C**) Enzyme activity ratio optimization of *Kl*ATPS, *Pc*APSK and *Ec*CHT15. The data represent mean ± SD, as determined from three independent experiments. (**D**) HPLC data monitored at 232 nm for mass spectrometric characterization of CSA disaccharide units (ΔDi-4S) and CSE disaccharide units (ΔDi-4,6S).

To synthesize chondroitin sulfate E (CSE), a gene fragment of the sulfotransferases family, featuring a single sulfonic acid group at chondroitin sulfate A (CSA) GalNAc C6 hydroxyl, served as a template for a BLAST search in NCBI to identify genes with 30–70% similarity. Nine different sources of sulfotransferases (CHST15) were screened from various organisms (Fig. S1), including animals (*Hs*CHST15: *Homo sapiens*, *Ef*CHST15: *Eptesicus fuscus*, *Mm*CHST15: *Mus musculus*, *Ec*CHST15: *Erpetoichthys calabaricus, Lc*CHST15: *Larimichthys crocea*), algae (*Ts*CHST15: *Tetraselmis sp*. GSL018), plants (*Ph*CHST15: *Podila humilis*, *Pv*CHST15: *Podila verticillata*), and bacteria (*At*CHST15: *Acidithiobacillus thiooxidans* ATCC 19377). Out of these, only four CHST15 proteins (*Ec*CHST15, *Lc*CHST15, *Ts*CHST15 and *At*CHST15) were successfully expressed, with *Ec*CHST15 showing the highest sulfation activity at 44.6 U/mL (Fig. S2). Due to the low soluble expression level of CHST15, the strategy of co-expression solubilizing tags was conducted to increase the soluble expression level of *Ec*CHST15. Specifically, maltose binding protein (MBP), thioredoxins (Trx), small ubiquitin-related modifier (SUMO), and glutathione *S*-transferase (GST) were used as solution-promoting tags (Fig. 1B). The co-expression of MBP tag increased the enzyme activity of *Ec*CHST15 by 2.8-fold, reaching 125.1 U/mL (Fig. S3). Optimizing protein expression conditions is a common method to improve enzyme solubility (14). optimized the expression conditions for sulfotransferase C4ST, which synthesizes CSA, achieving the highest protein expression and sulfation efficiency under induction at OD_600_ and 1 mM IPTG concentration at 16°C for 12 h. Therefore, We then optimized the protein expression conditions, achieving the highest expression of CHST15 protein and activity under induction at 16°C, OD_600_, and 0.8 mM IPTG concentration, resulting in an enzyme activity of 134.8 U/mL (Fig. S4).

To identify the sulfation product of *Ec*CHST15, an *in vitro* purified enzyme conversion system was established. Based on our previous study on PAPS production via a two-step enzymatic cascade with sulfate and ATP as substrates, we proposed employing PAPS as a sulfate donor and CSA as a sulfate acceptor to synthesis CSE catalyzed by *Ec*CHST15 (Fig. 1A). ATP sulfatase from *Kluyveromyces lactis* (*Kl*ATPS) and adenosine-5′-phosphosulfate kinase from *Penicillium chrysogenum* (*Pc*APSK) were chosen through literature mining to construct the PAPS synthesis pathway, utilizing one molecule of SO_4_^2−^ and two molecules of ATP as substrates. In a 2-mL pure enzyme reaction system, pure enzymes (*Kl*ATPS, *Pc*APSK, and *Ec*CHST15, molar ratio 1:1:1) were incubated with 60 mM ATP, 100 mM Na_2_SO_4_, and 5 g/L CSA to generate CSE. The produced CSE was purified and completely digested with chondroitinase ABC I. The resulting disaccharide was characterized by high-performance liquid chromatography-mass spectrometry (HPLC-MS) and tandem mass spectrometry (MS/MS) (Fig. 1D). The obtained CS disaccharide is consistent with the standard. The molecular weight of the unsaturated disaccharide ion produced in negative ion mode ionization is 538.01 *m/z*. The conversion was 12.3% by external standard method. The optimal enzyme molar ratio was determined by fixing the amount of *Kl*ATPS, the highest conversion reached 18.1% when *Kl*ATPS:*Pc*APSK:*Ec*CHST15 was 1:1.5:3 (Fig. 1C). Since more *Ec*CHST15 enzyme is required in the *in vitro* cascade, *Ec*CHST15 was the rate-limiting enzyme in the pathway.

### Structure analysis of *Ec*CHST15

To comprehend the molecular mechanism underlying the *Ec*CHST15 catalyzed reaction, attempts were made to crystallize *Ec*CHST15, but unfortunately, the crystal structure could not be obtained. Consequently, AlphaFold2 was employed to predict its 3D structure, and the residue confidence score (pLDDT) results indicated a high level of reliability in the protein structure (Fig. S5A). *Ec*CHST15 is anticipated to be a Golgi membrane protease with a predicted structure comprising 15 α-helices and 13 β-folded sheets across three domains: topological (1–80 AA), transmembrane (81–101 AA), and catalytic (102–561 AA) domains (Fig. S5B). Among these, the topology domain forms the loop ring structure located in the cytosol at the N end. The transmembrane domain, functioning as a signal anchor for type II membrane proteins, is an α-helix composed of hydrophobic residues embedded in the Golgi membrane. The C-terminal catalytic domain resides in the Golgi compartment and is connected to the transmembrane domain by a flexible loop (stem region), potentially keeping the catalytic domain away from the lipid bilayer and providing improved access to the substrate. Previous reports have demonstrated that the topological and transmembrane domains are not involved in glycosaminoglycan formation, and the catalytic domain alone can sulfate glycosaminoglycans in soluble form within the Golgi. Therefore, to mitigate potential interference with proper folding and activity, we truncated the topological and transmembrane domains (1–120 AA) of *Ec*CHST15 during heterologous expression in *E. coli*.

**Fig 2:**
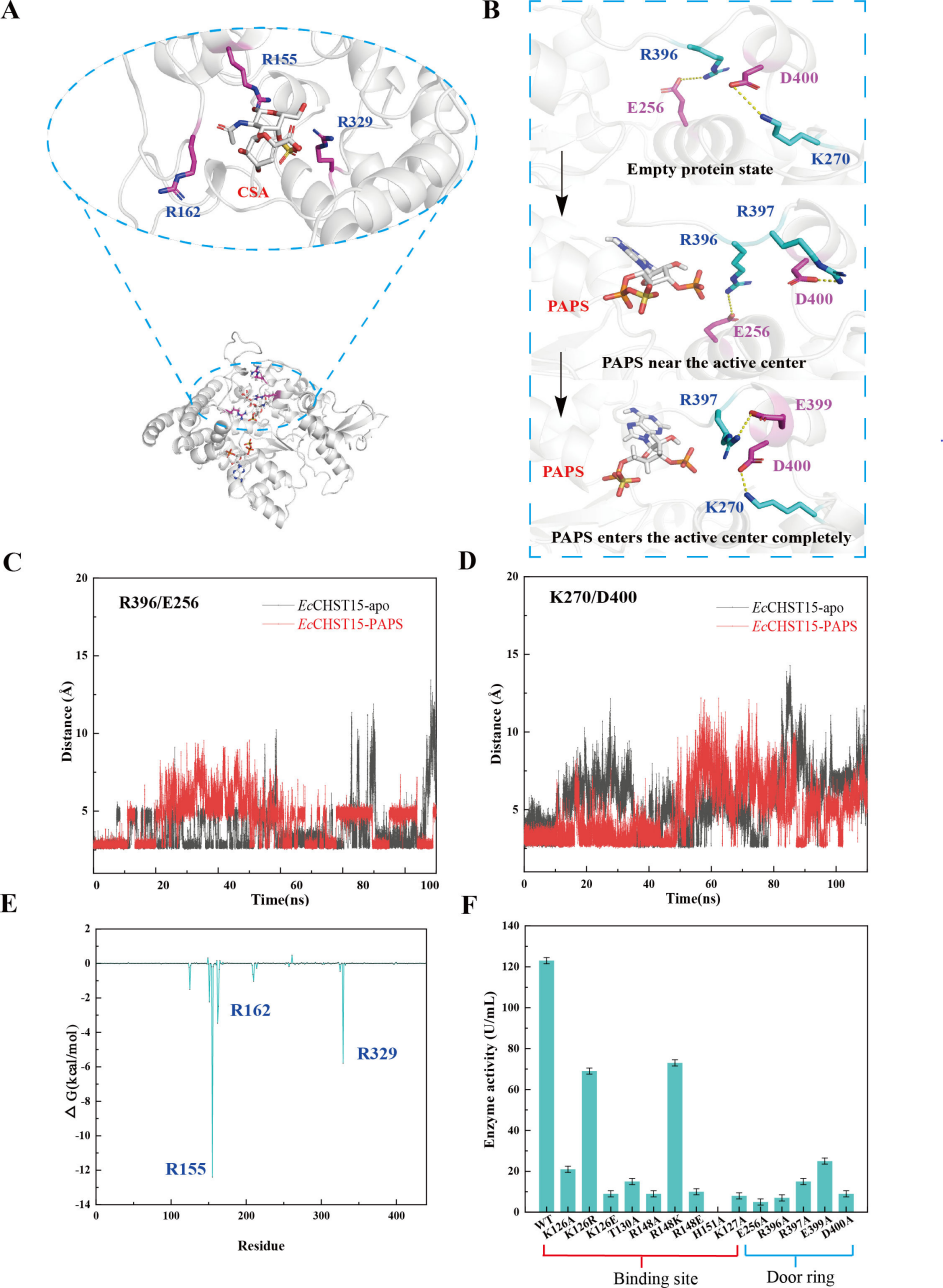
Structure analysis of *Ec*CHST15. (**A**) CSA binding mechanism (positively charged residues in blue). (**B**) PAPS binding mechanism (salt bridge forces are shown in yellow dashed lines, negatively charged residues in purple and positively charged residues in blue). (**C**) Distance variation between R396/E256 pairs. (**D**) Distance variation between K270/D400 pairs. (**E**) MM-GBSA calculates the binding free energy per residue of *Ec*CHST15-CSA. (**F**) Validation of key residue mutation. The data represent mean ± SD, as determined from three independent experiments.

To investigate the binding mechanism of PAPS and CSA, we conducted a comparison of the active centers of similar sulfotransferases in the PDB database. Such as 3-O sulphotransferase (ID: 1VKJ), human estrogen sulphotransferase (ID: 1HY3). We identified a typical sulfotransferase-substrate cofactor ternary complex structure, where PAPS is located in binding sites characterized by chain-loop-helix and chain-translocation-helix structures, including 5′-phosphate sulfate binding loop (PSB ring) and 3′-phosphate binding loop (PB ring). The substrate fits into the open cavity containing a positive charge, with its hydrophilic surface perpendicular to the PAPS 5′-phosphate. Subsequently, PAPS and CSA disaccharide units were docked into the active site of *Ec*CHST15 using Autdock software (Fig. 2A), and MD simulations of the *Ec*CHST15-PAPS-CSA triad were performed for 100 ns. During the simulation, the surrounding environment of the ligand-binding pocket was analyzed, revealing numerous highly conserved residue pairs and opposing charges in cofactor binding cavity. Residues R396 and E256, along with the interplay between K270 and D400, were identified as crucial in modulating the opening and closing of protein conformations, controlling entry and release of PAP(S).

The binding mechanism of PAPS was visualized in three states: First, in the absence of PAPS binding, a salt bridge was formed between R396 and E256, as well as between K270 and D400, resulting in the closure of the door ring. Second, as PAPS approached the active center, the noncovalent bond between K270 and D400 broke, and D400 formed a salt bridge with R397 on the same chain, leading to a semi-open state of the gate loop. Finally, when PAPS fully entered the active center, the noncovalent bond between R396 and E256, R397 and D400 broke, and D400 re-established a salt bridge with K270, reinstating the “closed-loop” effect (Fig. 2B).

To validate the PAPS binding, key residues were mutated through alanine scanning. As anticipated, mutants K127A, E256A, R396A, R397A, E399A, and D400A exhibited significantly reduced activity 76–92% (Fig. 2F). To observe alterations in the conformation of the cofactor cavity, we monitored movements between R396/E256 and K270/D400 salt bridges. The change in distance between the Cα atoms of the salt bridge backbone was used as the motion trajectory. In *Ec*CHST15-*apo,* the R396/E256 pair was relatively stable with an average distance of about 3 Å during MD simulation, while the R396/E256 pair was highly variable with an average distance of about 5 Å. However, in *Ec*CHST15-PAPS module, the average distance between R396 and E256 increased to about 7 Å within 20–50 ns, at which point the salt bridge between R396/E256 breaks. Subsequently, the average distance between R396 and E256 reduced to about 3 Å within 50–100 ns, and the salt bridge was re-formed (Fig. 2C). The K270/D400 pair was relatively stable in the first 50 ns, and the average distance increased to about 8 Å within 50–80 ns, and the salt bridge between K270/D400 broke. Subsequently, the average distance between K270 and D400 reduced to about 5 Å within 50–100 ns, and the salt bridge was re-formed (Fig. 2D). This supports the hypothesis that binding and dissociation between the above two salt bridges can regulate the opening and closing of protein conformations. Additionally, a cluster of three arginine residues, R155, R162, and R329, formed a positively charged fork, anchoring CSA to the active center (Fig. 2A). Notably, these residues were found to be highly conserved in the eukaryotic CHST15 enzyme (Fig. S6), suggesting their pivotal role in positioning the ligand within the active site. Furthermore, to quantify the contribution of key residues to the overall binding, MM-GBSA per residue energy decomposition analysis was conducted for *Ec*CHST15-PAPS-CSA (Fig. 2E). The total combined free energy △G for *Ec*CHST15-CSA was −30.6 kcal/mol. The contributions of R155, R162, and R329 to substrate binding were −12.4 ± 0.8, −3.5 ± 0.6, and −5.8 ± 0.6 kcal/mol, respectively, collectively contributing to a total binding free energy of −21.9 ± 0.9 kcal/mol for *Ec*CHST15-CSA. These findings suggest that these three residues, especially R155, play a fundamental role in substrate binding.

### Structural-based engineering of *Ec*CHST15 for improving the binding efficiency of PAPS

**Fig 3:**
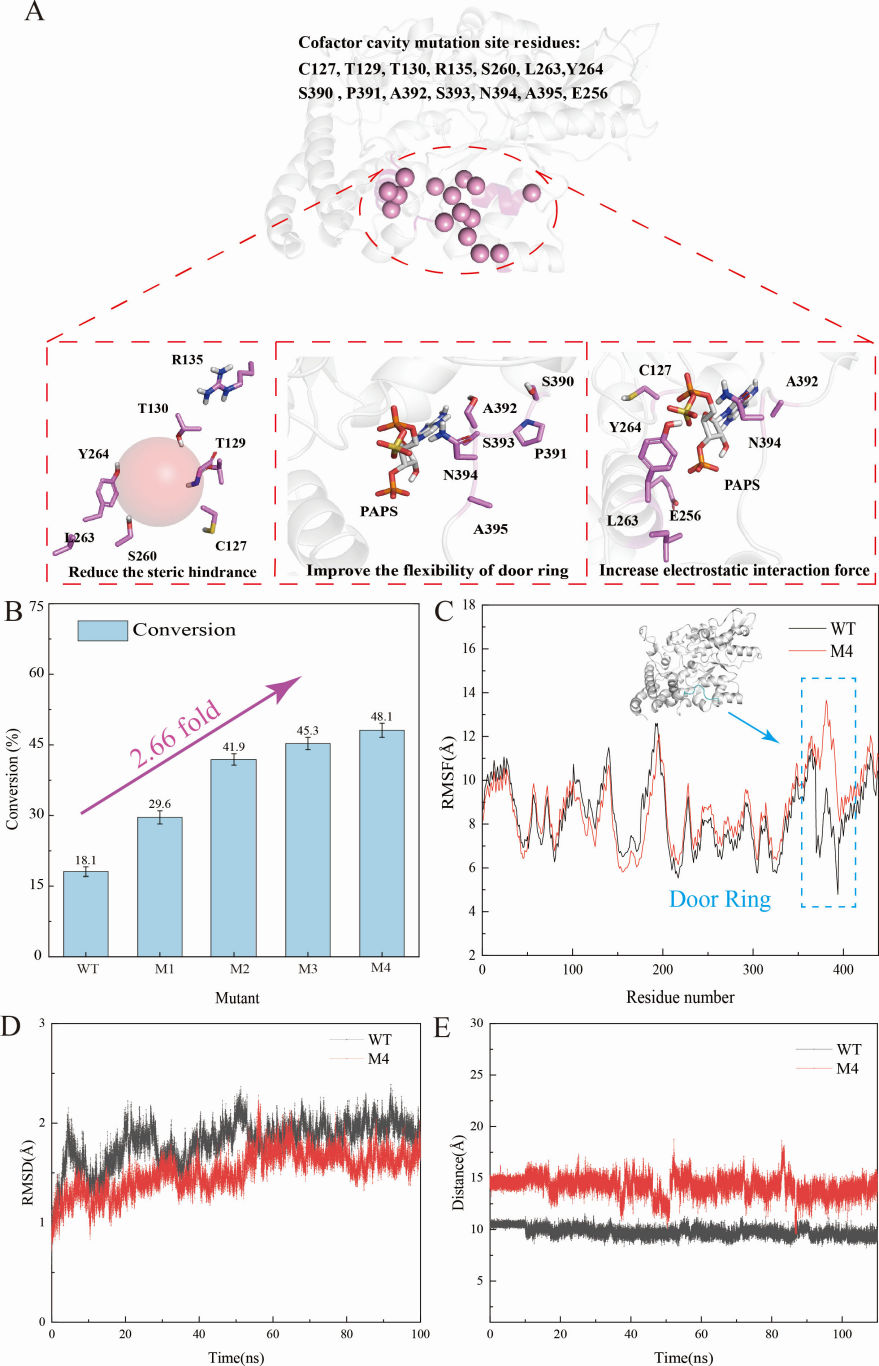
*Ec*CHST15 protein engineering site selection based on structural engineering guidance. (**A**) Cofactor cavity modification site, including reducing steric hindrance strategy selection point, improving the flexibility of the door ring strategy selection point, and enhancing the electrostatic interaction force strategy selection point. (**B**) The conversions of *Ec*CHST15 mutants (round 1 to round 4). (**C**) MD simulations calculate RMSD values for WT and M4. (**D**) MD simulates the RMSF values for WT and M4. (**E**) Changes in the distance between K126 and N394 αC during MD simulations.

To reveal the reason for the low catalytic efficiency at the structural level, the structure of *Ec*CHST15-PAPS-CSA ternary complex was analyzed. The result showed that the volume of PAPS was 1,302 Å^3^, while the volume of cofactor binding cavity was only 1,205 Å^3^, slightly smaller than the volume of PAPS (Fig. S9). This discrepancy may result in a reduced binding efficiency of PAPS, consequently affecting the sulfation reaction. To improve the binding efficiency of PAPS, three mutation strategies are proposed for the redesign of the cofactor binding pocket, including reducing the steric hindrance, improving the flexibility of the door ring, and enhancing the affinity for the cofactor (Fig. 3A). To reduce the steric hindrance in binding pocket, site-directed mutagenesis was employed, where large amino acid residues (C127, T129, T130, R135, S260, L263, and Y264) within 5 Å range of cofactor were mutated to small amino acid residues. To improve the flexibility of the door ring, the rigid residues (S390, P391, A392, S393, N394, and A395) in the door ring were mutated to flexible residues (Ser, Gly, and Ala) to improve its opening and closing efficiency. Regarding the affinity for the cofactor, given the negatively charged nature of PAPS, introducing positively charged residues in the binding cavity facilitates ligand anchoring through electrostatic interaction forces. Therefore, non-conserved residues (C127, E256, L263, Y264, A392, and N394) within 5 Å range of the cofactor were mutated to positively charged residues to enhance electrostatic interactions.

Thus, three mutation libraries, comprising 15 mutation sites and yielding 45 mutants, were constructed (Table S4). Screening of beneficial mutants by HPLC assay. Ten beneficial mutants were identified, with M1 (A395G, 29.6% conversion) demonstrating the most significant improvement in sulfation efficiency. Subsequently, 1, 9-dimethyl-methyl blue (DMMB) color development method was used for high-throughput screening. Based on M1, other beneficial mutation sites were iteratively combined, resulting in the most efficient double, triple, and quadruple mutants, which were M2 (A395G/N394S, with 41.9% conversion), M3 (A395G/N394S/Y264S, with 45.3% conversion), M4 (A395G/N394S/Y264S/C127G, with 48.1% conversion). As shown in Fig. 3B and Table S4, the quadruple mutant M4 was selected as a beneficial mutant, achieving the highest conversion of 48.1%. This represented a 30% increase in sulfation efficiency compared to WT.

To gain a deeper understanding of the enhanced sulfation efficiency of M4, we constructed the *Ec*CHST15^M4^-PAPS-CSA model and subjected it to 100 ns MD simulations. CAVER_Web_ was used to calculate the cofactor binding tunnel for both the WT and M4 (Fig. S10). We assessed the bottleneck of the evaluation channel based on the distance between the main chain Cα atom of N394 and K126. In the WT, the distance is constrained to 8.5–0.5 Å, with the bulky N394 occupying significant space, potentially impeding PAPS access. Conversely, the N394 to S394 mutation in M4 expanded the channel bottleneck to 10.0–15.5 Å (Fig. 3E). Furthermore, the introduction of N394S, A395G, Y264S, and C127G in M4 resulted in a 3.9 Å increase in the root mean square fluctuation (RMSF) value at the “door ring” (Fig. 3C). The average RMSD of WT was 1.9 Å, whereas the average RMSD of M4 was 1.6 Å, indicating an average RMSD decrease of 0.3 Å (Fig. 3D). These results suggest that the A395G and N394S mutations enhance the flexibility of the “door ring,” while the N394S, C127G, and Y264S mutations broaden the PAPS binding channel and improve the stability of protein. This collective effect accelerates the overall sulfation efficiency. Subsequently, the kinetic parameters of WT and M4 were determined. In comparison to WT, the *K*_m_ value of M4 decreased from 7.11 to 5.06 mM, *k*_cat_ decreased from 0.068 to 0.053 s^−1^, and *k*_cat_/*K*_m_ increased from 9.56 to 10.47 s^−1^ M^−1^ (Table S5). These results indicated that a strategy to retrofit *Ec*CHST15 based on structural engineering guidance is feasible.

### Mechanism analysis of *Ec*CHST15

**Fig 4:**
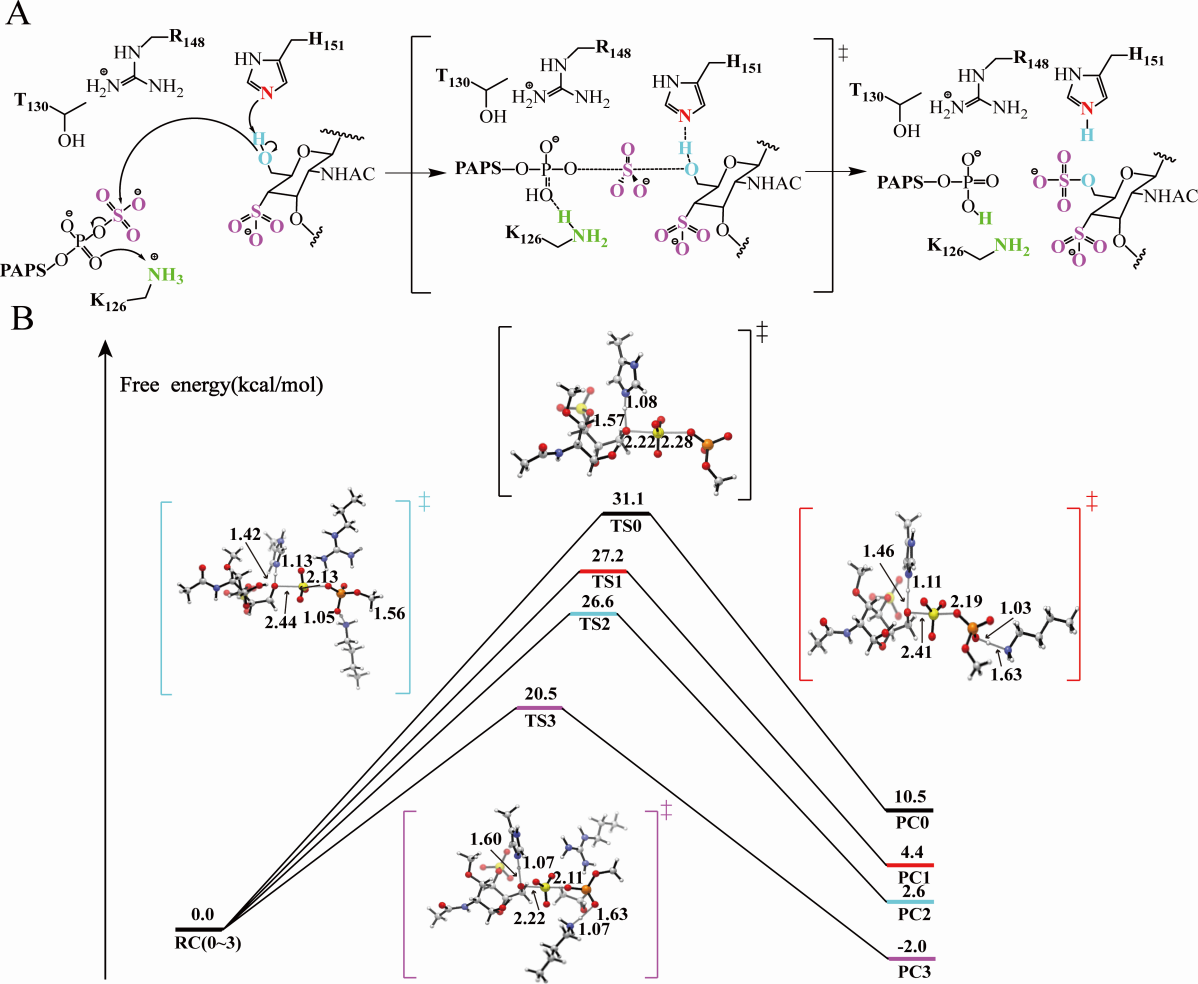
Elucidation of the sulfation mechanism. (**A**) Mechanism of *Ec*CHST15 sulfation reaction. (**B**) Energy barrier diagram of sulfation reaction of *Ec*CHST15. The black border is the TS0] state, the red border is the (TS1) state, the blue border is the [TS2] state, and the purple border is the [TS3] state. (The ground state model is named model 1, model 1 + LYS residue is named model 2, and model 1 + LYS + ARG residues are named model 3. Model 1 + LYS + ARG + THR residues are named model 4.)

To enhance the catalytic performance of *Ec*CHST15, we conducted literature research and conservation analysis of homologous enzymes, identifying the catalytic residues of *Ec*CHST5 (K126, T130, R148, and H151), and proposed its catalytic mechanism (Fig. 4A; Fig. S6). In this mechanism, the oxygen atom in the hydroxyl group of CSA at C6 position attacks the sulfur-oxygen bond of PAPS, with the imidazolyl nitrogen of H151 serving as a generalized base to capture the proton on the hydroxyl group of CSA to enhance its nucleophilicity. Positive charges from K126 and R148 residues stabilize the negative charges accumulated in the transition state by electrostatic interactions, thereby accelerating this nucleophilic substitution reaction. The SN_2_-like reaction mechanism of *Ec*CHST15 was validated through mutagenesis experiments and energy calculations. First, K126, T130, R148, and H151 were mutated to alanine or amino acids with opposite electrical properties, and the changes in the activity of the mutants were observed. As anticipated, K126 and R148 mutation into oppositely charged residues (K126E and R148E) led to almost complete deactivation. K126 and R148 mutations into the amino acid of the same charge only lead to enzyme activity declining slightly. The enzymatic activity of K126R decreased from 125.1 to 70.5 U/mL, and that of R148K decreased from 125.1 to 75.6 U/mL. Alanine scanning of the key catalytic residues showed that the H151A mutant was completely inactive. The enzymatic activities of the K126A, R148A, and T130A mutants decreased from 125.1 to 20.1, 10.7, and 18.6 U/mL, respectively, with almost complete loss of activity (Fig. 2F). Second, a ground-state model was constructed using GaussView, including RC, TS, and PC representing three different states, and the energy of the model was calculate. Key residues K126, T130, R148, and H151 were then added to the ground state model, and the energy changes of the three different states were recalculated to verify the sulfation mechanism from the perspective of thermodynamics and kinetics (Fig. S7). As expected, the formation of PC0 state in the ground state model required 10.5 kcal/mol, the ground state model was named model 1, with △*G* > 0 indicating an endothermic reaction unlikely to occur at room temperature. Added K126, T130, and R148 key residues into model 1 formatted model 4, Δ*G* gradually decreased from 10.5 to −2.0 kcal/mol. This shift resulted in an exothermic reaction (Δ*G* > 0), making it spontaneously achievable at room temperature (Δ*G* < 0). The energy barrier of (TS0) in model 1 was 31.1 kcal/mol, and the addition of key amino acids LYS, ARG, and THR successively lowered the energy barrier. Finally, the energy barrier of (TS3) for model 4 was 20.5 kcal/mol, making the entire sulfation reaction more facile (Fig. 4B; Fig. S8). In summary, the catalytic mechanism of *Ec*CHST15 follows the typical SN_2_ reaction mechanism of sulfotransferases, and K126, T130, R148, and H151 are key residues in the catalytic process.

### Mechanism-based engineering of *Ec*CHST15 for reducing the energy barrier of transition state

We further elucidate the reason for the low catalytic efficiency at the mechanism level. The catalytic process of *Ec*CHST15 was analyzed through QM, revealing the presence of a transition state, denoted as [TS3], characterized by a high energy barrier of 20.5 kcal/mol, significantly impacting sulfation efficiency. Consequently, reducing the energy barrier of this transition state emerges as a potential avenue for improving the enzyme catalytic efficiency. An exploration of the changes in enzyme and substrate during the transition from the reactant complex (RC) to [TS3], identified a modification method to lower the energy barrier of [TS3]. Employing restricted MD simulations (where the distance between O6@RC and S1@RC is less than 2.6 Å) replicated the [TS3] state within the active cavity under reaction conditions. This led to the generation of the theoretical docking model termed *Ec*CHST15-PAPS-CSA-[TS3]. A comparison between the *Ec*CHST15-PAPS-CSA-[TS3] and *Ec*CHST15-PAPS-CSA models revealed a substrate conformational shift of approximately 130° relative to the [TS3] conformation during the RC state transition to [TS3]. Additionally, the cofactor exhibited a shift of about 90° relative to the [TS3] conformation, accompanied by a closure trend in the loop around the ligand (V161-D165, F324-V328, and S390-P398) (Fig. S11). Based on restricted MD simulations, unfavorable intermolecular forces were identified as a key factor contributing to the high-energy barrier of the transition state, and hindering the transition of ligands from ground state to transition state. This phenomenon was particularly pronounced in PAPS state transition, where the hydrogen bond between the hydroxyl group on PAPS 5′-phosphate and N394 and Y264 increased the difficulty of PAPS transitioning from RC state to [TS3] state. Moreover, the side chain of W213 and P125 were observed to exert a significant steric hindrance, potentially destabilizing the transition state and leading to a high-energy barrier.

**Fig 5:**
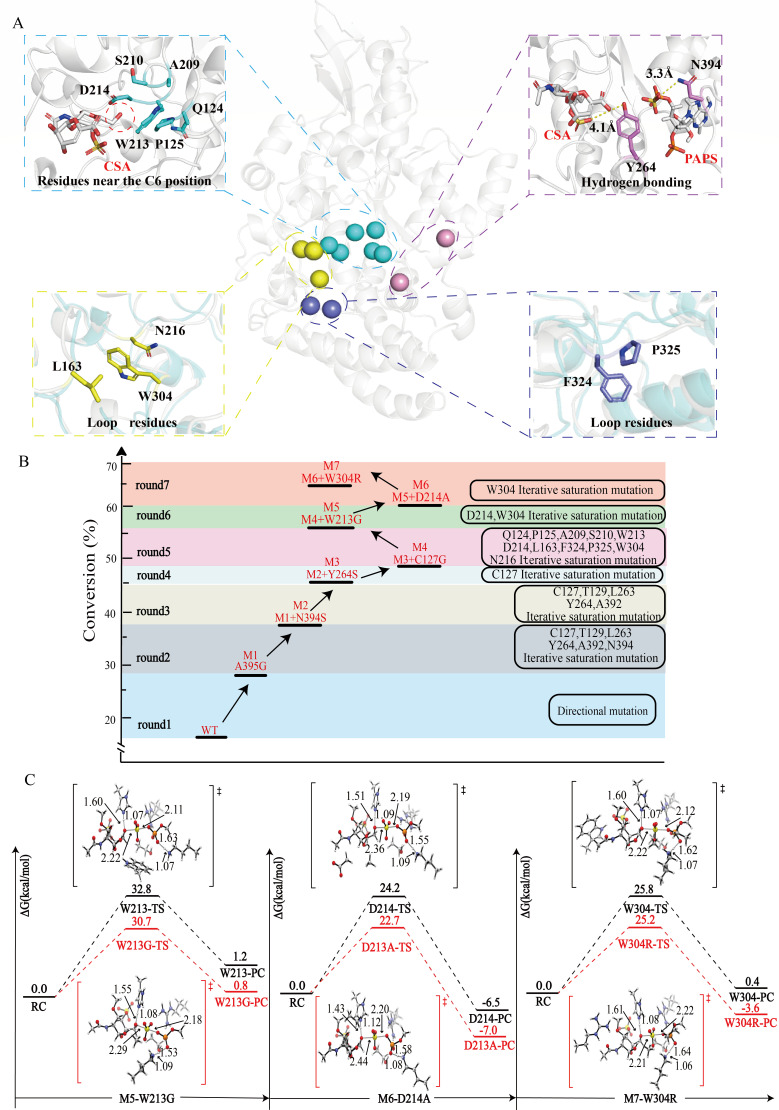
Site selection and mutant analysis of *Ec*CHST15 protein engineering guided by catalytic mechanism. (**A**) Substrate cavity modification site. (**B**) Phylogenetic tree of *Ec*CHST15. (**C**) Combined with QM calculations to resolve mutants, including M5, M6, and M7 mutants.

We undertook a redesign of the substrate binding pocket based on the observed phenomena, adhering to principles that aimed to stabilize the transition state while simultaneously disrupting the ground state. Key residues (C127, Y264, N394, W213, and P125) related to the high-energy barrier step were selected to eliminate the negative effects. Considering the synergistic effects of neighboring residues, additional residues such as Q124, P125, A209, S210, D214, N216, W304, F324, and P325 were also considered (Fig. 5A). Therefore, using the M4 mutant as a template, single saturation mutations were performed on the selected 13 amino acid sites. Although the sulfation efficiency of most mutants decreased, the introduction of mutations such as W213G, W213A, D214N, W304R, W304A, and W304G leads to an increase in sulfation efficiency ranging from 1.3% to 6.5%. To further enhance sulfation efficiency, an iterative combination of three residues (W304, N214, and W304) was performed. A beneficial mutant, M7 (A395G/N394S/Y264S/C127G/W213G/D214A/W304R), was identified, exhibiting a conversion of 62.5%, a 44.4% increase compared to WT (Fig. 5B). Subsequently, the kinetic parameters of M7 were determined. In comparison to M4, the *K*_m_ value of M7 decreased from 5.06 to 4.41 mM, *k*_cat_ decreased from 0.053 to 0.047 s^−1^, and *k*_cat_/*K*_m_ increased from 10.47 to 11.03 s^−1^ M^−1^ (Table S5). These results indicated that a strategy to retrofit *Ec*CHST15 based on catalytic mechanism guidance is feasible.

To further uncover the reasons for the enhanced sulfation efficiency, we constructed *Ec*CHST15^M5^-PAPS-CSA, *Ec*CHST15^M6^-PAPS-CSA, and *Ec*CHST15^M7^-PAPS-CSA models, subjecting them to 100 ns MD simulations. As shown in Fig. S12, the indole portion of the W213 side chain wedges into the deep pocket, potentially causing a clash with CSA, thereby affecting the efficiency of enzyme-substrate binding. Mutating W213 to glycine releases a significant amount of space, making it easier for the enzyme to bind with the substrate. The carboxyl group of D214 forms a hydrogen bond with the hydroxyl group on the CSA side chain, hindering the transition of CSA from the RC state to the [TS3] state. Mutating D214 to alanine disrupts unfavorable hydrogen bonding, reducing the difficulty of CSA transitioning from the RC state to the [TS3] state. Additionally, the W304R mutation increases electrostatic interactions, making it easier to anchor the substrate within the binding cavity. Building upon model 4, successive addition of W213G, D214A, and W304R was performed to calculate the transition state energy barrier and total energy barrier changes (Fig. 5C). When W213 is mutated to alanine, the energy barrier is reduced by 2.1 kcal/mol in the TS state and by 0.4 kcal/mol in the PC state. When D214 is mutated to glycine, the energy barrier is reduced by 1.5 kcal/mol in the TS state and by 0.5 kcal/mol in the PC state. When W304 is mutated to arginine, the energy barrier is reduced by 0.6kcal/mol in the TS state and by 4.0 kcal/mol in the PC state. This significant reduction in the energy barrier is mainly a reduction in the transition state energy barrier, consistent with our original enzyme design principles.

### Constructing whole-cell catalyst for CSE synthesis

**Fig 6:**
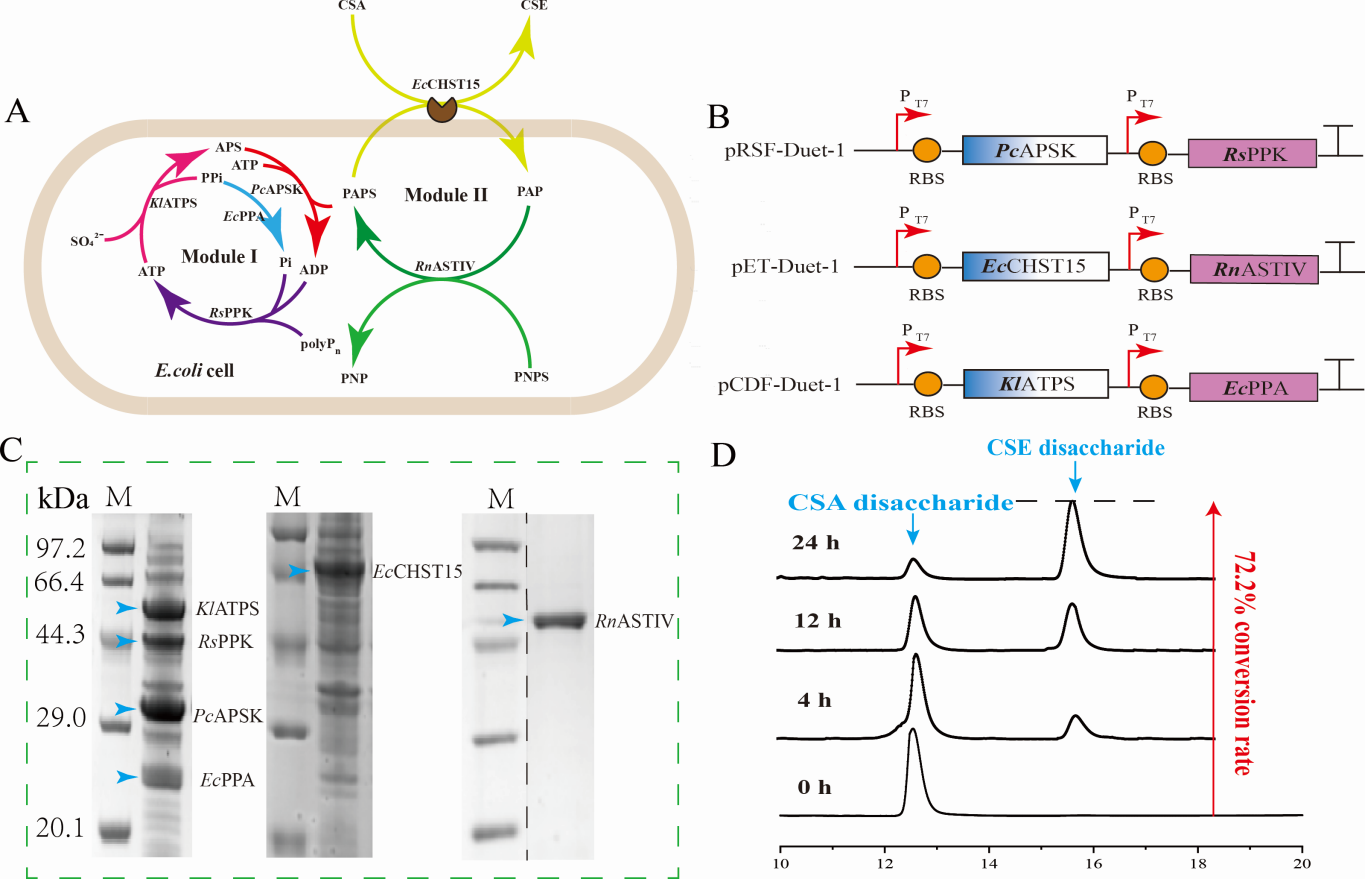
A whole-cell catalyst for CSE synthesis was constructed. (**A**) Schematic representation of the BL21 whole-cell catalytic system. (**B**) Schematic diagram of the engineered strain P1. The components of the chassis cells were assembled to express *Pc*APSK and *Rs*PPK with a high copy number (100) plasmid pRSF-Duet-1 (pRSF-Duet-1-*Pc*APSK-*Rs*PPK). Plasmids pET-Duet-1 with medium copy number (40) were used to express INPN-*Ec*CHST15^M7^ and *Rn*ASTIV(pET-Duet-1-PelB-INPN-*Ec*CHST15^M7^-TrxA-*Rn*ASTIV), and plasmids pCDF-Duet-1 with low copy number (15) were used to express *Kl*ATPS and *Ec*PPA(pCDF-Duet-1-*Kl*ATPS-*Ec*PPA), which were transferred into *E.coil* Rosetta (DE3). (**C**) SDS-PAGE of pathway enzymes in *Escherichia coli* Rosetta (DE3), Blue arrows correspond to bands expressed by different enzymes and are annotated above the bands in red font. (**D**) The total ion chromatograms at different times.

To achieve whole-cell catalytic synthesis of CSE, we implemented a surface display system based on the N-terminal motif of ice nucleoprotein (Fig. S14). To confirm the external localization of INPN-*Ec*CHST15^M7^ on the outer membrane, a whole-cell reaction was conducted with exogenous addition of PAPS for 24 h, resulting in a sulfation efficiency of 58.2%. We hypothesize that the low sulfation efficiency observed in the whole-cell reaction may be attributed to an insufficient supply of PAPS. To address this, the engineered strain P1 (Rosetta DE3-*Kl*ATPS-*Pc*APSK-*Ec*CHST15^M7^-*Rn*ASTIV-*Ec*PPA-*Rs*PPK) constructing an intracellular six-enzyme cascade (Fig. 6B and C), was employed for CSE production. This strain comprises two modules (Fig. 6A), PAPS synthesis module and sulfation module. In module I, PAPS was synthesized using ATP and sulfate as substrates through a two-step reaction. *E. coli* pyrophosphatase (*Ec*PPA) and *Rhodobacter sphaericus* Polyphosphokinase (*Rs*PPK) were introduced to establish an ATP recycling system, improving ATP utilization efficiency. In module II, PAPS and CSA were used as substrates to generate CSE. *Rattus norvegicus* derived sulfotransferase (*Rn*ASTIV) was introduced to catalyze the synthesis of PAPS from PNPS and PAP, thus constructing the PAPS circulatory system. The results revealed that the engineered strain P1 exhibited only a 33.9% conversion of CS, significantly lower than the sulfation efficiency observed *in vitro*. To further improve the sulfation efficiency, we optimized the whole-cell reaction system (Fig. S15). Under optimal conditions (pH 7.5, temperature 37°C, 1.0 g/L Triton X-100, 2 mM glutathione, 5 mM CuCl_2_, 60 mM ATP, 200 mM Na_2_SO_4_, and 100 mM PNPS, 24 h), the sulfation efficiency of 15 g/L CSA was 72.2% (Fig. 6D).

## DISCUSSION

In the present study, we screened the sulfotransferase CHST15, responsible for catalyzing the synthesis of CSE from CSA. CHST15 was heterologously expressed in a prokaryotic organism (*E. coli*), resulting in an enzyme activity of 44.6 U/mL. To enhance sulfation efficiency, we engineered CHST15 based on the structural features and catalytic mechanism, ultimately yielding the optimal mutant M7. The *in vitro* conversion of M7 reached 62.5%, demonstrating a noteworthy 44.4% improvement compared to WT. Finally, we integrated the dual-cofactor cycling system with CHST15, thereby constructing a whole-cell catalyst for CSE synthesis with a remarkable conversion of 72.2%. This approach achieved efficient whole-cell production of CSE. Altogether, this work confirms that sulfotransferases with good catalytic properties can be obtained by protein engineering modifications and that whole-cell biosynthesis of CSE can be achieved using a surface display system. The strategy employed in CS biosynthesis exhibits substantial potential and is anticipated to emerge as a viable alternative to traditional methods of CS production.

In this study, we conducted a comprehensive screening of the sulfotransferase CHST15, responsible for catalyzing the synthesis of CSE from CSA. Notably, we achieved the heterologous expression of CHST15 in a prokaryotic organism, specifically *E. coli* BL21. While the sulfotransferase C4ST, associated with CSA, has been extensively studied, with successful expression in mammalian cells COS and CHO cells (16). Recently, human-derived C4ST was expressed in *Picpastoris* and *E. coli*, with enzyme activities of 46.1 and 25.0 U/mL, respectively (13, 17). However, limited reports exist on sulfotransferase CHST15. Prior studies, such as that by Osami Habuchi et al., involved the transfection of recombinant plasmid pcDNA-CHST15 containing cDNA into COS-7 cells, determining CHST15 activity to be 33.7 U/mL using CSA as substrate (18). In our study, the screening process for CHST15 involved database mining, leading to the successful identification of this sulfotransferase. The enzyme activity of *Ec*CHST15 was measured at 44.6 U/mL. Furthermore, by employing N-terminal truncation expression and incorporating a solubility-enhancing tag, we significantly increased the heterologous expression activity of *Ec*CHST15 by 2.8-fold, reaching 125.1 U/mL. This advancement in heterologous expression contributes to the broader understanding and potential utilization of CHST15 in the biosynthesis of CSE from CSA.

This study improved the catalytic efficiency of CHST15 through protein engineering guided by structural features and catalytic mechanisms. While rational enzyme design based on structural features and catalytic mechanisms has been extensively employed, there is limited literature on protein modification for sulfotransferases involved in CS synthesis. For example, based on the structure of C4ST-1, saturation mutagenesis was performed on L134 and its vicinity in the PSB loop. The resulting double mutant L134E/F184S exhibited an approximately twofold increase in enzyme activity, with a *k*_cat_/*K*_m_ of 12.75 ± 0.41 s^−1^ M^−1^ (13). Due to the poor thermal stability of CHST11, protein engineering was carried out on the PAPS binding gate loop of CHST11, yielding the optimal mutant M12 with a *k*_cat_/*K*_m_ of 15.91 ± 0.24 S^−1^ M^−1^ (17). However, the sulfation of the C-4 and C-6 positions of GalNAc under sulfotransferase action poses challenges, with greater difficulty in substituting the GalNAc bisulfation site compared to the monosulfation site (18). Therefore, inspired by the previous research on sulfotransferase CHST11 catalyzing the C-4 position of GalNAc in our group, we conducted a ligand-binding pocket redesign for CHST15. Based on the hydroxyl sulfation at the C-4 position of GalNAc, the hydroxyl sulfation at C-6 was performed. As a result, the optimal mutant M7 was obtained, exhibiting a *k*_cat_/*K*_m_ increase from 9.06 ± 0.21 to 11.03 ± 0.40 s^−1^ M^−1^. Its specific activity and conversion were enhanced by 2.22-fold and 3.45-fold compared to WT, respectively, with the *in vitro* conversion increased from 18.1% to 62.5%.

Building upon our previous research, we successfully developed a six-enzyme cascade whole-cell catalyst (designated as P1), thereby achieving the biosynthesis of CSE. With the advancement of science and technology, various challenges have been surmounted, marking a notable transition from traditional animal extraction of CS to contemporary CS biosynthesis—a significant trend in the field. The biosynthesis of CSA has already been accomplished in both *P. pastoris* (13) and *E. coli* (17). For instance, in *P. pastoris*, the construction of a dual-functional protein combining C4ST and ASTIV resulted in a remarkable 98% conversion in a 1-L scale reaction system. This system efficiently catalyzed the synthesis of 15 g/L CS to CSA within 24 h. Similarly, in *E. coli*, an 89.5% conversion was achieved within the same timeframe for the synthesis of 10 g/L CS to CSA. However, the biosynthesis of CSE has not been explored. Therefore, inspired by our previous research (12), we introduced an ATP and PAPS dual-cycle module to ensure an abundant supply of APS, successfully constructing a whole-cell catalyst for CSE biosynthesis. The conversion for catalyzing 15 g/L CSA to CSE within 24 h reached 72.2%, marking an efficient production of CSE. Despite being influenced by the varying difficulty of sulfation at different substitution sites (18), the biosynthesis efficiency of CSE is slightly lower than that of CSA. Nonetheless, the successful construction of the whole-cell catalyst represents a significant advancement toward achieving industrial-scale biosynthesis of CSE.

## MATERIALS AND METHODS

### Strains, plasmids, and chemicals

All strains and plasmids are listed in Table S1. Commercial reagents, standards, and solvents were purchased from Sigma-Aldrich (Shanghai, China), Aladdin Reagents (Shanghai, China), and Macklin Reagents (Shanghai, China) and used without further purification. All other chemicals used were of analytical grade. In this study, commercial CSA was obtained from Sigma-Aldrich Reagents, CAS Number: 39455-18-0, molecular formula: (C_14_H_19_NO_14_SNa_2_)*n*, MDL number: MFCD00130792, NACRES:NA.75.

### Construction of plasmids with different solubility tags

Taking the construction of recombinant strain *Ec*CHST15-MBP/BL21 (DE3) as an example, the construction process was as follows: (i) The genome of *E. coli* K12 was employed as a template to amplify the fragment containing MBP, utilizing the primers listed in Table S2. The amplified target fragment was ligated with the linearized plasmid vector pET28a(+)-*Ec*CHST15 using homologous recombinase, with the reaction carried out for 30 min at 37°C. (ii) The recombinant plasmids were introduced into BL21 (DE3) commercial competent cells, followed by cultivation in a constant temperature incubator at 37°C for 8–12 h. (iii) Single colonies were chosen for colony PCR, and the plates with identical resistance were streaked. Colonies with a sequencing band size of approximately 2,500 bp (MBP-*Ec*CHST15) were confirmed via nucleic acid gel electrophoresis. (iv) The recombinant strain pET28a-MBP-*Ec*CHST15/BL21 (DE3) was produced by cultivating colonies with the correct band sizes in lysogeny broth (LB) medium for 8–12 h and subsequently stored in glycerol tubes at −80°C. Other lysogenic tags TrxA, GST, and SUMO were amplified from the genome using the primers listed in Table S2. The construction method mirrored that of the recombinant strain pET28a-MBP-*Ec*CHST15/BL21 (DE3). The recombinant strains were designated as pET28a-TrxA-*Ec*CHST15/BL21 (DE3), pET28a-GST-*Ec*CHST15/BL21 (DE3), and pET28a-SUMO-*Ec*CHST15/BL21 (DE3).

### Enzyme expression and purification

All desired purified proteins and mutants were obtained under the following operating conditions. Strains were initially cultured in LB medium supplemented with appropriate antibiotics at 37°C under constant agitation at 200 rpm. Upon reaching an OD_600_ of 0.6–0.8 in the culture broth, IPTG was introduced to achieve a final concentration of 0.1 mg/mL. The cells were induced at 16°C for 16 h, harvested by centrifugation (5,000 × *g*, 10 min), and subsequently resuspended in lysis buffer (20 mM Tris-HCl [pH 7.4], 20 mM NaCl, and 1 mM EDTA). The cell suspension was disrupted using a high-pressure homogenizer. The resultant crude lysate underwent centrifugation at 10,000 × *g* and 4°C for 30 min. The MBP-tagged protein was captured on dextrin agarose resin for an additional 30 min. Afterward, the elution buffer (20 mM Tris-HCl [pH 7.4], 1 mM EDTA, and 10 mM maltose) was employed to release the captured protein. Protein concentration was determined using the BCA Protein Assay Kit (Solarbio, China). Protein purity was assessed through SDS-PAGE.

### Molecular docking

The CSA disaccharide and PAPS were fully optimized at the B3LYP/6-31G(d) level using the Gaussian 16 (19) package. Subsequently, they were docked into the active site of stable complex structures. Molecular docking was performed with the Lamarckian genetic algorithm local search method using AutoDock Vina (20). The docking approach was applied to the rigid-receptor conformation. A total of 500 independent docking simulations were performed. Subsequently, the binding conformations for MD simulations of *Ec*CHST15-CSA, *Ec*CHST15-PAPS, and *Ec*CHST15-CSA-PAPS were selected based on the scoring function and reasonable conformation.

### MD simulations

The computational experiments consisted of several parts: (i) Empty protein structures of WT and mutants: Utilizing the AlphaFold2 software to predict the three-dimensional structural models of wild-type and mutant *Ec*CHST15 proteins, followed by a 100-ns MD simulation. (ii) MD simulations (15): All MD simulations were performed using the Amber 18 package software. The MD pre-equilibrated *Ec*CHST15 wild-type, its variants, 2IID structures, and possible catalytically active binding modes of CSA disaccharide and PAPS were employed as initial conformations for MD simulations of the protein–ligand complexes. The partial charges for CSA disaccharide and PAPS were determined through HF/6-31G(d) calculations and the restrained electrostatic potential protocol (21), implemented by the Antechamber module in the Amber 18 package. The force field parameters for CSA disaccharide and PAPS were adapted from the standard general amber force field 2.0 (GAFF2) parameters (15), while the standard Amber14SB force field was used to describe the protein. Each system was initially neutralized with Na^+^ counterions and solvated with explicit TIP3P water in a truncated octahedral box with a 10 Å buffer distance. Each system underwent a process of equilibrium through a series of minimizations interspersed by short MD simulations. During this process, restraints on the heavy atoms of the protein backbone were gradually released, with force constants decreasing in steps. Subsequently, the system was slowly heated from 0 to 310K over 50 ps. Finally, a standard unrestrained 100 ns MD simulation was conducted with periodic boundary conditions at 310K and 1 atm (3). Interaction analysis: It primarily encompassed hydrophobic interactions, H-bond interactions, and van der Waals forces (4). Complex structures of mutants with ligands: Initial binary/ternary complex structures of mutants with ligands were constructed by overlapping with the WT complex structure, and stable and accurate conformations of mutants with ligands were obtained by 100 ns MD simulation.

### QM calculations

The representative snapshots extracted from MD trajectories were utilized for subsequent QM calculations. The geometry optimizations, involving minima and transition states, were carried out at the B3LYP/6-31 + G(d,p) level. The vibrational frequency calculations were performed at the same level to ensure that all of the stationary points were transition states (one imaginary frequency) or minima (no imaginary frequency). Additionally, assessments of zero-point vibrational energies (ZPVE) and thermal corrections at 303K were conducted. Single-point energy calculations were performed at the B3LYP level using the 6-311 ++ G(2d,p) basis set. Throughout these computations, solvation by water was considered by using the CPCM model (22–24). Gibbs free energy represents the sum of the electronic energy, ZPVE, and thermal corrections.

### Directed evolution experiments

These mutants were generated using a whole-plasmid polymerase chain reaction (PCR) assay with the primary primers specified in Schedule 2. The PCR system (200 µL) included template (100–120 ng, 4 µL), respective primers (20 µM, 4 µL), KOD enzyme (4 µL), dNTPs (20 µL), Mg^2+^ (16 µL), Buffer (20 µL), and ultrapure water. Subsequently, DpnI was introduced into the PCR reaction mixture and incubated at 37°C for 40 min to remove the template plasmid. The digested products were then introduced into BL21 (DE3) cells for subsequent screening or DNA sequencing conducted by Genewiz, China. Randomly selected individual colonies from the plates were cultured in 200 µL LB medium supplemented with 0.5% (vol/vol) kanamycin sulfate in 96-well plates, agitated at 400 rpm for 12 h at 37°C. Afterward, a 1:5 dilution was made into 800 µL TBA medium [12 g/L tryptone, 24 g/L yeast extract, 5 g/L glycerol, 0.5 g/L glucose, 4 g/L lactose, 3.3 g/L (NH_4_)_2_SO_4_, 6.8 g/L KH_2_PO_4_, 7.1 g/L NaHPO_4_ 12H_2_O, and 0.15 g/L MgSO_4_]. Following incubation at 400 rpm and 37°C for 2–3 h, the temperature was lowered to 16°C for an additional 16 h. The bacterial culture was then centrifuged at high speed, and the resulting pellet was frozen at −80°C. Lysozyme was added to achieve a final concentration of 2.5 mg/mL, and the mixture was placed in a shaker at 16°C for 1 h. A 96-well plate containing 5 mg/mL CSA, 10 mM PAPS, and 1 mM CuCl_2_ was prepared, and the buffer was diluted to a final volume of 500 µL. We used a microplate reader to measure the absorbance at 400 nm every 2 s, monitoring the formation of para-nitrophenol (pNP). The activity of CHST15 is defined by the rate of pNP formation. From each well of the 96-well plate, 50 µL of the mutant fermentation supernatant was taken and mixed with 150 µL of DMMB coloring reagent (containing 3.04 g/L glycine, 2.37 g/L sodium chloride, and 16 mg/L 1,9-dimethylmethylene blue, pH 3.0). The mixture was allowed to react at room temperature for 0–5 min, and the absorbance was measured at a wavelength of 525 nm. Based on the pNP standard curve, beneficial mutants were screened (Fig. S19).

### Kinetic analysis and enzyme activity determination

Kinetic parameters, including *V*_max_, *K*_m_, and *k*_cat_, were determined by assessing the initial rates of product formation at various chondroitin A sulfate concentrations (1–2,000 mM) at 37°C. The reaction system comprised 20 µL of *Ec*CHST15, 50  mM p-nitrophenyl sulfate (pNPS), 0.5  mM PAP, and 20 µL of *Rn*ASTIV. The formation of pNP (p-nitrophenol) was monitored by measuring absorbance at 400  nm every 2  s.

### HPLC analysis

Disaccharide units were detected using strong anion-exchange HPLC and GPC. The sulfonation level of CSE is defined as the ratio of CSE disaccharide (Di-4,6S) content to the combined amount of Di-4,6S and CSA (Di-4S). For reaction samples, the water-insoluble fraction was removed by centrifugation at 12,000 × *g* for 20 min. Thereafter, chondroitinase ABC was added and incubated at 37^o^C for 12 h to ensure complete depolymerization of CSA and CSE. HPLC was conducted using a ZORBAX SAX column (Agilent, 4.6 × 250 mm^2^, 5 µm) at 40^o^C, and detection was carried out with a UV detector at 232  nm. Eluents A and B were 2 M NaCl (pH 3.5) and ultrapure water (pH 3.5), respectively. Gradient separation was performed using eluent A followed by a linear gradient from 0 to 1 M at a flow rate of 1 mL/min for 45 min. GPC was performed by an Ultrahydrogel Linear 7.8 × 300 mm^2^ column at 40^o^C using 0.1 M NaNO_3_ as the eluent at the rate of 0.5 mL/min. The hydrolysate of CS was analyzed by ESI-MS (Thermo, Waltham, MA, USA) in negative ion mode. Mass analysis was conducted over a duration of 15 min. In negative ion mode, the sheath gas flow rate was set at 20 arb, the aux gas flow rate at 5 arb, the spray voltage at 3.5 kV, the capillary temperature at 275^o^C, the capillary voltage at −40 V, and the tube lens at −50 V. The scan range of positron ionization was 100–2,000 *m/z*.

### Molecular weight identification of polysaccharides

The polysaccharide molecular weight results in this study are located in Fig. S13. CSA was dissolved in ultrapure water and filtered through a 0.22-µm membrane. HPLC-SEC was employed, using an Ultrahyfrogel column (300 mm × 7.8 mm i.d., Waters Corporation, Milford, MA, USA) combined with a refractive index detector to measure the molecular weight of CSA. The mobile phase consisted of 0.1 M NaNO_3_ and the flow rate was maintained at 0.9 mL min^−1^.

### Analysis of the composition of CS disaccharides

The disaccharide fractions were determined by 1D ^1^H NMR. Each sample was dissolved in 10% ^2^H2O, and then transferred into an NMR tube. All the NMR experiments were performed at 25°C on a Varian Inova spectrometer at 400 MHz. The 1D ^1^H spectra were recorded using 128 scans with a spectral width of 7  kHz. Acquisition time was set to 2 s and water pre-saturation pulse was set to the position of the carrier for a period equal to the recovery delay, 1.5  s. The disaccharide fractions were dried in a desiccator under a mild vacuum with dry air of less than 3% relative humidity. Approximately 5  mg of the sample was transferred into a differential scanning calorimeter pan and heated to 110°C for several hours to remove residual solvent. Samples were scanned by FTIR (Shimadzu) over the 580–4,000 cm^−1^ range at 5°C/min to study the hydrogen-binding interactions of different chain lengths. The spectral analyses and displays were performed using a Perkin-Elmer workstation.

### Experimental parallel and data processing method

All the analytical experiments in this study were conducted in triplicate under identical conditions, and each reported value represents the average of the results obtained from the three parallel samples. Data analysis was performed using Origin 2022 software.

## Data Availability

All data generated during this study are included in the article and its supplemental material.

## SUPPLEMENTAL MATERIAL

Supplemental material aem.01573-24-s0001.docx Tables S1 to S6; Figures S1 to S20.

ASM does not own the copyrights to Supplemental Material that may be linked to, or accessed through, an article. The authors have granted ASM a non-exclusive, world-wide license to publish the Supplemental Material files. Please contact the corresponding author directly for reuse.
