# Supplementary material for: Structural and mechanism-based engineering of sulfotransferase CHST15 for the efficient synthesis of chondroitin sulfate E
Source: Appl Environ Microbiol. 2024 Dec 4;91(1):e01573-24. doi: 10.1128/aem.01573-24 (PMC11784081; doi:10.1128/aem.01573-24)
Supplement: Supplemental material — Tables S1 to S6; Figures S1 to S20. [file aem.01573-24-s0001.docx]

**Supporting Materials**

**Structural and mechanism based engineering of sulfotransferase CHST15 for the efficient synthesis of chondroitin sulfate E**

*Zhonghua Wang^a^, Wei Song^a^, Wanqing Wei^b,c^, Hejia Qi^b,c^, Weiwei Meng^b,c^, Jia Liu^b,c^, Xiaomin Li^b,c^, Cong Gao^b,c^, Liming Liu^b,c^, Guipeng Hu^a^, Yiwen Zhou^a^, Jing Wu^a *^*

^a^ School of Life Sciences and Health Engineering, Jiangnan University, Wuxi 214122, China.

^b^ School of Biotechnology , Jiangnan University, Wuxi 214122, China.

^c^ Key Laboratory of Industrial Biotechnology of Ministry of Education, Jiangnan University, Wuxi 214122, China.

*Corresponding author.

Jing Wu − School of Life Sciences and Health Engineering, Jiangnan University, Wuxi 214122, China.

Phone: +86-510-85910059;

Email: wujing@jiangnan.edu.cn;

**Table of contents:**

[Supplementary Tables 4](#_Toc24723)

[Table S1 Strains and plasmids. 4](#_Toc21223)

[Table S2 The primers used in this study. 5](#_Toc27810)

[Table S3 Plasmids with different copy numbers were used to express different modules. 10](#_Toc26298)

[Table S4 Mutants obtained from the first round of directed mutagenesis of](#_Toc5702) *[Ec](#_Toc5702)*[CHST15 12](#_Toc5702)

[Table S5 Kinetic parameters of](#_Toc4884) *[Ec](#_Toc4884)*[CHST15 WT and M7 mutants. 14](#_Toc4884)

[Table S6 Sequence similarity of the sulfonate transferase CHST15 from different sources 15](#_Toc2947)

[Supplementary Figures 16](#_Toc4109)

[Fig. S1 CHST15 expression from different sources. 16](#_Toc26817)

[Fig. S2 Expression conversion and enzyme activity of CHST15 from different sources in host bacterium BL21(DE3). 18](#_Toc18765)

[Fig. S3 SDS-PAGE of solubilization label co-expressed with](#_Toc5341) *[Ec](#_Toc5341)*[CHST15. 19](#_Toc5341)

[Fig. S4 Optimization of induced expression conditions. 20](#_Toc8962)

[Fig. S5 AlphaFold prediction of the](#_Toc15555) *[Ec](#_Toc15555)*[CHST15 protein structure 22](#_Toc15555)

[Fig. S6 Analysis of conserved residues of sulfotransferase 25](#_Toc7909)

[Fig. S7 Transition state captured by different models during QM calculations. 26](#_Toc13951)

[Fig. S8 Using GaussView to calculate reaction barriers for 12 different states 27](#_Toc4384)

[Fig. S9 Enzyme protein](#_Toc19103) *[Ec](#_Toc19103)*[CHST15 and small molecule volumes 28](#_Toc19103)

[Fig. S10 Comparison of cofactor cavity volumes between WT and M4 mutants 29](#_Toc30958)

[Fig. S11 Theoretical docking model of](#_Toc30922) *[Ec](#_Toc30922)*[CHST15 with [RC]/[TS3] states 30](#_Toc30922)

[Fig. S12 Comparative analysis of M4 and M7 mutations 31](#_Toc18252)

[Fig. S13 Molecular weight identification of polysaccharides. 32](#_Toc26166)

[Fig. S14 Expression of pET22b-INPN-](#_Toc19481)*[Ec](#_Toc19481)*[CHST15](#_Toc19481)^[M7](#_Toc19481)^ [protein 33](#_Toc19481)

[Fig. S15 Optimization of whole cell response system 34](#_Toc27836)

[Fig. S16 Mutation verification of key residues. 35](#_Toc1371)

[Fig. S17 Hydrogen nuclear magnetic resonance spectrometry (NMR-H) of CSA and CSE 36](#_Toc14233)

[Fig. S18 HPLC diagram of chondroitin and chondroitin sulfate 37](#_Toc10624)

[Fig. S19 The pNP standard curve. 38](#_Toc19669)

[Fig. S20 Time course of reactivity of chondroitinase ABC-I to CSE. 39](#_Toc32448)

[Calculated coordinates 40](#_Toc17290)

# Supplementary Tables

## Table S1 Strains and plasmids.

| Plasmids or strains | Relevant characteristics |
| --- | --- |
| Plasmids |  |
| pET22b | pBR322 ori, AmpR, PT7, 6*His, PelB, TT7 |
| pET28a | pBR322 ori, KanR, PT7, 6*His,TT7 |
| pRSF-Duet-1 | pBR322 ori, KanR, PT7, 6*His, PT7, S-Tag, TT7 |
| pCDF-Duet-1 | pBR322 ori, StrR, PTrc, 6*His, PT7, S-Tag, TT7 |
| pET-Duet-1 | pBR322 ori, AmpR, PTrc, 6*His, PT7, S-Tag, TT7 |
| Strains |  |
| *E. coli* K12 | Used to obtain a solubilizing label |
| *Saccharomyces cerevisiae* | Used to obtain a solubilizing label |
| *E. coli* BL21(DE3) | For enzyme expression and purification |
| *E. coli* Rosetta (DE3) | For enzyme expression and purification |

## **Table S2** The primers used in this study.

| Name | Sequence (5’–3’) |
| --- | --- |
| Primers for Key residue verification | |
| K126A-F | TATTGGTCAGCCGGCGGGGGGCACCACCG |
| K126A-R | CGGTGGTGCCCCCCGCCGGCTGACCAATA |
| K126R-F | TATTGGTCAGCCGCGCGGGGGCACCACCG |
| K126R-F | CGGTGGTGCCCCCCGCCGGCTGACCAATA |
| K126E-F | TATTGGTCAGCCGGAAGGGGGCACCACCG |
| K126E-R | CGGTGGTGCCCCCTTCCGGCTGACCAATA |
| T130A-F | GAAAGGGGGCACCGCGGATCTGTATGATC |
| T130A-R | GATCATACAGATCCGCGGTGCCCCCTTTC |
| R148A-F | ATTTAGCGCGATTGCGGAACCGCATTGGT |
| R148A-R | ACCAATGCGGTTCCGCAATCGCGCTAAAT |
| R148K-F | ATTTAGCGCGATTAAAGAACCGCATTGGT |
| R148K-R | ACCAATGCGGTTCTTTAATCGCGCTAAAT |
| R148E-F | ATTTAGCGCGATTCAAGAACCGCATTGGT |
| R148E-R | ACCAATGCGGTTCTTCAATCGCGCTAAAT |
| H151A-F | GATTAAAGAACCGGCGTGGTGGACCCGCA |
| H151A-R | TGCGGGTCCACCACGCCGGTTCTTTAATC |
| K270A-F | TGCGAGCAGCAACGCGAGCGCGGAAGATT |
| K270A-R | AATCTTCCGCGCTCGCGTTGCTGCTCGCA |
| E256A-F | GCGCGATCCGGTGGCGCGCCTGTATAGCG |
| E256A-R | CGCTATACAGGCGCGCCACCGGATCGCGC |
| R396A-F | GGCGAGCAGCGGCGCGCGTCCGGAAGACC |
| R396A-R | GGTCTTCCGGACGCGCGCCGCTGCTCGCC |
| R397A-F | GAGCAGCGGCCGCGCGCCGGAAGACCGCA |
| R397A-R | TGCGGTCTTCCGGCGCGCGGCCGCTGCTC |
| E399A-F | CGGCCGCCGTCCGGCGGACCGCAGCCTGG |
| E399A-R | CCAGGCTGCGGTCCGCCGGACGGCGGCCG |
| D400A-F | CCGCCGTCCGGAAGCGCGCAGCCTGGGCC |
| D400A-R | GGCCCAGGCTGCGCGCTTCCGGACGGCGG |
| Primers for the library of directed mutation | |
| C127A-F | TGGTCAGCCGAAAGCGGGCACCACCGATC |
| C127A-R | GATCGGTGGTGCCCGCTTTCGGCTGACCA |
| C127G-F | TGGTCAGCCGAAAGGCGGCACCACCGATC |
| C127G-R | GATCGGTGGTGCCGCCTTTCGGCTGACCA |
| C127S-F | TGGTCAGCCGAAAAGCGGCACCACCGATC |
| C127S-R | GATCGGTGGTGCCGCTTTTCGGCTGACCA |
| G128S-F | TGGTCAGCCGAAAAGCGGCACCACCGATC |
| G128S-R | GATCGGTGGTGCCGCTTTTCGGCTGACCA |
| T129G-F | GCCGAAAGGGGGCGGCACCGATCTGTATG |
| T129G-R | CATACAGATCGGTGCCGCCCCCTTTCGGC |
| T129S-F | GCCGAAAGGGGGCAGCACCGATCTGTATG |
| T129S-R | CATACAGATCGGTGCTGCCCCCTTTCGGC |
| T130A-F | GAAAGGGGGCACCGCGGATCTGTATGATC |
| T130A-R | GATCATACAGATCCGCGGTGCCCCCTTTC |
| T130S-F | GAAAGGGGGCACCAGCGATCTGTATGATC |
| T130S-R | GATCATACAGATCGCTGGTGCCCCCTTTC |
| R135K-F | CGATCTGTATGATAAACTGCGCCTGCATC |
| R135K-R | GATGCAGGCGCAGTTTATCATACAGATCG |
| S260G-F | GGAACGCCTGTATGGCGATTATCTGAGCT |
| S260G-R | AGCTCAGATAATCGCCATACAGGCGTTCC |
| L263A-F | GTATAGCGATTATGCGAGCTTTGCGAGCA |
| L263A-R | TGCTCGCAAAGCTGCGATAATCGCTATAC |
| L263G-F | GTATAGCGATTATGGCAGCTTTGCGAGCA |
| L263G-R | TGCTCGCAAAGCTGCCATAATCGCTATAC |
| Y264A-F | TAGCGATTATCTGGCGTTTGCGAGCAGCA |
| Y264A-R | TGCTGCTCGCAAACGCCAGATAATCGCTA |
| Y264G-F | TAGCGATTATCTGGGCTTTGCGAGCAGCA |
| Y264G-R | TGCTGCTCGCAAAGCCCAGATAATCGCTA |
| Y264S-F | TAGCGATTATCTGAGCTTTGCGAGCAGCA |
| Y264S-R | TGCTGCTCGCAAAGCTCAGATAATCGCTA |
| S390A-F | GTTAATGACGCGCGCGCCGGCGAGCAGCG |
| S390A-R | CGCTGCTCGCCGGCGCGCGCGTCATTAAC |
| S390G-F | GTTAATGACGCGCGGCCCGGCGAGCAGCG |
| S390G-R | CGCTGCTCGCCGGGCCGCGCGTCATTAAC |
| P391A-F | AATGACGCGCAGCGCGGCGAGCAGCGGCC |
| P391A-R | GGCCGCTGCTCGCCGCGCTGCGCGTCATT |
| P391G-F | AATGACGCGCAGCGGCGCGAGCAGCGGCC |
| P391G-R | GGCCGCTGCTCGCGCCGCTGCGCGTCATT |
| P391S-F | AATGACGCGCAGCAGCGCGAGCAGCGGCC |
| P391S-R | GGCCGCTGCTCGCGCTGCTGCGCGTCATT |
| A392G-F | GACGCGCAGCCCGGGCAGCAGCGGCCGCC |
| A392G-R | GGCGGCCGCTGCTGCCCGGGCTGCGCGTC |
| S393A-F | GCGCAGCCCGGCGGCGAGCGGCCGCCGTC |
| S393A-R | GACGGCGGCCGCTCGCCGCCGGGCTGCGC |
| S393G-F | GCGCAGCCCGGCGGGCAGCGGCCGCCGTC |
| S393G-R | GACGGCGGCCGCTCCGCGCCGGGCTGCGC |
| N394A-F | CAGCCCGGCGAGCGCGGGCCGCCGTCCGG |
| N394A-R | CCGGACGGCGGCCCGCGCTCGCCGGGCTG |
| N394G-F | CAGCCCGGCGAGCGGCGGCCGCCGTCCGG |
| N394G-R | CCGGACGGCGGCCGCCGCTCGCCGGGCTG |
| N394S-F | CAGCCCGGCGAGCAGCGGCCGCCGTCCGG |
| N394S-R | CCGGACGGCGGCCGCTGCTCGCCGGGCTG |
| A395G-F | CCCGGCGAGCAGCGGCCGCCGTCCGGAAG |
| A395G-R | CTTCCGGACGGCGGCCGCTGCTCGCCGGG |
| C127K-F | TGGTCAGCCGAAAAAAGGCACCACCGATC |
| C127K-R | GATCGGTGGTGCCTTTTTTCGGCTGACCA |
| C127R-F | TGGTCAGCCGAAACGCGGCACCACCGATC |
| C127R-R | GATCGGTGGTGCCGCGTTTCGGCTGACCA |
| C127H-F | TGGTCAGCCGAAACATGGCACCACCGATC |
| C127H-R | GATCGGTGGTGCCGTATTTCGGCTGACCA |
| E256K-F | GCGCGATCCGGTGAAACGCCTGTATAGCG |
| E256K-R | CGCTATACAGGCGTTTCACCGGATCGCGC |
| E256R-F | GCGCGATCCGGTGCGTCGCCTGTATAGCG |
| E256R-R | CGCTATACAGGCGGCACACCGGATCGCGC |
| E256H-F | GCGCGATCCGGTGGATCGCCTGTATAGCG |
| E256H-R | CGCTATACAGGCGGTACACCGGATCGCGC |
| L263K-F | GTATAGCGATTATAAAAGCTTTGCGAGCA |
| L263K-R | TGCTCGCAAAGCTTTTATAATCGCTATAC |
| L263R-F | GTATAGCGATTATCGCAGCTTTGCGAGCA |
| L263R-R | TGCTCGCAAAGCTGCGATAATCGCTATAC |
| L263H-F | GTATAGCGATTATCATAGCTTTGCGAGCA |
| L263H-R | TGCTCGCAAAGCTGTAATAATCGCTATAC |
| Y264K-F | TAGCGATTATCTGAATTTGCGAGCAGCA |
| Y264K-R | TGCTGCTCGCAAATTTCAGATAATCGCTA |
| Y264R-F | TAGCGATTATCTGCGCTTTGCGAGCAGCA |
| Y264R-R | TGCTGCTCGCAAAGCGCAGATAATCGCTA |
| Y264H-F | TAGCGATTATCTGCATTTTGCGAGCAGCA |
| Y264H-R | TGCTGCTCGCAAAGTACAGATAATCGCTA |
| A392K-F | GACGCGCAGCCCGAAAAGCAGCGGCCGCC |
| A392K-R | GGCGGCCGCTGCTTTTCGGGCTGCGCGTC |
| A392R-F | GACGCGCAGCCCGCGCAGCAGCGGCCGCC |
| A392R-R | GGCGGCCGCTGCTGCGCGGGCTGCGCGTC |
| A392H-F | GACGCGCAGCCCGCATAGCAGCGGCCGCC |
| A392H-R | GGCGGCCGCTGCTGTACGGGCTGCGCGTC |
| N394K-F | CAGCCCGGCGAGCAAAGGCCGCCGTCCGG |
| N394K-R | CCGGACGGCGGCCTTTGCTCGCCGGGCTG |
| N394R-F | CAGCCCGGCGAGCCGCGGCCGCCGTCCGG |
| N394R-R | CCGGACGGCGGCCGCGGCTCGCCGGGCTG |
| N394H-F | CAGCCCGGCGAGCCATGGCCGCCGTCCGG |
| N394H-R | CCGGACGGCGGCCGTAGCTCGCCGGGCTG |
| Primers for the library of saturation mutation | |
| C127-F | TGGTCAGCCGAAANNKGGCACCACCGATC |
| C127-R | GATCGGTGGTGCCMNNTTTCGGCTGACCA |
| T129-F | GCCGAAAGGGGGCNNKACCGATCTGTATG |
| T129-R | CATACAGATCGGTMNNGCCCCCTTTCGGC |
| L263-F | GTATAGCGATTATNNKAGCTTTGCGAGCA |
| L263-R | TGCTCGCAAAGCTMNNATAATCGCTATAC |
| Y264-F | TAGCGATTATCTGNNKTTTGCGAGCAGCA |
| Y264-R | TGCTGCTCGCAAAMNNCAGATAATCGCTA |
| A392-F | GACGCGCAGCCCGNNKAGCAGCGGCCGCC |
| A392-F | GGCGGCCGCTGCTMNNCGGGCTGCGCGTC |
| N394-F | CCGGACGGCGGCCNNKGCTCGCCGGGCTG |
| N394-R | CAGCCCGGCGAGCMNNGGCCGCCGTCCGG |
| Q124-F | CTTTTATATTATTGNNKAGCCGAAAGGGG |
| Q124-R | CCCCTTTCGGCTGAMNNATAATATAAAAG |
| P125-F | TATTATTGGTCAGCNNKAAGGGGGCACCA |
| P125-R | TGGTGCCCCCTTTCMNNTGACCAATAATA |
| A209-F | CGGCGAAGCGAGCNNKAGCACCATGGGCG |
| A209-R | CGCCCATGGTGCTMNNGCTCGCTTCGCCG |
| S210-F | CGAAGCGAGCGCGNNKACCATGGGCGCGA |
| S210-R | TCGCGCCCATGGTMNNCGCGCTCGCTTCG |
| W213-F | CGCGAGCACCATGNNKGCGAACAACGCGT |
| W213-R | ACGCGTTGTTCGCMNNCATGGTGCTCGCG |
| D214-F | GAGCACCATGGGCNNKAACAACGCGTGGA |
| D214-R | TCCACGCGTTGTTMNNGCCCATGGTGCTC |
| L163-F | TGGCATTGTGCGCNNKCGCGATGGCCTGC |
| L163-R | GCAGGCCATCGCGMNNGCGCACAATGCCA |
| F324-F | ATGGAACAGCGCGNNKCCGGTGCAAGTGC |
| F324-R | GCACTTGCACCGGMNNCGCGCTGTTCCAT |
| P325-F | GAACAGCGCGTTTNNKGTGCAAGTGCGCC |
| P325-R | GGCGCACTTGCACMNNAAACGCGCTGTTC |
| W304-F | GGCGCGCCCGAGTNNKGGTCTGGCGCCGG |
| W304-R | CCGGCGCCAGACCMNNACTCGGGCGCGCC |
| N216-F | CATGGGCGCGAACNNKGCGTGGACCTTTT |
| N216-R | AAAAGGTCCACGCMNNGTTCGCGCCCATG |
| Primers for the molecular chaperones | |
| MBP-F | GACTGGTGGACAAATGGGTCGCATGAAAATCGAAGAAGGTAAACTGG |
| MBP-R | GGCGGTGGGGATCCGGATCCACTACCTCCGCCGCCAGTCTGCGCGTCTTTCAGGGCTTCA |
| TrxA-F | GACTGGTGGACAGCAAATGGGTCGCATGAGCGATAAAATTATTCACCTGA |
| TrxA-R | CGGCGGTGGGGATCCGGATCCACTACCTCCGCCGCCGGCCAGGTTAGCGTCGAGGAACTC |
| GST-F | GACTGGTGGACAGCAAATGGGTCGCATGTCCCCTATACTAGGTTATTGGA |
| GSE-R | CGGCGGTGGGGATCCGGATCCACTACCTCCGCCGCCTTTTGGAGGATGGTC |
| SUMO-F | GACTGGTGGACAGCAAATGGGTCGCCATATGGCTAGCATGTCGGACTCAG |
| SUMO-R | CGGCGGTGGGGATCCGGATCCACTACCTCCGCCGCCACCAATCTGTTCTCT |

## Table S3 Plasmids with different copy numbers were used to express different modules.

| Modules | Different plasmid combinations |
| --- | --- |
| Main path module | pRSF-Duet-*Pc*APSK-*Rs*PPK |
|  | pET22b (+) -INPN-*Ec*CHST15 |
| ATP cycle path module | pCDF-Duet-*Kl*ATPS-*Ec*PPA |
| PAPS cycle path module | pET-Duet-INPN-*Ec*CHST15-TrxA-*Rn*ASTIV |

## Table S4 Mutants obtained from the first round of directed mutagenesis of *Ec*CHST15

| Round | Methodology | Site of mutation | Template | No. of mutants screened | Beneficial mutants | Conversion(%) | Fold improvement to WT |
| --- | --- | --- | --- | --- | --- | --- | --- |
| - | - | - | - | - | Wild-type | 18.1 | 1.0 |
| 1 | SDM^[a]^ | C127,G128,T129,T130,R135,S260,L263,Y264 | WT | 15 | T129G | 20.2 | 1.1 |
|  |  |  |  |  | T129A | 23.9 | 1.3 |
|  |  |  |  |  | L263A | 20.1 | 1.1 |
|  |  |  |  |  | Y264S | 23.7 | 1.3 |
|  | SDM^[b]^ | S390,P391,A392,S393,  N394,A395 |  | 12 | N394S | 28.3 | 1.6 |
|  |  |  |  |  | N394G | 25.1 | 1.4 |
|  |  |  |  |  | N394A | 25.7 | 1.4 |
|  |  |  |  |  | **A395G**  **(M1)** | 29.6 | 1.6 |
|  | SDM^[c]^ | C127,E256,  L263,Y264,  A392,N394 |  | 18 | C127K | 21.0 | 1.2 |
|  |  |  |  |  | A392R | 21.8 | 1.2 |
| 2 | SSM | T129,C127,L263,Y264,A392,N394 | M1 | 6 | M1/C127A | 36.7 | 2.0 |
|  |  |  |  |  | M1/T129K | 34.5 | 1.9 |
|  |  |  |  |  | M1/L263A | 33.5 | 1.9 |
|  |  |  |  |  | M1/Y264G | 35.1 | 1.9 |
|  |  |  |  |  | M1/A392G | 38.4 | 2.1 |
|  |  |  |  |  | **M1/N394S**  **(M2)** | 41.9 | 2.3 |
| 3 | SSM | T129,C127,L263,Y264,A392 | M2 | 3 | M2/C127G | 42.6 | 2.4 |
|  |  |  |  |  | **M2/Y264S**  **(M3)** | 45.3 | 2.5 |
|  |  |  |  |  | M2/A392G | 43.5 | 2.4 |
| 4 | SSM | C127,A392 | M3 | 1 | **M3/C127G**  **(M4)** | 48.1 | 2.7 |
| 5 | SSM | Q124,P125,A209,S210,W213,D214,L163,F324,P325,W304,N216 | M4 | 6 | M4/W304A | 53.3 | 2.9 |
|  |  |  |  |  | M4/W304R | 50.7 | 2.8 |
|  |  |  |  |  | M4/W304G | 49.5 | 2.7 |
|  |  |  |  |  | **M4/W213G**  **(M5)** | 54.5 | 3.0 |
|  |  |  |  |  | M4/W213A | 49.7 | 2.7 |
|  |  |  |  |  | M4/D214N | 53.4 | 3.0 |
| 6 | SSM | N214,W304 | M5 | 7 | **M5/D214A**  **(M6)** | 59.9 | 3.3 |
|  |  |  |  |  | M5/D214N | 56.0 | 3.1 |
|  |  |  |  |  | M5/D214G | 55.6 | 3.1 |
|  |  |  |  |  | M5/W304A | 57.4 | 3.2 |
|  |  |  |  |  | M5/W304R | 55.4 | 3.1 |
|  |  |  |  |  | M5/W304G | 55.0 | 3.0 |
|  |  |  |  |  | M5/W304K | 56.1 | 3.1 |
| 7 | SSM | W304 | M6 | 4 | M6/304A | 59.6 | 3.3 |
|  |  |  |  |  | **M6/304R**  **(M7)** | 62.5 | 3.5 |
|  |  |  |  |  | M6/304K | 57.6 | 3.2 |
|  |  |  |  |  | M6/304P | 56.5 | 3.1 |

[a] Large amino acid residues within 5 Å of the cofactor were mutated to small amino acid residues. [b] The rigid residues in the gate ring were mutated to flexible residues. [c] Nonconserved residues within the 5 Å range of the cofactor were mutated to positively charged residues.The data represent mean ± SD, as determined from three independent experiments.

## **Table S5** Kinetic parameters of *Ec*CHST15 WT and M7 mutants.

|  | Specific  activity(U·g^-1^) | *K_m_*(mM) | *k_cat_*(s^-1^) | *k_cat_*/*K_m_*(s^-1^·M^-1^) |
| --- | --- | --- | --- | --- |
| WT | 67.41 ± 1.71 | 7.11 ± 0.10 | 0.068 ± 0.001 | 9.56 ± 0.21 |
| **M4^A395G/N394S/Y264S/C127G^** | 120.61 ± 3.52 | 5.06 ± 0.06 | 0.053 ± 0.001 | 10.47 ± 0.19 |
| **M7^A395G/N394S/Y264S/C127G^**  **^/W213G/N214A/W304R^** | 149.53 ± 2.41 | 4.41 ± 0.06 | 0.047 ± 0.001 | 11.03± 0.40 |

Note: The data represent mean ± SD, as determined from three independent experiments. Abbreviation: WT, wild-type.

## **Table S6** Sequence similarity of the sulfonate transferase CHST15 from different sources

| **Enzymes** | **Organisms** | **Sequence similarity** |
| --- | --- | --- |
| *Hs*CHST15 | *Homo sapiens* | 100.0% |
| *Mm*CHST15 | *Mus musculus* | 89.3% |
| *Ef*CHST15 | *Eptesicus fuscus* | 79.1% |
| *Ec*CHST15 | *Erpetoichthys calabaricus* | 75.2% |
| *Lc*CHST15 | *Larimichthys crocea* | 67.7% |
| *Ph*CHST15 | *Podila humilis* | 58.3% |
| *Pv*CHST15 | *Podila verticillata* | 50.2% |
| *Ts*CHST15 | *Tetraselmis sp. GSL018* | 43.9% |
| *At*CHST15 | *Acidithiobacillus thiooxidans ATCC 19377* | 32.1% |

# Supplementary Figures


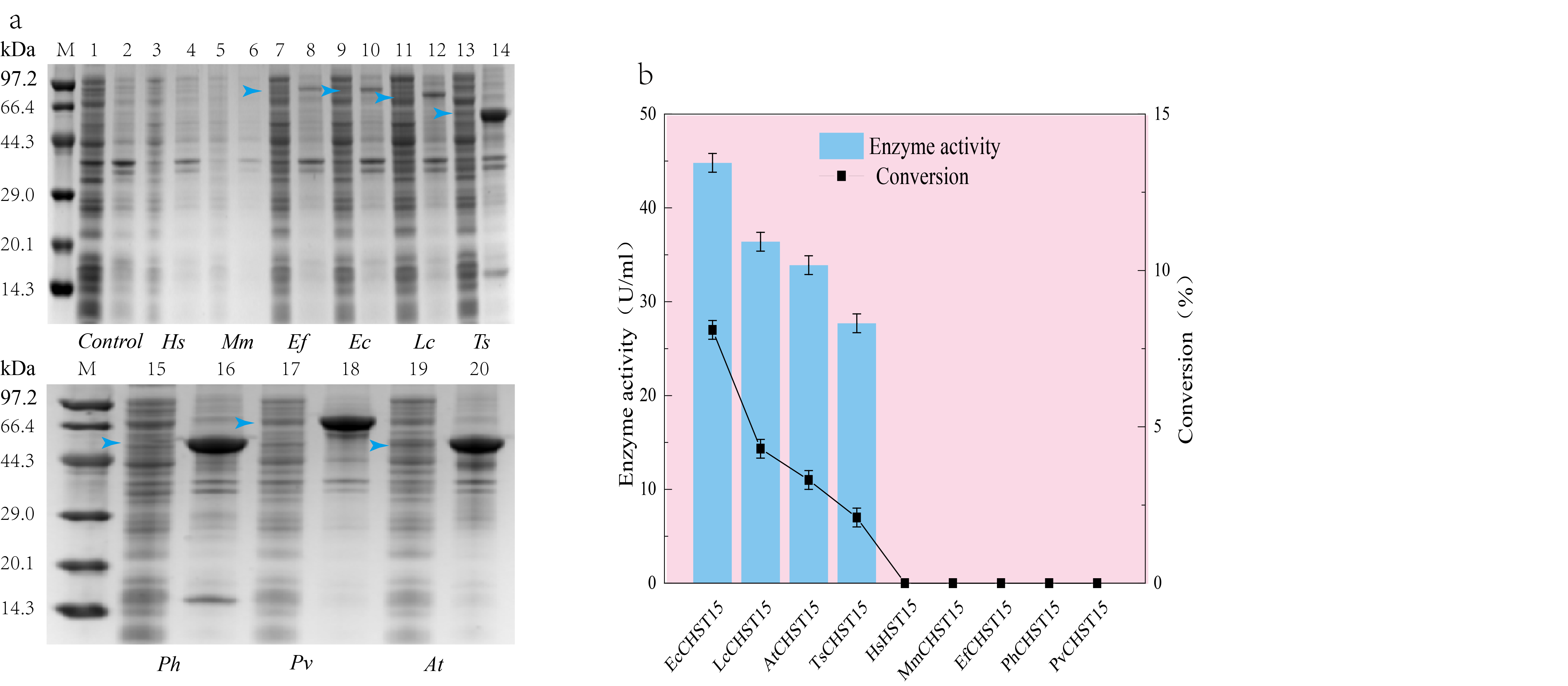


Fig. S1 CHST15 expression from different sources. M, marker; Lane 1, 2,supernatant and precipitation of strain BL21(DE3); Lane 3, 4, supernatant and precipitation of BL21(DE3) cell lysates expressing *Hs*CHST15(*[Homo sapiens](https://www.uniprot.org/taxonomy/9606" \o "Homo sapiens (Human), taxon ID 9606)* ); Lane 5, 6, supernatant and precipitation of BL21(DE3) cell lysates expressing *Mm*CHST15(*Mus musculus*); Lane 7 and 8, superlyant and precipitation of BL21(DE3) cell lysates expressing *Ef*CHST15(*Eptesicus fuscus*); Lane 9, 10, supernatant and precipitation of BL21(DE3) cell lysates expressing *Ec*CHST15(*E calabaricus*); Lane 11, 12, supernatant and precipitation of BL21(DE3) cell lysates expressing *Lc*CHST15(*Larimichthys crocea*); Lane 13, 14, supernatant and precipitation of BL21(DE3) cell lysates expressing *Ts*CHST15(*Tetraselmis sp.GSL018*); Lane 15, 16, supernatant and precipitation of BL21(DE3) cell lysates expressing *Ph*CHST15(*Podila humilis*); Lane 17, 18, supernatant and precipitation of BL21(DE3) cell lysates expressing *Pv*CHST15(*Podila verticillata*); Lane 19, 20, supernatant and precipitation of BL21(DE3) cell lysate expressing *At*CHST15(*Acidithiobacillus thiooxidans ATCC 19377*).


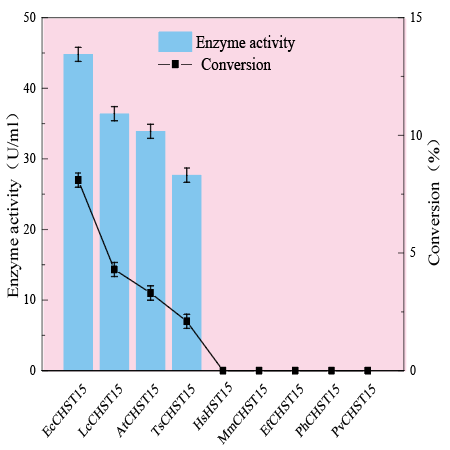


## **Fig. S2** **Expression conversion and enzyme activity of CHST15 from different sources in host bacterium BL21(D**E3).


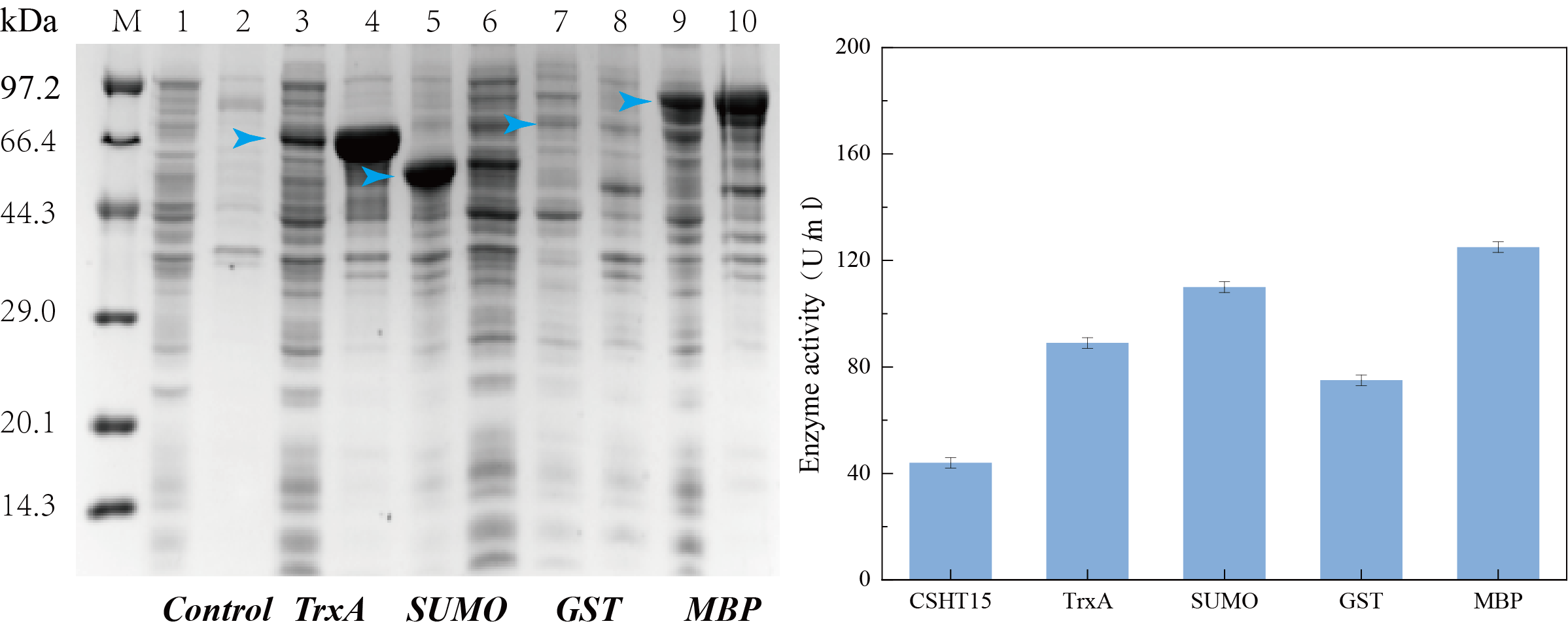


Fig. S3 SDS-PAGE of solubilization label co-expressed with *Ec*CHST15.M，marker;Lane 1,2,Supernatants and precipitates of BL21(DE3) cell lysates expressing *Ec*CHST15;Lane 3,4,Supernatants and precipitates of BL21(DE3) cell lysates expressing TrxA-*Ec*CHST15;Lane 5,6,Supernatants and precipitates of BL21(DE3) cell lysates expressing SUMO-*Ec*CHST15;Lane 7,8,Supernatants and precipitates of BL21(DE3) cell lysates expressing SUMO-*Ec*CHST15;Lane 9,10,Supernatants and precipitates of BL21(DE3) cell lysates expressing MBP-*Ec*CHST15.


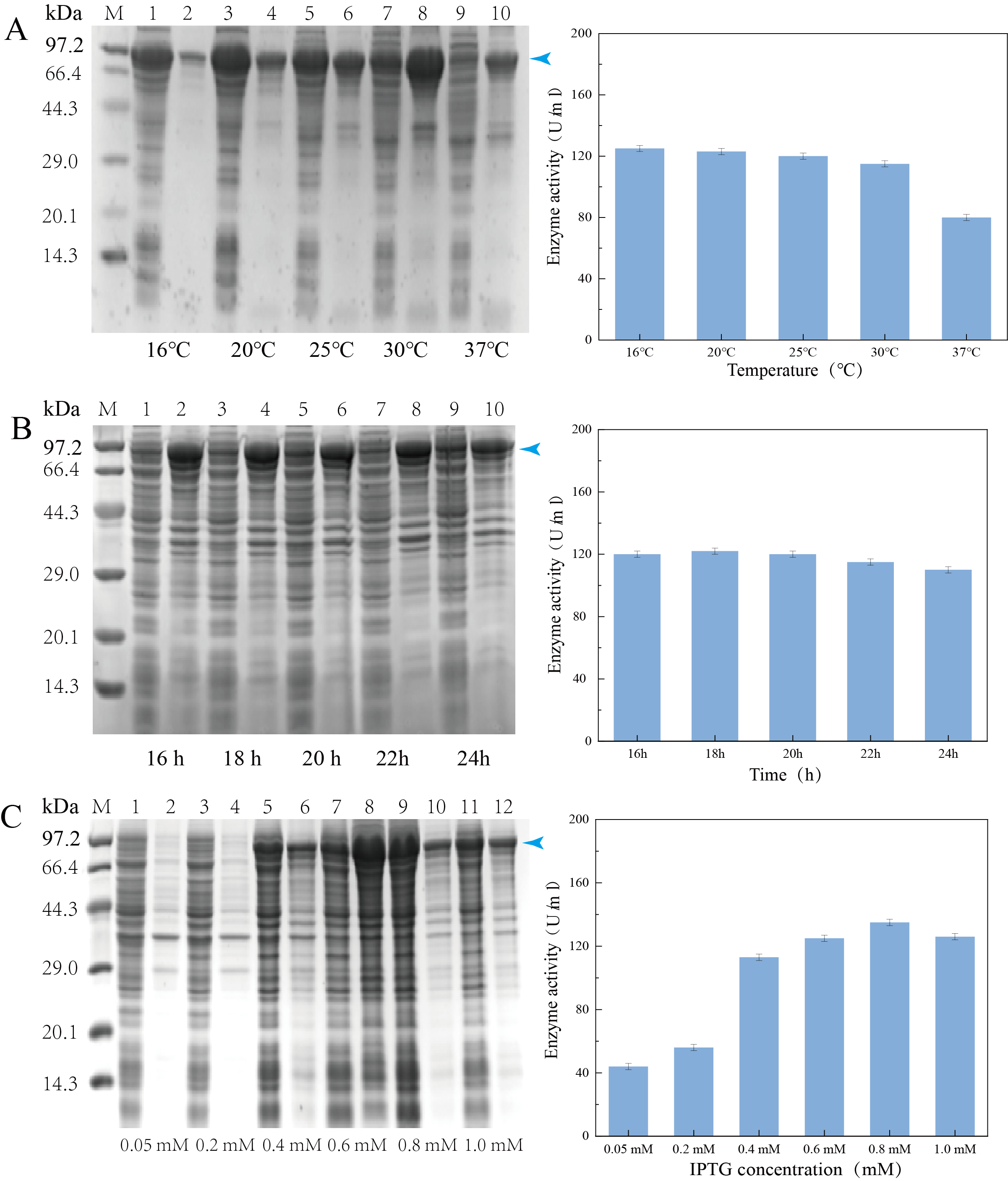


Fig. S4 Optimization of induced expression conditions.(A) Expression of *Ec*CHST15 at different induction temperatures (Fixed induction time is 16 hours, with an inducer concentration of 0.4 mM.); M, marker; Lane 1,2,supernatants and precipitates of BL21(DE3) cell lysates expressing *Ec*CHST15 at 16℃; Supernatants and precipitates of BL21(DE3) cell lysates expressing *Ec*CHST15 at lane 3 and 4 at 20℃; Supernatants and precipitates of BL21(DE3) cell lysates expressing *Ec*CHST15 at lane 5 and 6 at 25℃; Supernatants and precipitates of BL21(DE3) cell lysates expressing *Ec*CHST15 at 30℃ in lane 7 and 8; Supernatants and precipitates of BL21(DE3) cell lysates expressing *Ec*CHST15 in lane 9 and 10 at 37℃; (B) Expression of *Ec*CHST15 at different induction times (Fixed induction temperature is 16 ℃, with an inducer concentration of 0.4 mM.); M, marker; Lane 1 and 2, the precipitate and supernatant of BL21(DE3) cell lysates induced to express *Ec*CHST15 for 16h; Lane 3 and 4, the precipitate and supernatant of BL21(DE3) cell lysates expressing *Ec*CHST15 for 18h were induced; Lane 5 and 6, the precipitate and supernatant of BL21(DE3) cell lysates induced to express *Ec*CHST15 for 20h; Lane 7 and 8, the precipitate and supernatant of lysates from BL21(DE3) cells expressing *Ec*CHST15 for 22h were induced; Lane 9 and 10, the precipitate and supernatant of BL21(DE3) cell lysates induced to express *Ec*CHST15 for 24h; (C) Expression of *Ec*CHST15 at different induction concentrations (Fixed induction temperature is 16 ℃, with an induction time of 18 hours.); M, marker; Supernatants and precipitates of 0.05 mM IPTG expressing *Ec*CHST15 BL21(DE3) cell lysates from lane 1 and 2; Supernatants and precipitates of BL21(DE3) cell lysates expressing *Ec*CHST15 in lanes 3 and 4,0.2 mM IPTG; Supernatants and precipitates of BL21(DE3) cell lysates expressing *Ec*CHST15 at 0.4 mM IPTG in lanes 5 and 6; Supernatant and precipitate of 0.6 mM IPTG BL21(DE3) cell lysates expressing *Ec*CHST15 in lanes 7 and 8; Lanes 9 and 10 supernatants and precipitates of BL21(DE3) cell lysates expressing *Ec*CHST15 at 0.8 mM IPTG; Supernatants and precipitates of BL21(DE3) cell lysates from 1.0 mM IPTG expressing *Ec*CHST15 in lanes 11 and 12;


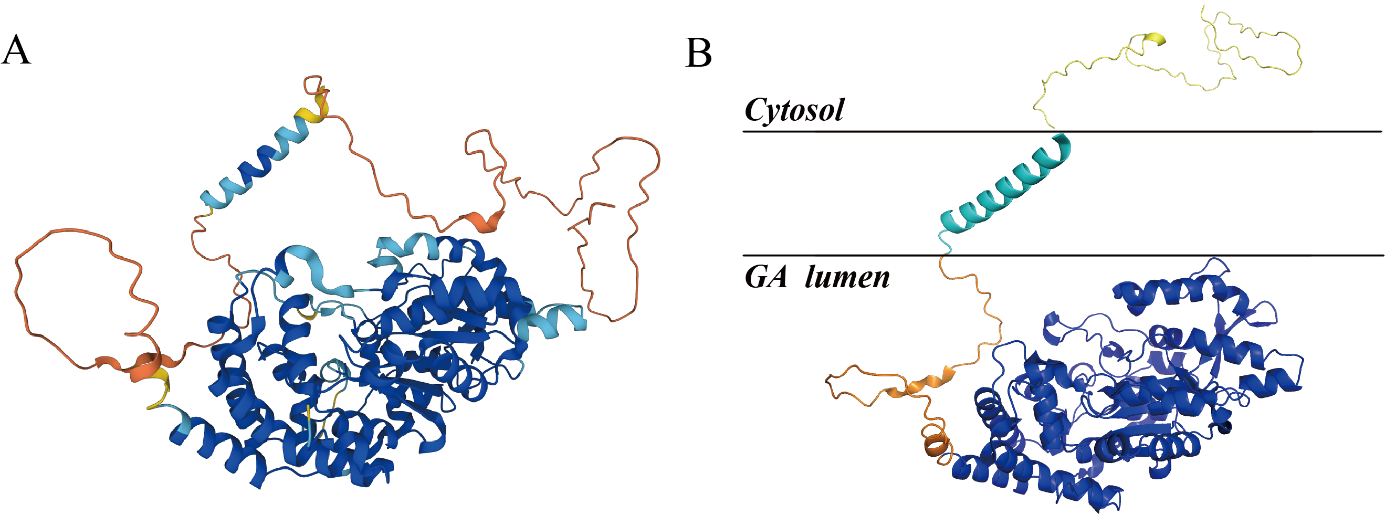


Fig. S5 AlphaFold prediction of the *Ec*CHST15 protein structure. (A) Local Distance Difference Test (pLDDT) predicted by AlphaFold. (Deep blue, pLDDT > 90; Light blue, pLDDT 90-70; Yellow, pLDDT 70-50; Orange, < 50. AlphaFold generates a confidence score (pLDDT) for each residue ranging from 0 to 100, where higher pLDDT indicates higher confidence.) (B) Structural domain classification based on the AlphaFold2-predicted *Ec*CHST15 structure. (Yellow, topological domain; Cyan, transmembrane domain; Orange, stem region in the catalytic domain; Deep blue, catalytic domain)


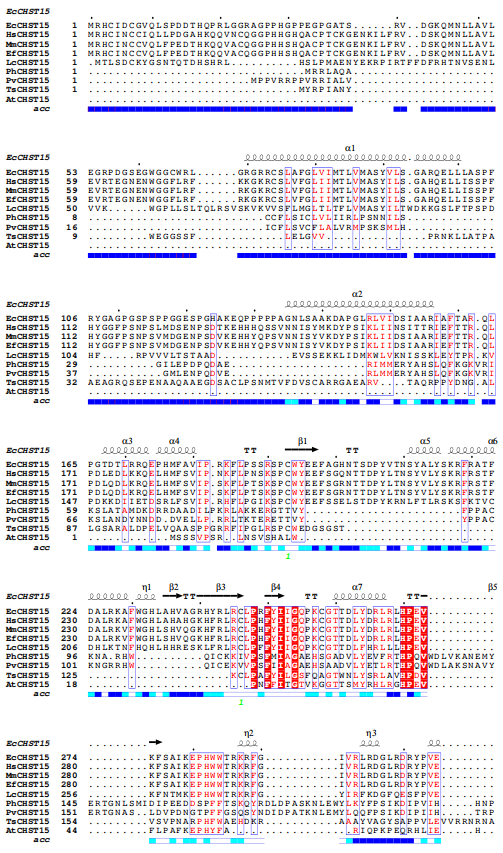


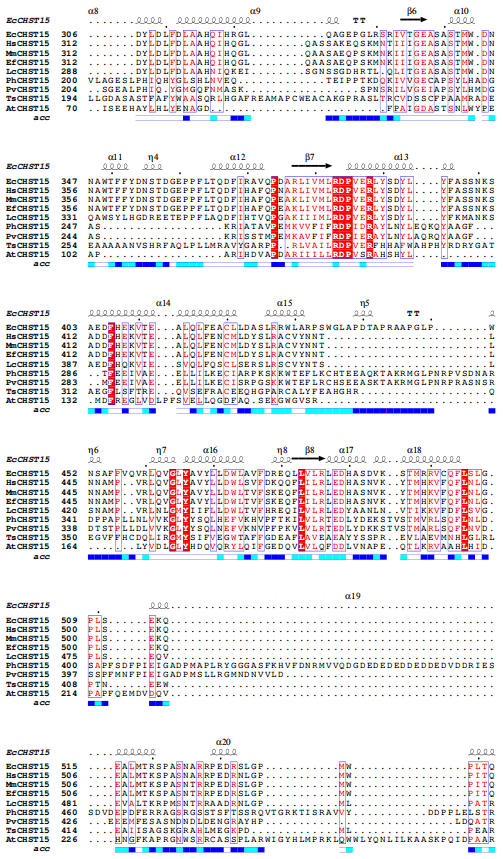


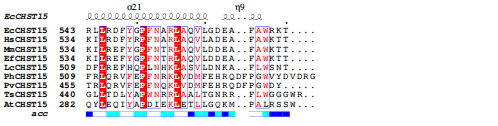


Fig. S6 Analysis of conserved residues of sulfotransferase.

**
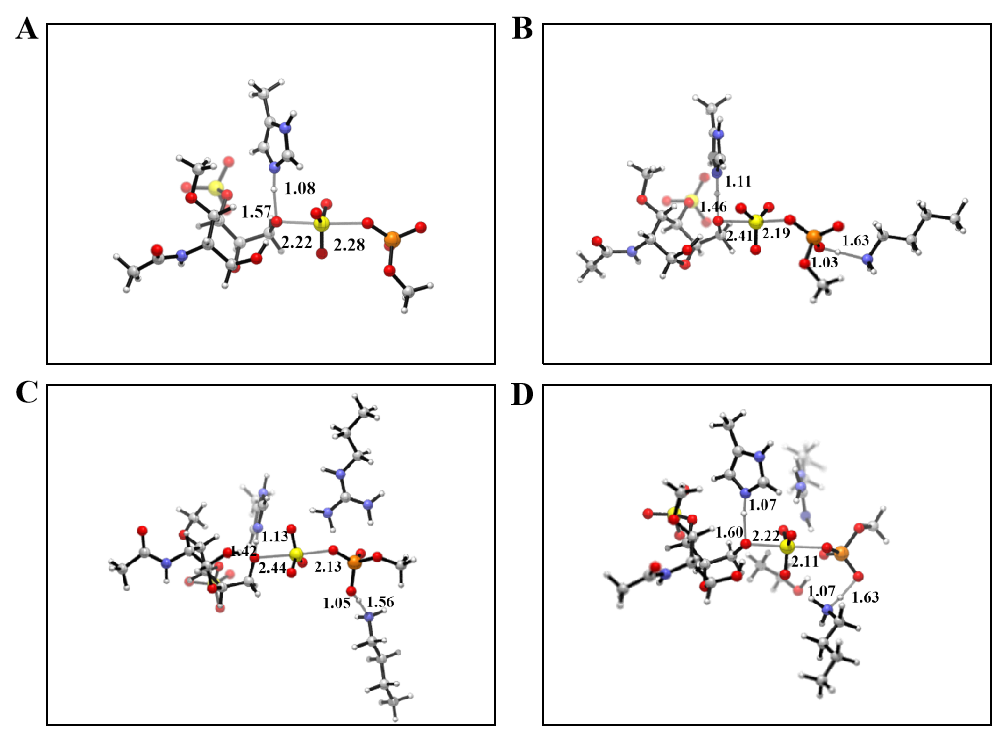
**

Fig. S7 Transition state captured by different models during QM calculations. (A) TS0 captured in the **model 1** (ground state model); (B) TS1 captured in the **model 2** (ground state + LYS model); (c) TS2 captured in the **model 3** (ground state + LYS + ARG model); (D) TS3 captured in the **model 4 (**ground state + LYS + ARG + THR model).


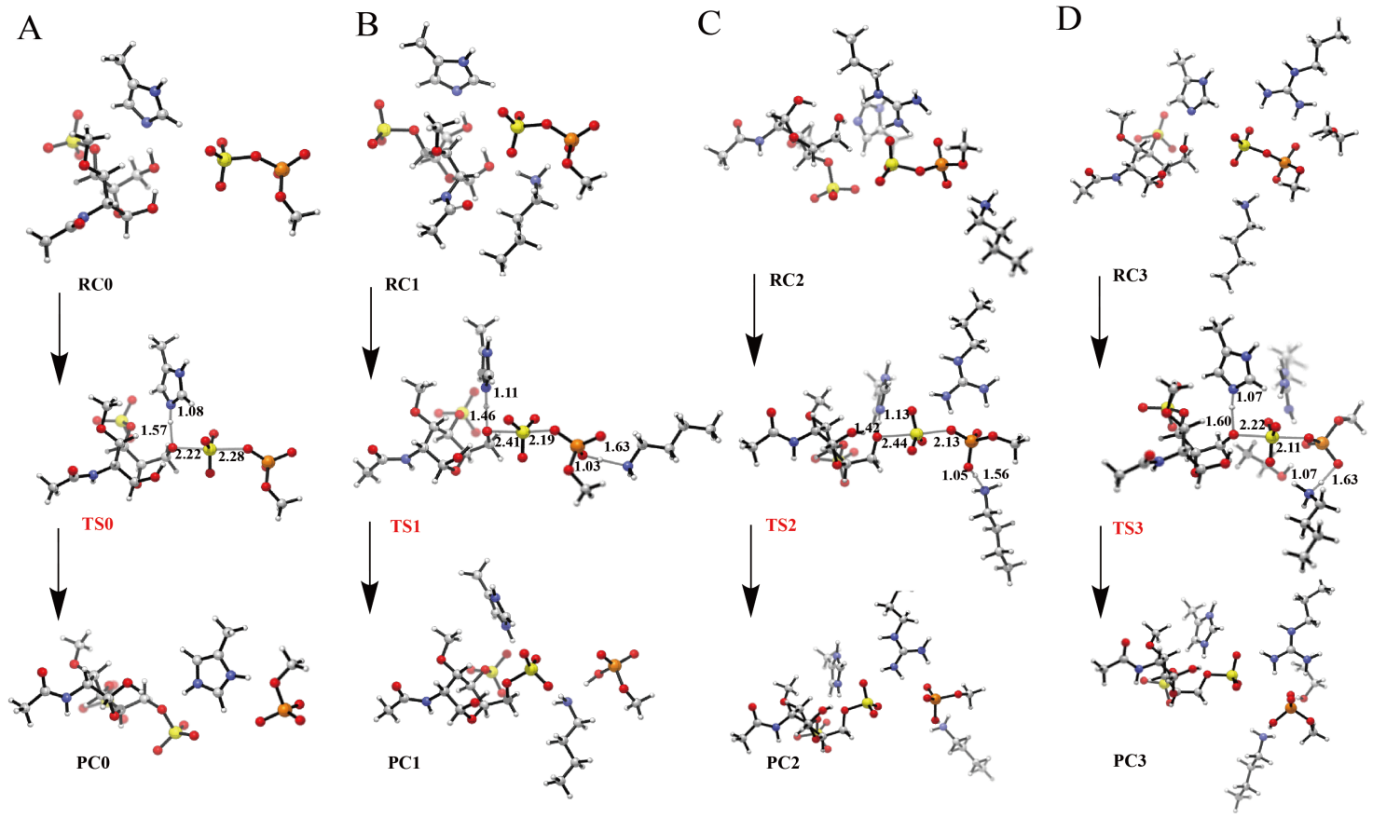


Fig. S8 Using GaussView to calculate reaction barriers for 12 different states. (A) Reaction models under three different states in the **model 1** (ground state model), including RC0, TS0, PC0 states; (B) Reaction models under three different states in the **model 2** (ground state + LYS model), including RC1, TS1, PC1 states; (C) Reaction models under three different states in the **model 3** (ground state + LYS + ARG model), including RC2, TS2, PC2 states; (D) Reaction models under three different states in the **model 4 (**ground state + LYS + ARG + THR model), including RC3, TS3, PC3 states.


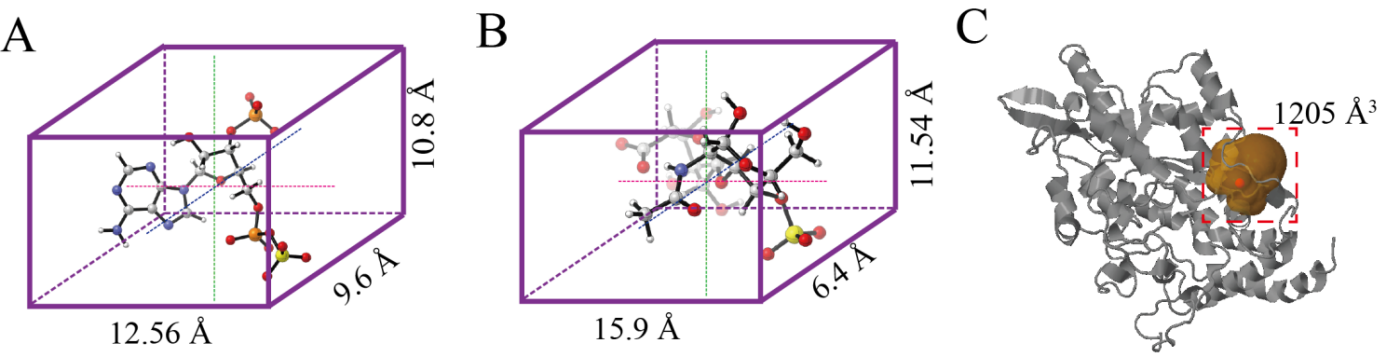


Fig. S9 Enzyme protein *Ec*CHST15 and small molecule volumes. (A) Molecular size of PAPS; (B) Molecular size of CSA; (C) Binding cavity volume of *Ec*CHST15 with PAPS.

**
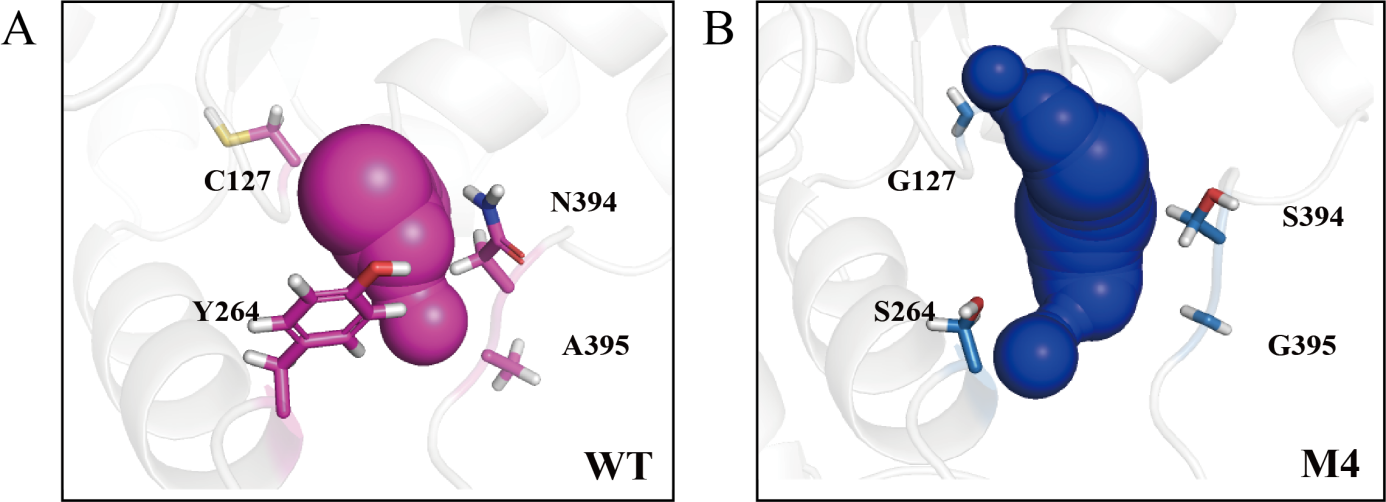
**

Fig. S10 Comparison of cofactor cavity volumes between WT and M4 mutants.(A) Active pocket structure analysis of the *Ec*CHST15(WT);(B) Active pocket structure analysis of the *Ec*CHST15(M4). The pocket volumes of the *Ec*CHST15 were measured using POVME 3.0.


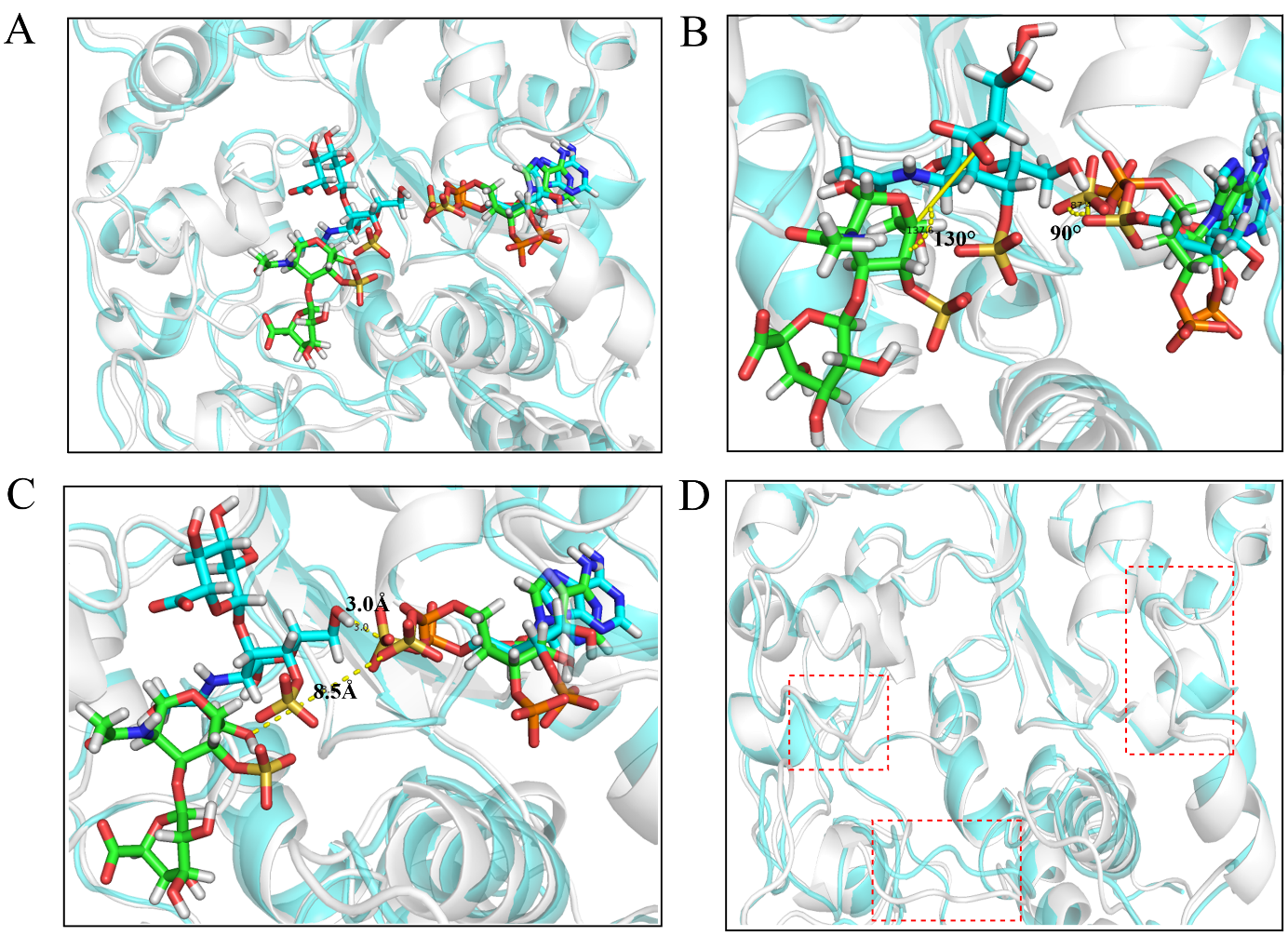


Fig. S11 Theoretical docking model of *Ec*CHST15 with [RC]/[TS3] states.(White and blue represent the enzyme spatial structures in [RC] and [TS3] states, respectively; Green and blue represent the small molecule structures in [RC] and [TS3] states, respectively). (A) MD results of *Ec*CHST15 with [RC]/[TS3] states; (B) Angle changes of *Ec*CHST15 with [RC]/[TS3] states; (C) Distance changes of *Ec*CHST15 with [RC]/[TS3] states; (D) Loop conformation changes of *Ec*CHST15 with [RC]/[TS3] states.

**
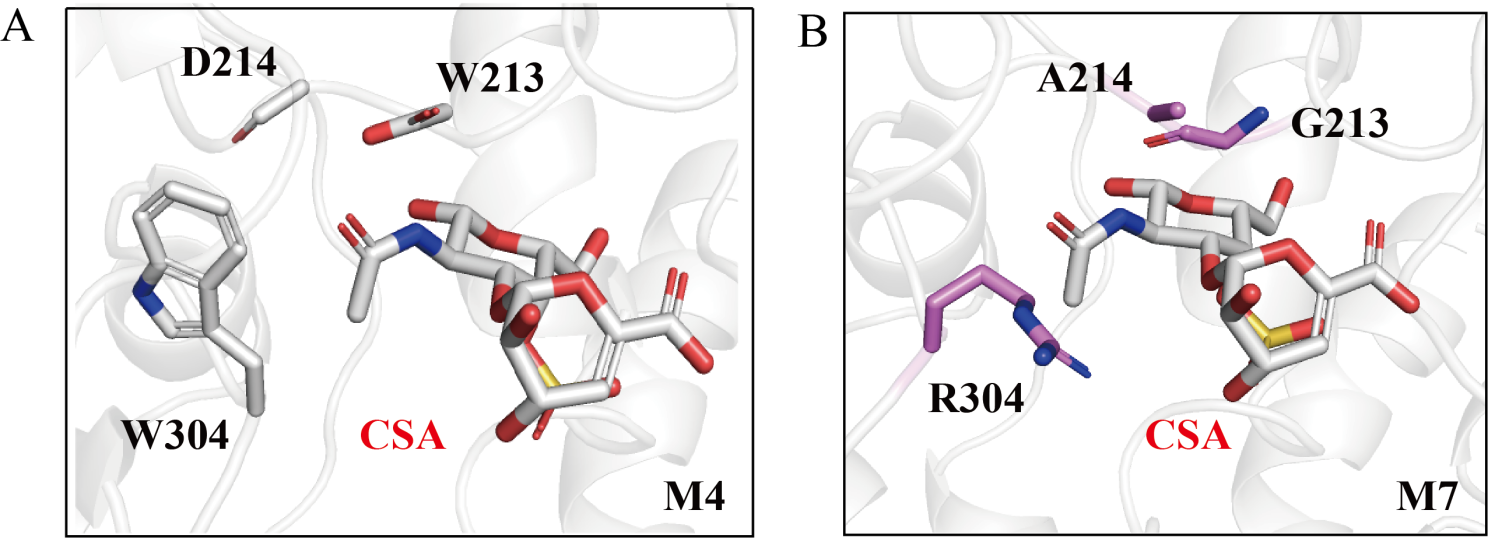
**

Fig. S12 Comparative analysis of M4 and M7 mutations**.**(A) The binding cavity of the M4 mutant;(B) The binding cavity of the M7 mutant.

Fig. S13 Molecular weight identification of polysaccharides.High-performance liquid chromatography-exclusion chromatography (HPLC-SEC) was employed, using an Ultrahyfrogel column (300 mm × 7.8 mm i.d., Waters Corporation, Milford, MA, USA) combined with a refractive index detector to measure the molecular weight of chondroitin. The mobile phase consisted of 0.1 M NaNO_3_ and the flow rate was maintained at 0.9 mL min^−1^.It was identified that the retention time of CSA was 16.74 min, Mn was 15457 Da, MW was 105028 Da, MP was 72992 and peak area was 813134.


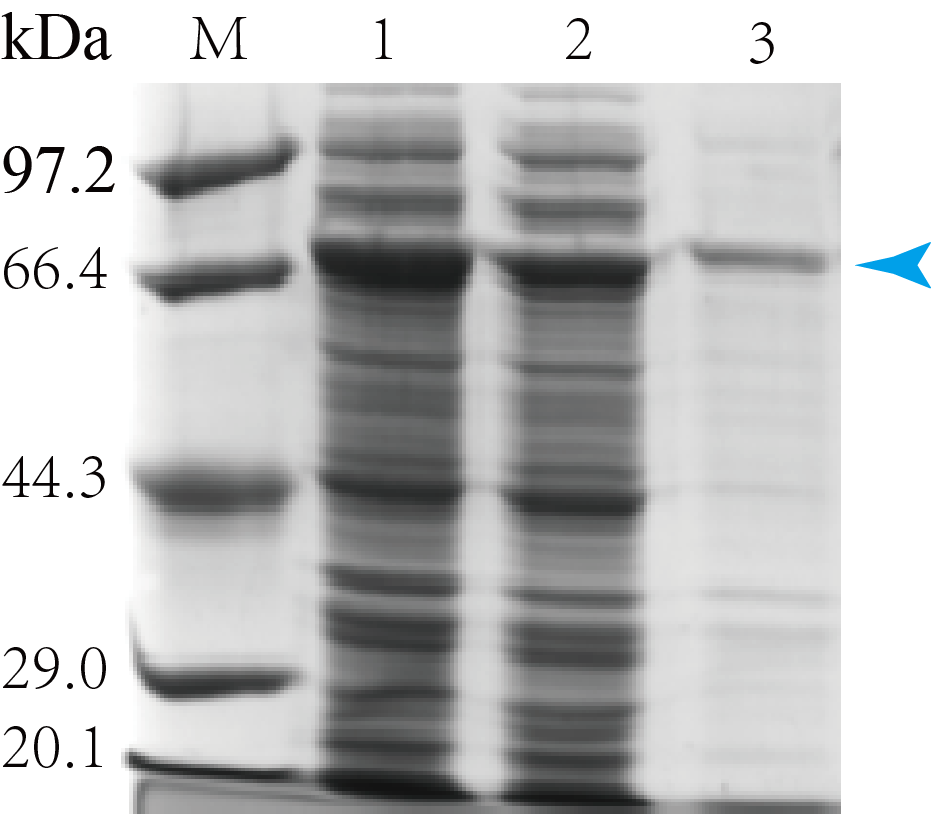


Fig. S14 Expression of pET22b-INPN-*Ec*CHST15^M7^ protein. M, marker; Lane 1, whole cell lysates from Rosetta(DE3) cells expressing pET22b-INPN-*Ec*CHST15M7; Lane 2, supernatant of Rosetta(DE3) cell lysates expressing pET22b-INPN-*Ec*CHST15M7; Lane 3, precipitation of Rosetta(DE3) cell lysates expressing pET22b-INPN-*Ec*CHST15M7;

**
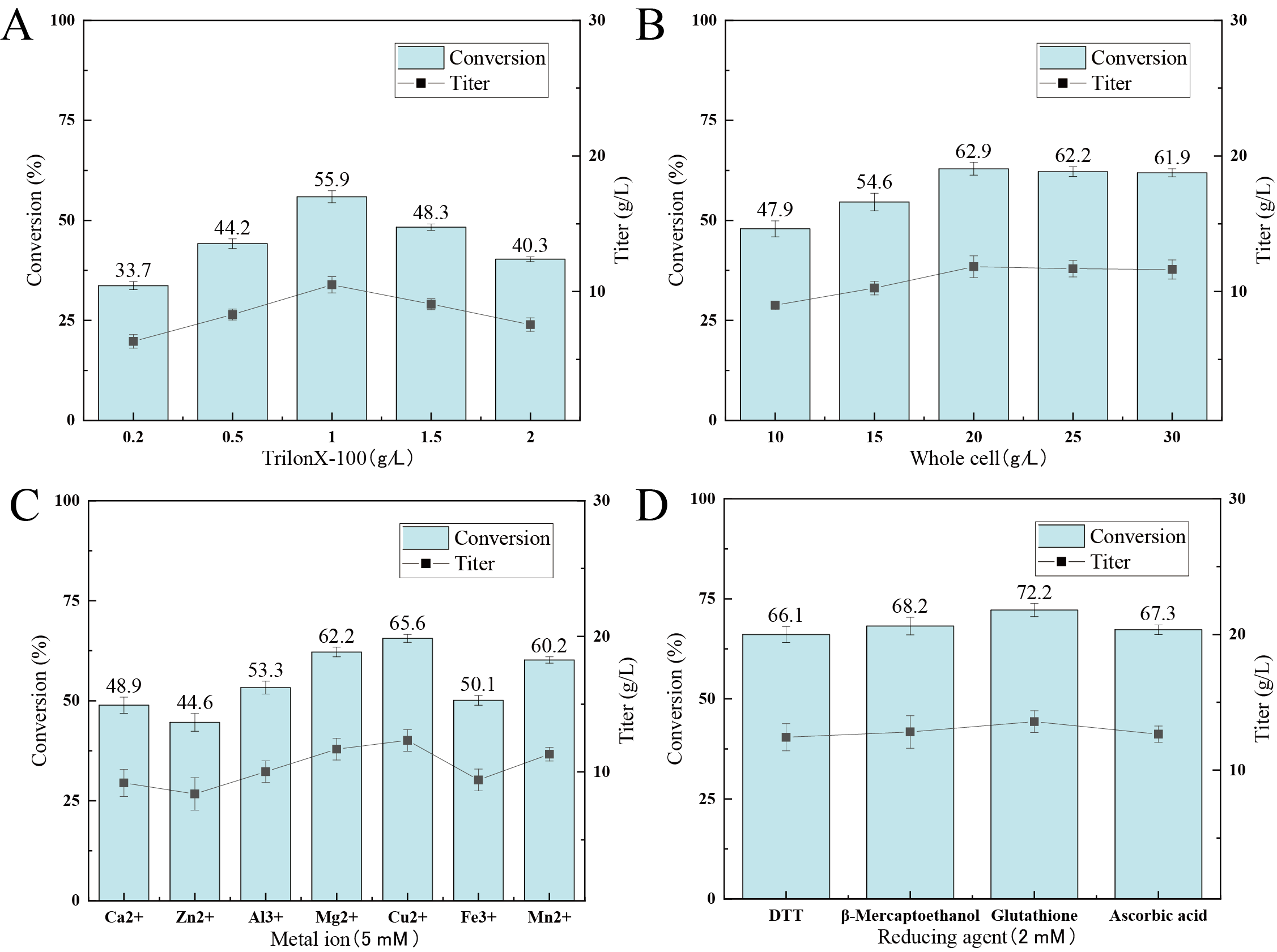
**

Fig. S15 Optimization of whole cell response system. (A) Concentration optimization of TritonX-100. Basic conditions include:pH 7.5, temperature 37°C, 15 g/L whole cells, ,TritonX-100, 60 mM ATP, 11 mM polyP_6_, 200 mM Na_2_SO_4_ and 120 mM pNPS, reaction duration 24 h;(B) Concentration optimization of Whole cells. Basic conditions include:pH 7.5, temperature 37°C, whole cells, 1 g/L TritonX-100, 60 mM ATP, 11 mM polyP_6_, 200 mM Na_2_SO_4_ and 120 mM pNPS, reaction duration 24 h;(C) Concentration optimization of Metal ions. Basic conditions include:pH 7.5, temperature 37°C,20 g/L whole cells, 1 g/L TritonX-100, 60 mM ATP, 11 mM polyP_6_, 200 mM Na_2_SO_4_ and 120 mM pNPS, reaction duration 24 h;(D) Concentration optimization of Reductant type. Basic conditions include:pH 7.5, temperature 37°C,20 g/L whole cells, 1 g/L TritonX-100,2 mM reducing agent, 60 mM ATP, 11 mM polyP_6_, 200 mM Na_2_SO_4_ and 120 mM pNPS, reaction duration 24 h.

Fig. S16 Mutation verification of key residues. The data represent mean ± SD, as determined from three independent experiments.

***
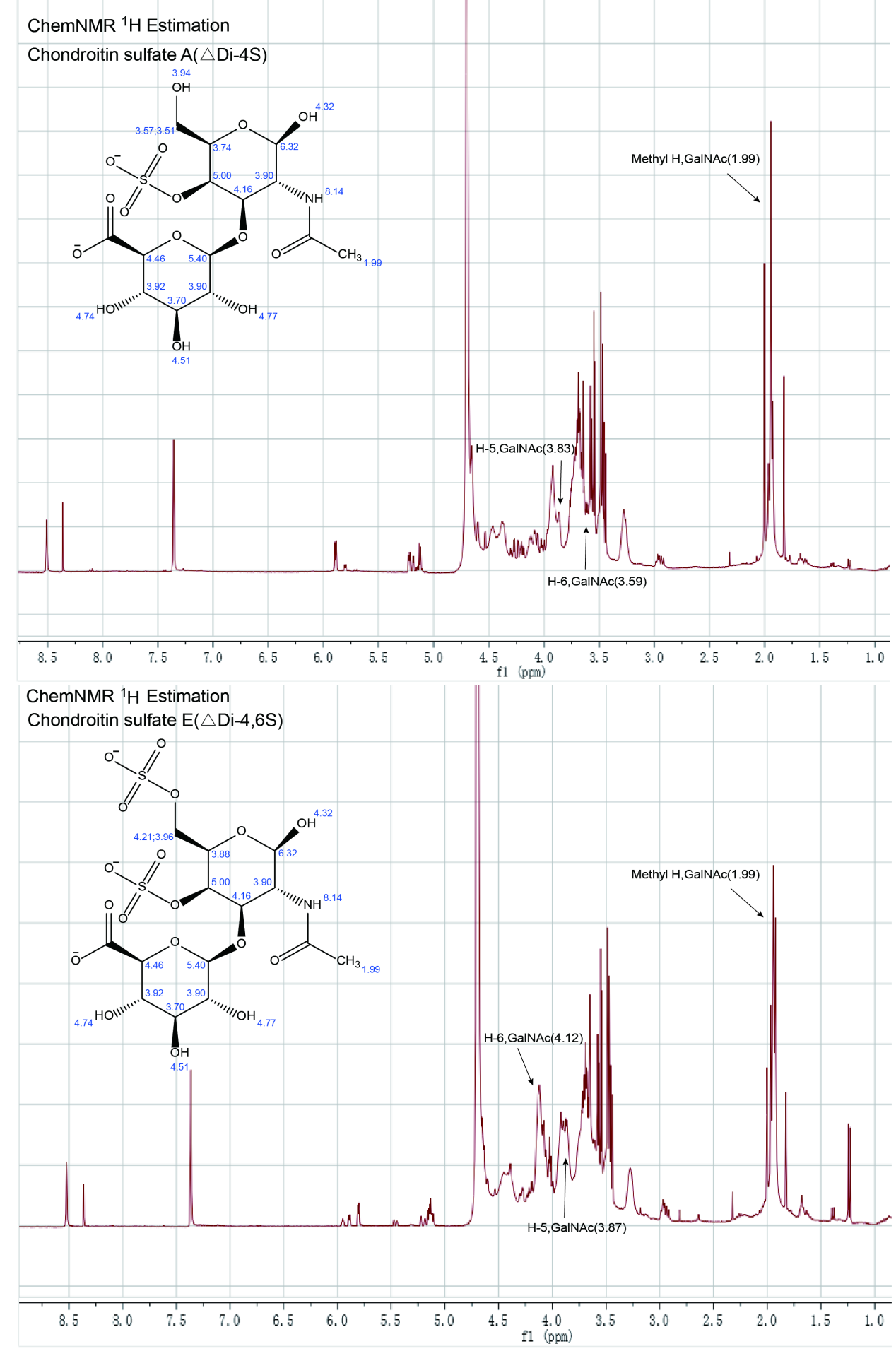
***

Fig. S17 Hydrogen nuclear magnetic resonance spectrometry (NMR-H) of CSA and CSE.


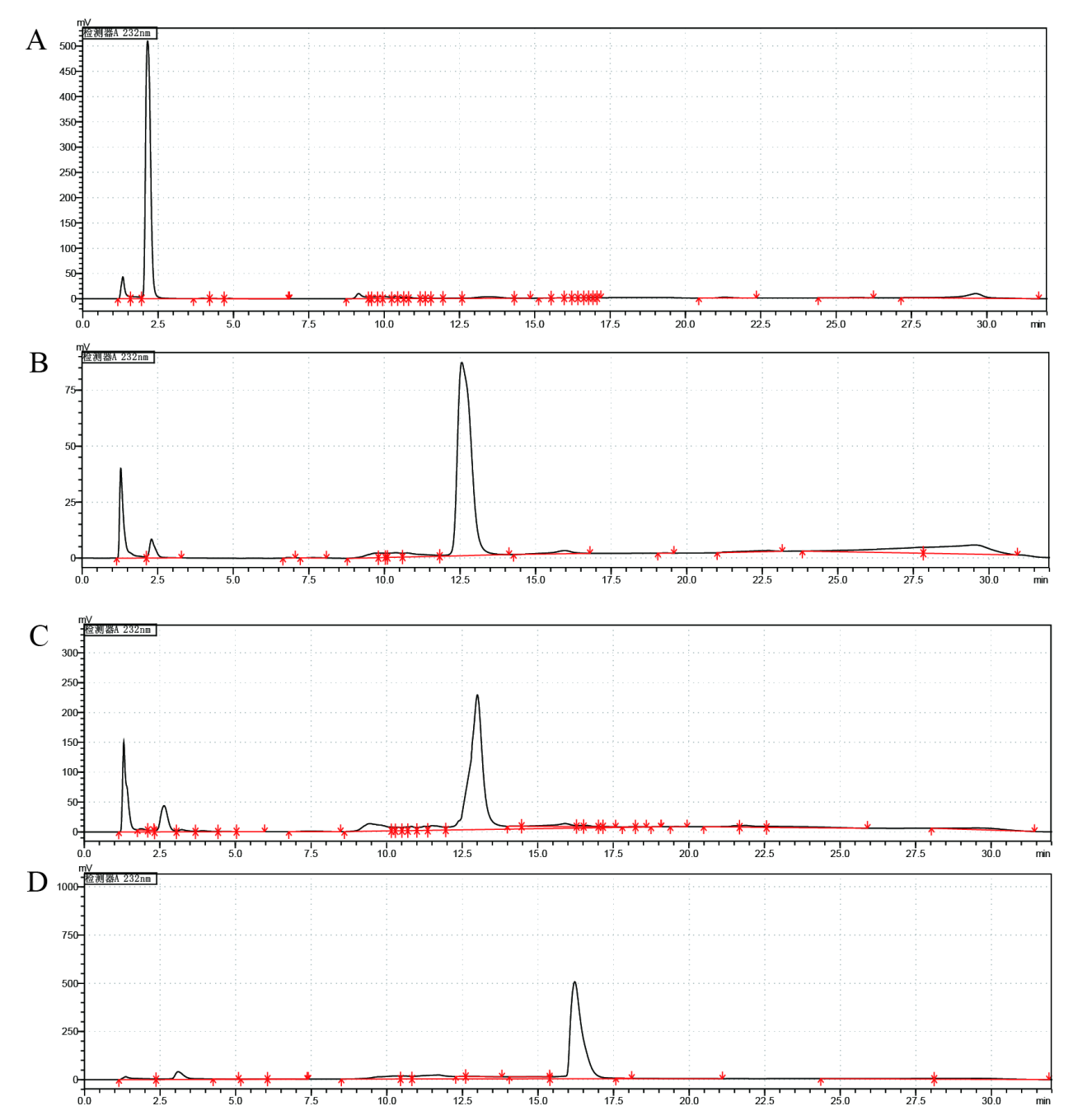


Fig. S18 HPLC diagram of chondroitin and chondroitin sulfate. (A)The peak elution time of chondroitin was 2.5min by HPLC; (B)The peak elution time of CSC was 12.0 min by HPLC; (C)The peak elution time of CSA was 12.5 min by HPLC; (D)The peak elution time of CSE was 15.8 min by HPLC.


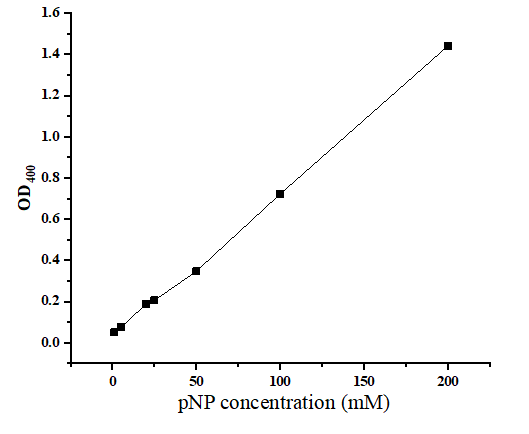


Fig. S19 The pNP standard curve. We prepared standard samples of PNP with concentrations of 1, 5, 20, 25, 50, 100, and 200 mM (dissolved in 20 mM Tris-HCl buffer solution, pH 7.0), measured the absorbance of the test samples at OD400nm, and plotted the standard curve. The data represent mean ± SD, as determined from three independent experiments.

Fig. S20 Time course of reactivity of chondroitinase ABC-I to CSE. Take 300 μL of nCSE solution (10 mg/ml) and mix it with 50 μL of chondroitinase ABC I pure enzyme at a concentration of 20 mg/ml. Incubate at pH 7.0 and 37°C for 12 hours. The peak area of CSE disaccharides in the digested products of CSE at the indicated times was determined using the HPLC method. As the digestion time increases, the peak area of CSE disaccharides increases, and after 12 hours of digestion, the peak area of CSE disaccharides remains essentially unchanged. Even if chondroitinase ABC I is added again to the digestion system, the peak area of CSE in the HPLC will not change.

# Calculated coordinates

RC0

*G*(Water) =-3055.13997 Hartree

------------------------------------

C 1.764057 5.178390 -2.152675

C 1.057933 4.009239 -1.548290

C 1.368516 3.190888 -0.488129

N -0.141308 3.513728 -2.036463

C -0.507289 2.444680 -1.278889

N 0.391005 2.222703 -0.331492

H 2.034688 4.990345 -3.199526

H 2.684395 5.380477 -1.596934

H -0.660703 3.883327 -2.822861

H 2.244708 3.214598 0.144150

H -1.413261 1.874210 -1.441940

P -6.707123 -0.052423 0.249186

S -3.746789 0.308354 -0.206917

O -6.474263 0.068303 1.736306

O -7.870028 0.661491 -0.403620

O -5.371761 0.423547 -0.640232

O -6.701366 -1.641589 -0.202021

O -3.096620 0.441254 -1.536867

O -3.559729 -1.017612 0.428578

O -3.503827 1.461602 0.694990

C 2.447003 -0.856753 -0.619540

N 3.365366 -3.166257 -0.631032

O 1.857294 -2.382258 1.766104

S 4.504420 1.563750 1.626651

C 2.146146 -2.366607 -0.654179

O 3.198356 0.938382 0.783935

C 1.253788 -2.782202 0.534873

O 4.321661 1.123940 3.035515

C 2.229396 -0.993938 1.939539

O 4.305328 3.014736 1.398958

C 3.063116 -0.492309 0.734561

O 5.711767 0.988980 0.976545

C 1.055209 -0.090855 2.337360

O 0.064799 0.104404 1.328936

C 3.818464 -3.900973 -1.679187

O -0.074749 -2.362099 0.388467

C 5.100275 -4.676980 -1.439839

O 3.238996 -3.945262 -2.774177

C 3.131569 0.713089 -2.311169

O 3.335870 -0.558780 -1.689190

H 1.507591 -0.309608 -0.753186

H 1.626967 -2.602818 -1.583777

H 4.060976 -0.943311 0.774703

H 1.231549 -3.873577 0.589604

H 3.866868 -3.226238 0.246164

H 2.889878 -1.009863 2.810850

H 0.557864 -0.558122 3.194554

H 1.465749 0.871898 2.658626

H 5.475307 -4.602036 -0.415069

H 4.925136 -5.730088 -1.681316

H 5.868027 -4.301615 -2.124904

H 3.339530 1.534134 -1.619971

H 1.146054 6.084841 -2.127376

H 3.820506 0.752573 -3.159108

H 2.099677 0.809569 -2.677571

H -0.138715 -1.392049 0.601493

C -7.525547 -2.553167 0.538733

H -7.231358 -2.575634 1.594105

H -7.375534 -3.541081 0.096472

H -8.586851 -2.283421 0.461915

H 0.270641 0.907614 0.753452

------------------------------------

PC0

*G*(Water) =-3055.123276 Hartree

------------------------------------

C 4.409914 2.570179 -2.836527

C 3.746262 1.668380 -1.850085

C 2.431981 1.319353 -1.681166

N 4.450725 0.987852 -0.868780

C 3.611958 0.256368 -0.132450

N 2.378979 0.444064 -0.610447

H 4.938727 3.386617 -2.331248

H 3.664379 3.005482 -3.506894

H 5.485024 1.028015 -0.698787

H 1.546922 1.615838 -2.222004

H 3.904964 -0.368356 0.695995

P 7.498532 -0.247220 0.697264

S 0.600334 -2.117654 1.336284

O 8.624857 0.270011 1.613188

O 7.924617 -1.374677 -0.263479

O 6.127746 -0.441487 1.374566

O 7.114348 1.096818 -0.341284

O 1.938893 -1.641426 1.758349

O 0.607058 -3.180780 0.301976

O -0.365353 -2.347668 2.436117

C -3.733196 1.102866 -0.748405

N -4.632668 2.145672 1.325332

O -2.230241 0.488586 1.625935

S -4.325385 -2.763563 -1.061098

C -3.525070 2.131364 0.382334

O -3.713595 -1.215216 -1.315680

C -2.229419 1.820716 1.144082

O -3.341162 -3.435368 -0.173356

C -2.400592 -0.568600 0.661660

O -4.358249 -3.275997 -2.450467

C -3.670738 -0.323370 -0.186726

O -5.657699 -2.561354 -0.434473

C -1.168985 -0.809264 -0.227179

O 0.059077 -0.724112 0.527703

C -5.516955 3.171500 1.443482

O -1.146351 2.090571 0.276976

C -6.610500 2.986841 2.478059

O -5.452904 4.197907 0.752991

C -5.016500 1.202525 -2.778508

O -4.991182 1.355206 -1.357068

H -2.926287 1.242227 -1.478474

H -3.462364 3.128953 -0.054591

H -4.561513 -0.485837 0.429730

H -2.168303 2.432061 2.050936

H -4.726158 1.356261 1.952129

H -2.544705 -1.454619 1.278486

H -1.254046 -1.792231 -0.694405

H -1.067339 -0.053975 -1.006007

H -6.492151 2.085551 3.086267

H -6.627674 3.863163 3.133101

H -7.574734 2.938310 1.960444

H -4.792235 0.171499 -3.071550

H 5.140671 2.022275 -3.442585

H -6.028337 1.465314 -3.097330

H -4.299678 1.884785 -3.257908

H -0.329626 1.825634 0.734325

C 8.063414 1.503451 -1.322877

H 8.990163 1.853840 -0.848875

H 7.623455 2.329942 -1.891749

H 8.302033 0.679509 -2.007453

H 1.534093 0.002430 -0.226328

------------------------------------

TS0

*G*(Water) =-3055.090384 Hartree

------------------------------------

C 1.165660 5.577678 -1.605521

C 0.605083 4.198913 -1.506558

C 0.796729 3.204992 -0.583543

N -0.282485 3.672778 -2.437958

C -0.613856 2.418758 -2.091054

N 0.035868 2.118259 -0.972198

H 1.747081 5.706219 -2.526013

H 1.824731 5.767695 -0.754605

H -0.638166 4.156657 -3.254195

H 1.416328 3.181873 0.299936

H -1.304881 1.768980 -2.604204

P -6.111316 -0.280297 0.186254

S -2.619741 -0.110616 -0.159567

O -5.974583 -0.120929 1.709663

O -7.386740 0.340461 -0.417244

O -4.837161 0.101148 -0.637545

O -6.211920 -1.948698 -0.119701

O -2.385892 -0.097816 -1.612918

O -2.816402 -1.383276 0.546702

O -2.643706 1.156163 0.587692

C 2.595286 -0.676243 -0.688231

N 3.823294 -2.821847 -0.427907

O 1.370429 -2.555862 1.128394

S 3.124962 1.722499 2.308715

C 2.607822 -2.194906 -0.934246

O 2.361113 1.017371 0.995185

C 1.382969 -2.860982 -0.270136

O 2.559961 1.058413 3.511858

C 1.371326 -1.161072 1.523115

O 2.712016 3.136044 2.125320

C 2.521811 -0.402949 0.816842

O 4.576325 1.468897 2.114784

C -0.005077 -0.489860 1.405805

O -0.418071 -0.257703 0.079783

C 4.796135 -3.352297 -1.211587

O 0.187974 -2.563067 -0.927816

C 5.964510 -3.987649 -0.480541

O 4.755649 -3.327064 -2.450753

C 3.604447 1.174547 -1.845735

O 3.773240 -0.118071 -1.260287

H 1.707504 -0.260282 -1.175335

H 2.574238 -2.380778 -2.008441

H 3.479908 -0.706171 1.253471

H 1.514069 -3.946456 -0.304542

H 3.902221 -2.935381 0.574986

H 1.603821 -1.194296 2.591122

H -0.723873 -1.150302 1.914858

H 0.043267 0.452546 1.970020

H 5.853769 -3.990388 0.607712

H 6.081305 -5.017970 -0.831272

H 6.877621 -3.441935 -0.741262

H 3.353074 1.926554 -1.091592

H 0.370185 6.332042 -1.600583

H 4.558348 1.426928 -2.316320

H 2.817397 1.158103 -2.613719

H -0.116468 -1.635745 -0.652224

C -7.248765 -2.675836 0.529794

H -7.125989 -2.661361 1.621851

H -7.193986 -3.713154 0.181342

H -8.241274 -2.272425 0.282334

H -0.073087 1.172396 -0.472429

------------------------------------

RC1

*G*(Water) =-3269.368286 Hartree

------------------------------------

C 4.671042 -4.542296 1.483507

C 3.642031 -3.880329 0.626627

C 3.690362 -2.772869 -0.186100

N 2.336165 -4.335904 0.523182

C 1.658912 -3.514879 -0.322181

N 2.452587 -2.555344 -0.769591

H 4.369147 -4.559031 2.538507

H 5.616764 -3.997275 1.411000

H 1.945483 -5.136082 1.004527

H 4.518086 -2.097413 -0.345969

H 0.608211 -3.620178 -0.549621

P -4.728452 -2.383953 -0.105510

S -1.787681 -2.030736 0.391943

O -4.485559 -1.780235 -1.481378

O -5.619478 -3.592707 0.021540

O -3.295383 -2.772431 0.634737

O -5.197841 -1.195733 0.931493

O -1.022657 -2.594039 1.525799

O -2.031638 -0.571375 0.476383

O -1.341898 -2.495695 -0.942823

C 1.420296 0.828415 0.331039

N 0.433187 3.086312 0.681660

O 1.368495 2.432632 -2.078160

S 5.295930 0.949896 0.111194

C 0.265754 1.800490 0.016040

O 3.742468 0.364761 -0.119876

C 0.129964 2.040611 -1.503140

O 5.608925 1.748362 -1.104429

C 2.533231 1.605921 -1.843434

O 6.054575 -0.317361 0.232784

C 2.704279 1.336511 -0.331296

O 5.236634 1.762226 1.354977

C 2.592765 0.363841 -2.740989

O 1.603257 -0.631834 -2.476845

C -0.318572 3.499701 1.735549

O -0.478053 0.954923 -2.165319

C 0.051354 4.849365 2.321683

O -1.245574 2.827423 2.206745

C 1.862657 -0.566866 2.231739

O 1.570960 0.748991 1.741827

H 1.160737 -0.155501 -0.070134

H -0.658524 1.359340 0.388193

H 2.985852 2.271072 0.167212

H -0.523819 2.898933 -1.669459

H 1.153420 3.708249 0.337078

H 3.367985 2.236353 -2.160858

H 2.454236 0.697832 -3.775239

H 3.594696 -0.068723 -2.649177

H 0.735200 5.427337 1.693216

H -0.862232 5.426877 2.488221

H 0.525883 4.686712 3.296191

H 2.801878 -0.944234 1.815214

H 4.852191 -5.579404 1.173723

H 1.948893 -0.473955 3.317943

H 1.048823 -1.261293 1.987406

H 0.168481 0.197823 -2.217070

C -6.502447 -0.623415 0.742844

H -6.626258 -0.251521 -0.281061

H -6.580408 0.212898 1.440900

H -7.285386 -1.358442 0.961658

H 1.952372 -1.320621 -1.823453

C -3.637312 5.235256 0.369609

H -3.694643 5.729833 -0.608513

H -2.584546 5.220897 0.675954

C -4.203610 3.813022 0.300982

H -4.164384 3.353363 1.297563

H -5.262654 3.849525 0.009529

C -3.432616 2.928018 -0.686695

H -3.454848 3.382127 -1.687741

H -2.384905 2.881311 -0.370536

C -4.007809 1.518335 -0.744775

H -3.895579 0.993731 0.204380

H -5.068782 1.535616 -1.008667

N -3.326939 0.664144 -1.771148

H -2.300171 0.630986 -1.644734

H -3.702943 -0.320756 -1.703220

H -3.498531 1.016625 -2.715878

H -4.189489 5.849029 1.090970

------------------------------------

PC1

*G*(Water) =-3269.361223 Hartree

------------------------------------

C 3.060210 6.145181 -0.015460

C 2.184583 4.954385 -0.216188

C 1.609570 4.085313 0.672138

N 1.791298 4.508897 -1.473858

C 1.013732 3.422875 -1.360793

N 0.897468 3.158825 -0.062652

H 4.026033 6.011916 -0.516016

H 3.242555 6.292879 1.051705

H 2.048185 4.937483 -2.356019

H 1.652575 4.030414 1.747130

H 0.574623 2.847246 -2.163850

P -5.899141 1.548943 -0.229783

S -0.900629 0.309588 -1.732070

O -5.117847 1.168573 1.149629

O -7.000166 2.529254 0.134660

O -4.926102 1.875811 -1.347494

O -6.570621 0.092864 -0.680229

O -0.331468 0.959932 -2.932524

O -2.018625 -0.633260 -1.977668

O -1.138963 1.221337 -0.583715

C 3.359153 -0.378759 -0.431645

N 4.943028 -2.271310 -0.163799

O 2.121600 -2.991997 -0.211035

S 1.318224 0.674222 2.733436

C 3.965567 -1.652256 -1.049780

O 1.752868 0.450874 1.128944

C 2.876929 -2.691044 -1.382497

O 0.500440 -0.511194 3.097549

C 1.500241 -1.911419 0.513466

O 0.556991 1.941952 2.613720

C 2.485522 -0.736931 0.778840

O 2.595449 0.778453 3.481813

C 0.166766 -1.467814 -0.074336

O 0.389228 -0.652738 -1.248707

C 6.278998 -2.306198 -0.408462

O 2.094345 -2.343525 -2.496940

C 7.127681 -2.991483 0.645637

O 6.791883 -1.810548 -1.421725

C 4.168653 1.862435 -0.003908

O 4.446023 0.462429 -0.074942

H 2.732464 0.116101 -1.183967

H 4.491436 -1.381610 -1.966050

H 3.153636 -1.005646 1.604865

H 3.360303 -3.635318 -1.640838

H 4.594927 -2.735406 0.665680

H 1.245333 -2.359935 1.476444

H -0.429895 -2.343197 -0.347214

H -0.358161 -0.877058 0.677328

H 6.544764 -3.452887 1.447931

H 7.741445 -3.757359 0.161472

H 7.804587 -2.249850 1.083368

H 3.581239 2.110635 0.883133

H 2.592268 7.051905 -0.415605

H 5.138606 2.364161 0.042615

H 3.631011 2.202519 -0.899821

H 1.488710 -1.604974 -2.268812

C -7.528221 -0.520915 0.189624

H -7.083383 -0.742980 1.167551

H -7.836800 -1.456954 -0.284048

H -8.404402 0.123712 0.328655

H 0.375051 2.362678 0.306464

C -3.619423 -5.984748 0.223491

H -2.851606 -6.159811 0.988387

H -3.116036 -5.949708 -0.751321

C -4.379067 -4.680846 0.496597

H -5.162056 -4.546688 -0.263928

H -4.898235 -4.755437 1.463292

C -3.472672 -3.441934 0.507627

H -2.687666 -3.564182 1.269186

H -2.958804 -3.348669 -0.460384

C -4.252000 -2.151612 0.788425

H -5.026217 -2.006970 0.026352

H -4.769764 -2.231891 1.752852

N -3.440215 -0.919751 0.815871

H -2.952124 -0.804878 -0.074681

H -4.441155 0.410169 1.018516

H -2.721110 -0.984482 1.536253

H -4.295417 -6.848763 0.221975

------------------------------------

TS1

*G*(Water) =-3269.324867 Hartree

------------------------------------

C -2.181085 5.255195 2.466196

C -1.729270 3.850675 2.245121

C -1.804630 3.031274 1.149105

N -1.110647 3.091195 3.230331

C -0.826126 1.869739 2.745799

N -1.242863 1.813943 1.486670

H -2.928985 5.311435 3.266000

H -2.630734 5.645288 1.549397

H -0.898947 3.402471 4.171103

H -2.224957 3.213174 0.171851

H -0.324591 1.074384 3.274008

P 4.708586 -1.319026 0.994746

S 1.394473 -0.632382 0.873506

O 4.648000 -0.936515 -0.568935

O 6.021966 -0.903944 1.618437

O 3.417956 -0.823957 1.677823

O 4.562371 -2.961632 1.016741

O 0.853364 -0.738587 2.232774

O 1.475556 -1.822882 0.025206

O 1.668298 0.689557 0.303930

C -4.038274 -0.457257 0.307181

N -5.411410 -2.375705 -0.475360

O -2.683638 -2.261940 -1.491703

S -3.649848 2.328916 -2.363050

C -4.264342 -1.978603 0.333661

O -3.282530 1.372769 -1.041170

C -3.008657 -2.721535 -0.174169

O -2.911867 1.740535 -3.511064

C -2.449418 -0.842748 -1.679628

O -3.129688 3.637185 -1.893389

C -3.630982 -0.023820 -1.104097

O -5.127532 2.264039 -2.510672

C -1.064175 -0.382791 -1.198686

O -0.905806 -0.380230 0.196219

C -6.579056 -2.850044 0.027607

O -1.946668 -2.661333 0.727857

C -7.635330 -3.232428 -0.992954

O -6.794585 -2.967544 1.243304

C -5.052421 1.365398 1.504164

O -5.240224 0.185209 0.720856

H -3.227463 -0.221727 1.003509

H -4.470574 -2.289496 1.358532

H -4.507512 -0.139243 -1.751803

H -3.262001 -3.776434 -0.317036

H -5.291622 -2.366172 -1.480607

H -2.458005 -0.720762 -2.766373

H -0.328257 -1.060614 -1.661947

H -0.897204 0.621308 -1.616589

H -7.297947 -3.145667 -2.029810

H -7.951202 -4.263220 -0.803904

H -8.507911 -2.585789 -0.850804

H -4.568756 2.158054 0.925326

H -1.342968 5.905873 2.742242

H -6.048446 1.689973 1.816942

H -4.446409 1.152237 2.396909

H -1.490514 -1.750780 0.641965

C 5.600441 -3.741412 0.405411

H 5.667869 -3.527696 -0.668909

H 5.332175 -4.791083 0.546249

H 6.570028 -3.544788 0.877533

H -1.133857 0.906731 0.857576

C 11.283749 2.093521 -1.415937

H 11.342855 2.204423 -2.506483

H 11.901789 1.230969 -1.134691

C 9.831971 1.899728 -0.961852

H 9.796810 1.821703 0.134377

H 9.242068 2.788770 -1.227973

C 9.176965 0.656316 -1.577645

H 9.204554 0.731410 -2.675116

H 9.760636 -0.237096 -1.308811

C 7.727669 0.462681 -1.121619

H 7.673719 0.362655 -0.031955

H 7.126679 1.339145 -1.394159

N 7.052962 -0.724145 -1.691491

H 7.590704 -1.565366 -1.479366

H 5.581064 -0.867405 -1.010292

H 7.017977 -0.659694 -2.709282

H 11.728861 2.987669 -0.962799

------------------------------------

RC2

*G*(Water) =-3593.141335 Hartree

------------------------------------

C 0.764556 0.375740 5.536774

C 1.332105 0.865725 4.247156

C 1.528067 0.252698 3.031968

N 1.787880 2.162017 4.069576

C 2.226806 2.292748 2.792388

N 2.084383 1.151260 2.136062

H 1.492274 0.453583 6.354158

H 0.476389 -0.674574 5.437423

H 1.793452 2.890581 4.773064

H 1.311255 -0.765969 2.742662

H 2.620878 3.214706 2.388774

P -4.512087 0.606208 0.387231

S -2.204985 -0.179133 -1.348065

O -4.894093 -0.525547 1.325098

O -3.666746 1.751857 0.899202

O -3.868162 -0.025303 -1.004532

O -5.932877 1.135725 -0.220407

O -1.795461 1.159976 -1.824501

O -2.233460 -1.222991 -2.397203

O -1.585316 -0.600101 -0.068932

C 4.609351 -0.753069 0.106536

N 6.195440 -1.631364 -1.593483

O 3.684699 -0.513567 -2.624259

S 1.674554 -3.188963 0.782796

C 5.556167 -0.432618 -1.065238

O 2.477318 -1.717952 0.669326

C 4.799600 0.275357 -2.210225

O 0.995510 -3.373863 -0.526378

C 2.718114 -0.926734 -1.629629

O 0.771915 -2.915691 1.925812

C 3.419184 -1.570514 -0.407386

O 2.730571 -4.199607 1.051084

C 1.699090 0.159435 -1.278562

O 2.234181 1.250998 -0.513759

C 7.516619 -1.916234 -1.456515

O 4.474495 1.607042 -1.907286

C 7.984515 -3.210041 -2.095894

O 8.305541 -1.181543 -0.845223

C 4.984106 -1.162939 2.442867

O 5.339733 -1.471243 1.091703

H 4.254012 0.191499 0.530993

H 6.351725 0.222792 -0.708696

H 3.797973 -2.559085 -0.689072

H 5.446932 0.309314 -3.089616

H 5.628404 -2.254626 -2.154297

H 2.145677 -1.711531 -2.131489

H 1.315550 0.584799 -2.210348

H 0.864051 -0.297175 -0.740786

H 7.210104 -3.717601 -2.678229

H 8.838587 -2.993830 -2.745086

H 8.329652 -3.884763 -1.304985

H 3.941358 -1.420565 2.650195

H -0.125590 0.947115 5.827877

H 5.648619 -1.755210 3.077153

H 5.138932 -0.095049 2.653133

H 3.714250 1.620835 -1.272547

C -5.936450 2.237270 -1.150848

H -5.509624 3.131991 -0.686572

H -6.980855 2.416596 -1.411775

H -5.368110 1.977116 -2.049950

H 2.241615 1.047666 0.479232

C -11.243765 -2.977679 -1.561045

H -11.774491 -2.024112 -1.676698

H -11.661104 -3.489868 -0.684867

C -9.735730 -2.752011 -1.398511

H -9.226511 -3.721716 -1.310321

H -9.334238 -2.268135 -2.299493

C -9.403520 -1.892666 -0.170389

H -9.884370 -0.909349 -0.266982

H -9.818900 -2.366882 0.729780

C -7.899252 -1.710253 0.002576

H -7.390135 -2.669299 0.127761

H -7.449546 -1.185532 -0.843744

N -7.573099 -0.892577 1.219192

H -7.969615 -1.304664 2.068277

H -6.522037 -0.796933 1.337583

H -7.941621 0.058579 1.132335

H -11.457839 -3.591335 -2.443810

C 2.496132 6.190585 -1.939074

H 2.050281 7.062069 -1.443737

H 2.121447 6.160687 -2.969912

C 2.161031 4.898608 -1.190242

H 2.645097 4.044796 -1.682324

H 2.556936 4.942501 -0.167322

C 0.651676 4.648255 -1.120240

H 0.154661 5.493970 -0.630684

H 0.236080 4.552216 -2.133718

N 0.395678 3.436880 -0.341125

H 1.120945 2.710738 -0.364543

C -0.776045 3.096424 0.196277

N -1.892603 3.828470 -0.022649

N -0.850046 2.072574 1.061975

H -1.938521 4.350456 -0.888715

H -2.760416 3.346289 0.225781

H -0.037010 1.481348 1.181285

H -1.758731 1.612160 1.138104

H 3.579990 6.344946 -1.980668

------------------------------------

PC2

*G*(Water) =-3593.137169 Hartree

------------------------------------

C 1.797957 1.669730 5.331520

C 1.675701 1.503944 3.854663

C 1.748056 0.393022 3.059032

N 1.463419 2.571142 2.987139

C 1.408067 2.136664 1.723040

N 1.580822 0.816332 1.753246

H 2.643421 2.318927 5.586054

H 1.959047 0.694820 5.797720

H 1.361900 3.542329 3.260600

H 1.916642 -0.648212 3.292608

H 1.248460 2.729798 0.837774

P -4.890175 0.446340 -1.424661

S -0.020229 0.415066 -1.414981

O -4.900488 -1.171507 -1.515529

O -4.610571 0.868575 0.023575

O -4.041306 1.001648 -2.541938

O -6.447293 0.881458 -1.782386

O 0.228762 1.844397 -1.089773

O -0.216507 0.119004 -2.846758

O -0.945602 -0.270118 -0.488821

C 4.613829 -0.236190 -0.232904

N 6.467183 -0.971357 -1.718228

O 3.789792 -1.701297 -2.594633

S 3.065968 -3.127612 1.889650

C 5.313149 -0.091449 -1.597242

O 3.170523 -1.652632 1.066761

C 4.334020 -0.393191 -2.747150

O 2.438613 -4.065473 0.925426

C 3.145962 -2.050870 -1.352810

O 2.204502 -2.737062 3.030026

C 3.988546 -1.631534 -0.117157

O 4.463656 -3.471479 2.247038

C 1.675546 -1.649020 -1.297181

O 1.522980 -0.226074 -1.034280

C 7.755181 -0.537180 -1.754177

O 3.364394 0.610629 -2.915361

C 8.812606 -1.614487 -1.901686

O 8.064958 0.658645 -1.664493

C 5.113976 0.566406 1.990114

O 5.588557 -0.018126 0.774947

H 3.830038 0.528779 -0.167862

H 5.676505 0.931935 -1.698028

H 4.812632 -2.341448 0.009064

H 4.889556 -0.437734 -3.685942

H 6.290898 -1.958475 -1.857389

H 3.119435 -3.143163 -1.373530

H 1.217123 -1.884965 -2.261361

H 1.180113 -2.204504 -0.499332

H 8.401917 -2.612256 -2.080544

H 9.476951 -1.345306 -2.728430

H 9.413328 -1.640306 -0.985948

H 4.467908 -0.122025 2.541553

H 0.889915 2.111455 5.757347

H 6.001543 0.795976 2.584662

H 4.566129 1.497996 1.789611

H 2.698970 0.547844 -2.198373

C -7.496605 0.506028 -0.880366

H -7.632261 -0.582839 -0.869143

H -8.413777 0.974858 -1.245194

H -7.284024 0.855799 0.136297

H 1.612664 0.220650 0.923029

C -9.245155 -6.470777 1.071105

H -9.887367 -5.905836 1.759167

H -8.536338 -7.050344 1.676625

C -8.506384 -5.529750 0.111709

H -7.895992 -6.122007 -0.585250

H -9.237368 -4.985139 -0.503264

C -7.606176 -4.519912 0.837029

H -8.211639 -3.917301 1.530471

H -6.866524 -5.057355 1.448962

C -6.873332 -3.585006 -0.130907

H -6.254142 -4.169789 -0.822068

H -7.601115 -3.038363 -0.743420

N -5.992290 -2.582659 0.505458

H -5.277031 -3.048014 1.065066

H -5.284161 -1.643716 -0.692504

H -6.526108 -2.004982 1.155650

H -9.879279 -7.179923 0.525393

C -2.406791 8.567612 0.093323

H -2.908812 8.815646 1.036520

H -3.146441 8.640697 -0.713303

C -1.786405 7.167203 0.148046

H -1.274897 6.946538 -0.797943

H -1.029579 7.126216 0.942905

C -2.843722 6.091863 0.404633

H -3.347794 6.283249 1.361617

H -3.598787 6.121649 -0.390845

N -2.227069 4.761157 0.423368

H -1.216699 4.710938 0.465807

C -2.899613 3.602070 0.464133

N -4.232905 3.575521 0.560939

N -2.233175 2.431685 0.444106

H -4.780181 4.418230 0.651401

H -4.695167 2.667219 0.413395

H -1.311451 2.395332 0.010913

H -2.813762 1.599885 0.298321

H -1.635713 9.324361 -0.086846

------------------------------------

TS2

*G*(Water) =-3593.099006 Hartree

------------------------------------

C 1.867001 2.771555 4.565072

C 1.622521 2.378499 3.146541

C 1.663430 1.157956 2.523586

N 1.309653 3.290329 2.146318

C 1.170770 2.641042 0.975634

N 1.382759 1.348229 1.184754

H 2.715195 3.461694 4.645362

H 2.092414 1.881338 5.157897

H 1.200183 4.290127 2.269143

H 1.908982 0.185280 2.921649

H 0.927935 3.095038 0.027780

P -4.311420 -0.014855 -1.046138

S -1.057724 0.152227 -1.271426

O -4.256803 -1.602086 -1.186775

O -4.577732 0.413319 0.391265

O -3.086323 0.607719 -1.756431

O -5.540671 0.469262 -2.013288

O -0.662620 1.558272 -1.435610

O -0.920823 -0.781040 -2.388007

O -1.353128 -0.341330 0.077842

C 4.409584 0.055773 -0.469465

N 6.064375 -0.890216 -2.064306

O 3.398135 -2.052498 -2.159969

S 3.768416 -2.228629 2.595752

C 4.839499 -0.108526 -1.937223

O 3.466605 -1.078489 1.420009

C 3.720991 -0.790809 -2.755818

O 3.249960 -3.512104 2.053855

C 2.983667 -2.063985 -0.769905

O 2.984493 -1.668305 3.724548

C 4.011797 -1.306515 0.105846

O 5.240836 -2.221198 2.798870

C 1.526977 -1.622524 -0.563545

O 1.293467 -0.254287 -0.784268

C 7.266346 -0.380335 -2.434040

O 2.618766 0.040190 -2.961856

C 8.409206 -1.374777 -2.526395

O 7.444138 0.821561 -2.682780

C 5.104638 1.568414 1.263676

O 5.490769 0.627036 0.260182

H 3.544606 0.724842 -0.443705

H 5.040684 0.876144 -2.361133

H 4.924905 -1.905125 0.200905

H 4.121483 -1.056565 -3.738766

H 5.987523 -1.891496 -1.936258

H 3.028735 -3.121297 -0.494877

H 0.913884 -2.223179 -1.254615

H 1.245131 -1.907762 0.460386

H 8.112035 -2.407453 -2.321735

H 8.844768 -1.319472 -3.529167

H 9.184621 -1.082099 -1.810499

H 4.499679 1.096703 2.044055

H 0.989742 3.263095 5.001897

H 6.030745 1.955874 1.696407

H 4.538353 2.401791 0.822593

H 2.045187 0.045784 -2.117913

C -6.879162 0.095265 -1.636740

H -6.982998 -0.996050 -1.604039

H -7.542218 0.500327 -2.403693

H -7.139939 0.519223 -0.660974

H 1.358836 0.576767 0.361505

C -7.143401 -7.872666 1.118010

H -8.091406 -7.529398 1.552042

H -6.461690 -8.104625 1.946481

C -6.542848 -6.804504 0.195223

H -5.611794 -7.185362 -0.248654

H -7.229863 -6.614587 -0.641975

C -6.251947 -5.482072 0.918793

H -7.181642 -5.087742 1.355116

H -5.562247 -5.664487 1.756275

C -5.644661 -4.425884 -0.010045

H -4.710294 -4.799759 -0.445582

H -6.326114 -4.217729 -0.843502

N -5.337216 -3.129676 0.636257

H -4.698244 -3.267106 1.420286

H -4.697509 -2.152613 -0.403137

H -6.184762 -2.719445 1.029979

H -7.340495 -8.804169 0.573855

C -4.117740 8.311455 -0.552737

H -4.650106 8.615437 0.356787

H -4.865235 8.066095 -1.316993

C -3.193217 7.118075 -0.281011

H -2.657105 6.841423 -1.198002

H -2.436825 7.398108 0.464584

C -3.973275 5.902400 0.224952

H -4.500070 6.155179 1.155009

H -4.719650 5.606672 -0.522075

N -3.068542 4.768860 0.455744

H -2.079658 4.969548 0.545923

C -3.454313 3.506183 0.674699

N -4.742368 3.178850 0.800277

N -2.522383 2.533313 0.815131

H -5.471450 3.877052 0.816649

H -4.989518 2.183558 0.718573

H -1.681430 2.611358 0.250681

H -2.893982 1.582626 0.826713

H -3.540833 9.171571 -0.908981

------------------------------------

RC3

*G*(Water) =-3748.17766 Hartree

------------------------------------

C -0.971435 -6.353445 0.263636

C -0.809146 -4.914727 -0.112408

C -1.628046 -3.822037 0.052978

N 0.313054 -4.416725 -0.758000

C 0.140134 -3.081858 -0.956893

N -1.029163 -2.691075 -0.476366

H -0.955562 -7.006449 -0.617972

H -1.929782 -6.493455 0.771933

H 1.122979 -4.954775 -1.040255

H -2.590531 -3.767279 0.540026

H 0.873420 -2.441320 -1.428369

P 4.001218 2.369327 0.485820

S 1.805012 0.583613 -0.424920

O 3.054042 3.273512 1.249287

O 4.891314 1.396582 1.233531

O 3.160101 1.546970 -0.702209

O 4.973233 3.229068 -0.490952

O 1.554796 0.039866 -1.776709

O 0.764725 1.500683 0.090912

O 2.260208 -0.438714 0.561708

C -5.136823 -1.119375 -0.913211

N -7.054777 0.450834 -1.075332

O -4.513942 1.594205 -0.136323

S -4.349210 -2.181844 2.745480

C -5.747295 0.105336 -1.620265

O -4.112730 -1.884379 1.111571

C -4.819057 1.334101 -1.506729

O -3.763255 -1.011770 3.451874

C -3.947418 0.526750 0.664413

O -3.595097 -3.446717 2.905332

C -4.801584 -0.759202 0.536901

O -5.820825 -2.306124 2.919354

C -2.447743 0.323539 0.436487

O -2.134822 -0.249929 -0.830545

C -8.226693 0.295578 -1.743472

O -3.692393 1.244796 -2.337022

C -9.476273 0.724023 -0.997131

O -8.293949 -0.171482 -2.889743

C -5.511943 -3.484760 -1.138690

O -6.079294 -2.183253 -0.976883

H -4.216272 -1.401058 -1.435774

H -5.894082 -0.130304 -2.674940

H -5.748131 -0.617486 1.070247

H -5.370503 2.219818 -1.831260

H -7.079477 0.884039 -0.160894

H -4.047509 0.894130 1.689366

H -1.951966 1.298133 0.514282

H -2.068761 -0.317237 1.238868

H -9.270983 1.209442 -0.038710

H 10.048796 1.408166 -1.630932

H 10.095965 -0.161348 -0.817795

H -4.920589 -3.774272 -0.264358

H -0.174923 -6.688069 0.939972

H -6.351888 -4.173589 -1.261157

H -4.877420 -3.527777 -2.035909

H -3.059221 0.581058 -1.958384

C 4.413360 4.291992 -1.288201

H 3.676429 3.893984 -1.993894

H 5.246804 4.734696 -1.836524

H 3.945529 5.047054 -0.648712

H -1.743541 -1.169774 -0.704930

C -1.960999 8.922292 0.585301

H -2.522784 8.705428 1.502368

H -2.583408 8.629906 -0.269583

C -0.611071 8.171517 0.571894

H -0.057822 8.443011 -0.337828

H -0.002084 8.516851 1.418959

C -0.730028 6.632551 0.641388

H -1.284081 6.347910 1.546629

H -1.304453 6.264869 -0.219494

C 0.653547 5.967542 0.661144

H 1.220445 6.193246 -0.245350

H 1.236940 6.300182 1.523450

N 0.594130 4.460776 0.756738

H 0.176587 4.026840 -0.072013

H 1.546957 4.018640 0.877642

H 0.028151 4.160141 1.555522

H -1.797574 10.004442 0.533424

C 9.857863 -5.008771 -0.494197

H 10.212256 -4.851730 0.531639

H 10.374578 -4.294336 -1.146229

C 8.327675 -4.841765 -0.587716

H 8.009113 -5.038605 -1.620583

H 7.838329 -5.592736 0.046406

C 7.817400 -3.448561 -0.177142

H 8.064758 -3.244523 0.871396

H 8.289740 -2.676746 -0.797841

N 6.351787 -3.392078 -0.344159

H 5.924309 -4.174721 -0.823731

C 5.530886 -2.386396 0.006384

N 5.996810 -1.264621 0.565225

N 4.206708 -2.508024 -0.190498

H 6.977546 -1.007996 0.539953

H 5.370787 -0.475213 0.750627

H 3.804647 -3.396291 -0.456393

H 3.554763 -1.757929 0.062725

H 10.143366 -6.019858 -0.802212

C 8.085993 1.779373 -0.222698

H 7.811907 2.842427 -0.172202

C 7.532599 1.162574 -1.505240

O 7.633720 1.105567 0.959288

H 7.840851 0.115135 -1.603413

H 6.438846 1.210651 -1.513396

H 7.905393 1.711767 -2.379636

H 6.681940 1.328449 1.085393

H 9.179718 1.713980 -0.208622

------------------------------------

PC3

*G*(Water) =-3748.180824 Hartree

------------------------------------

C -2.882970 -4.200247 4.117511

C -2.415524 -3.458146 2.911888

C -2.373556 -2.120155 2.629718

N -1.931207 -4.094982 1.773293

C -1.608978 -3.192985 0.839139

N -1.876152 -1.990262 1.345880

H -3.738524 -4.841593 3.876903

H -3.191428 -3.490357 4.888757

H -1.827338 -5.096999 1.658810

H -2.666801 -1.255568 3.206802

H -1.197842 -3.385715 -0.138178

P 4.566005 1.063104 -1.939761

S 0.128552 -0.267244 -1.138905

O 3.667882 2.371379 -1.644052

O 4.624479 0.186552 -0.671448

O 4.122099 0.413343 -3.224751

O 6.078321 1.657287 -2.219443

O 0.077291 -1.748675 -1.210537

O 0.360261 0.420798 -2.423862

O 0.890837 0.267231 0.009507

C -4.698643 -0.410987 -0.561156

N -6.391973 0.717941 -1.986605

O -3.718852 1.862269 -2.071368

S -3.772296 1.547662 2.685341

C -5.174480 -0.080863 -1.987776

O -3.603397 0.491574 1.376497

C -4.082762 0.673760 -2.767075

O -3.129843 2.812757 2.249350

C -3.297714 1.765225 -0.695728

O -3.032403 0.798050 3.727739

C -4.248702 0.872275 0.148183

O -5.234430 1.657706 2.909395

C -1.810365 1.464414 -0.535664

O -1.514138 0.057203 -0.760102

C -7.607710 0.253856 -2.380083

O -2.991125 -0.145467 -3.103760

C -8.750739 1.248430 -2.308659

O -7.790650 -0.906990 -2.771571

C -5.445906 -1.960669 1.140353

O -5.784464 -1.029219 0.111141

H -3.854274 -1.108584 -0.631540

H -5.401858 -1.013044 -2.506095

H -5.153831 1.441917 0.383502

H -4.499060 1.028111 -3.712185

H -6.312065 1.693129 -1.727206

H -3.397547 2.787907 -0.322950

H -1.261252 2.066506 -1.265037

H -1.497168 1.725323 0.476080

H -8.440418 2.255282 -2.014874

H -9.239464 1.294478 -3.286830

H -9.486849 0.881122 -1.585532

H -5.031624 -1.457268 2.017767

H -2.087950 -4.833378 4.527263

H -6.375567 -2.467841 1.410019

H -4.724737 -2.705311 0.775336

H -2.439019 -0.301369 -2.309219

C 6.725092 2.455138 -1.214119

H 6.164498 3.380762 -1.034225

H 7.714420 2.707505 -1.602976

H 6.830771 1.900569 -0.275671

H -1.778057 -1.115595 0.824530

C 3.433119 8.565067 2.240618

H 4.335967 8.495297 2.861017

H 2.568139 8.364228 2.885872

C 3.490320 7.569964 1.071789

H 2.590149 7.685519 0.450907

H 4.344821 7.816002 0.424909

C 3.605622 6.104455 1.520389

H 4.511024 5.974762 2.131746

H 2.750652 5.847003 2.163160

C 3.654631 5.129803 0.336012

H 2.754344 5.244821 -0.279892

H 4.511458 5.365893 -0.307136

N 3.757946 3.696966 0.694004

H 2.973731 3.426133 1.288421

H 3.740560 2.782305 -0.706116

H 4.599614 3.530319 1.246573

H 3.351523 9.597189 1.879275

C 5.094456 -7.859193 -0.189114

H 5.873061 -7.691232 0.564856

H 5.579672 -7.912315 -1.171385

C 4.040122 -6.745787 -0.151869

H 3.252648 -6.946089 -0.889714

H 3.560763 -6.725765 0.836245

C 4.657425 -5.375213 -0.435981

H 5.464064 -5.179878 0.284310

H 5.087882 -5.359682 -1.444623

N 3.641073 -4.319383 -0.334003

H 2.757205 -4.564799 0.095251

C 3.887019 -3.012564 -0.508338

N 5.092286 -2.585813 -0.909102

N 2.929554 -2.100699 -0.279483

H 5.904736 -3.181676 -0.844048

H 5.225824 -1.568739 -0.961948

H 1.945044 -2.342233 -0.339678

H 3.177476 -1.118285 -0.423512

H 4.633903 -8.832294 0.012196

C 6.731787 -0.994934 1.717985

H 7.015186 -1.371908 0.724566

C 5.793025 -1.976685 2.416212

O 6.162405 0.312738 1.603095

H 5.583931 -1.643617 3.439705

H 4.840098 -2.050113 1.881636

H 6.240146 -2.978370 2.460653

H 5.560503 0.318916 0.820367

H 7.656407 -0.883045 2.296645

------------------------------------

TS3

*G*(Water) =-3748.145055 Hartree

------------------------------------

C -0.989793 -5.075532 -3.553945

C -0.856598 -3.677621 -3.043005

C -1.093574 -3.128334 -1.809748

N -0.455586 -2.617102 -3.847547

C -0.454182 -1.479455 -3.135205

N -0.841191 -1.770730 -1.897552

H -1.727791 -5.130516 -4.362689

H -1.317169 -5.732940 -2.744620

H -0.201688 -2.682888 -4.826472

H -1.428748 -3.586561 -0.891839

H -0.175325 -0.498638 -3.486288

P 4.020803 2.096928 -0.654284

S 0.849730 1.192238 -0.775960

O 4.475679 3.552974 -0.424327

O 4.050593 1.241494 0.624813

O 2.660527 2.055481 -1.428839

O 5.048784 1.386429 -1.740427

O 0.308035 1.364934 -2.132273

O 0.650258 2.247546 0.240824

O 1.486800 -0.076192 -0.377088

C -4.144394 -0.526886 -0.430213

N -6.000154 0.751597 0.620548

O -3.335455 1.290550 1.662207

S -2.841065 -3.451439 1.766848

C -4.809937 0.843868 -0.217205

O -2.830454 -2.234662 0.616736

C -3.816592 1.834153 0.428594

O -2.248098 -2.858556 2.993305

C -2.690324 -0.007817 1.630622

O -1.988959 -4.460011 1.088912

C -3.584309 -1.039740 0.899860

O -4.265614 -3.844743 1.922852

C -1.239269 0.044083 1.135290

O -1.113132 0.295425 -0.245138

C -7.266752 0.961097 0.179982

O -2.807989 2.233909 -0.450250

C -8.360604 0.849072 1.225834

O -7.537730 1.222414 -1.001655

C -4.619844 -2.374550 -1.900715

O -5.118374 -1.417370 -0.964457

H -3.321402 -0.398656 -1.141218

H -5.127343 1.237874 -1.183398

H -4.435631 -1.303812 1.537264

H -4.364664 2.731968 0.729242

H -5.856508 0.607921 1.612297

H -2.641844 -0.297811 2.683798

H -0.737464 0.835852 1.710662

H -0.765475 -0.909938 1.403796

H -8.957463 1.766437 1.211340

H -9.020477 0.018576 0.952702

H -7.985272 0.681966 2.239542

H -3.953912 -3.097163 -1.419890

H -0.035384 -5.451827 -3.940031

H -5.493271 -2.890300 -2.308500

H -4.079517 -1.878782 -2.720267

H -2.103494 1.503767 -0.496119

C 6.400989 1.144071 -1.327938

H 6.911344 2.081887 -1.074906

H 6.910782 0.673738 -2.172712

H 6.428596 0.469532 -0.464098

H -0.964987 -1.019387 -1.141678

C -0.721002 8.822381 0.070912

H -0.457070 9.123705 1.092561

H -1.663627 8.262380 0.119622

C 0.392617 7.974278 -0.555590

H 0.126831 7.718563 -1.590595

H 1.315801 8.568349 -0.608136

C 0.669444 6.683985 0.230801

H 0.879683 6.937369 1.279782

H -0.222958 6.043433 0.227252

C 1.857914 5.920381 -0.346625

H 1.668465 5.587578 -1.370639

H 2.755601 6.545023 -0.351643

N 2.193764 4.695121 0.445890

H 1.500700 3.935834 0.329361

H 3.132732 4.285994 0.131668

H 2.258761 4.904637 1.445342

H -0.900001 9.732344 -0.513635

C 7.265890 -5.690684 1.698579

H 6.813612 -5.686470 2.697834

H 8.103533 -4.982637 1.702967

C 6.236759 -5.321602 0.624607

H 6.703332 -5.349789 -0.368680

H 5.418566 -6.053887 0.621308

C 5.667029 -3.926612 0.874467

H 5.188961 -3.896706 1.862848

H 6.483109 -3.192330 0.867582

N 4.688044 -3.561086 -0.154307

H 4.436662 -4.262276 -0.839592

C 4.093604 -2.359975 -0.227518

N 4.305584 -1.441944 0.714762

N 3.289876 -2.060238 -1.269546

H 4.967923 -1.634922 1.452000

H 4.065036 -0.433136 0.582487

H 3.003964 -2.799876 -1.897604

H 2.625505 -1.291820 -1.149890

H 7.672296 -6.691610 1.517649

C 3.180553 0.379569 3.712515

H 4.047909 -0.245158 3.445030

C 1.892212 -0.272530 3.213861

O 3.355354 1.704567 3.202733

H 1.019302 0.310860 3.530683

H 1.883561 -0.330997 2.120969

H 1.792530 -1.289259 3.617351

H 3.560890 1.624446 2.236297

H 3.168639 0.466932 4.805499

------------------------------------

RC-W213

*G*(Water) =-4151.335123 Hartree

------------------------------------

C 0.706641 -6.013816 -2.803948

C 0.608779 -4.691792 -2.108510

C 1.563202 -3.771479 -1.739601

N -0.588125 -4.133747 -1.686324

C -0.326463 -2.936976 -1.094166

N 0.972278 -2.686133 -1.114625

H 0.308638 -6.825137 -2.182247

H 1.754506 -6.235742 -3.024375

H -1.506037 -4.547902 -1.793047

H 2.633529 -3.830732 -1.882501

H -1.086520 -2.312353 -0.645275

P -3.868880 2.058300 1.519738

S -1.711131 0.028133 1.629349

O -2.864955 3.182749 1.631776

O -4.551681 1.832968 0.180027

O -3.240343 0.610426 2.042011

O -4.981070 2.146304 2.706677

O -1.925562 -1.428989 1.655219

O -0.811365 0.554558 2.686052

O -1.419563 0.597246 0.286845

C 3.969988 -0.238824 -1.882341

N 6.111826 1.012705 -1.764597

O 4.486678 0.973568 0.693271

S 0.919951 2.111413 -2.258087

C 5.384010 -0.152375 -1.277533

O 1.775955 0.739287 -1.829370

C 5.325329 -0.100050 0.264719

O 0.938179 2.979333 -1.042220

C 3.138338 1.057960 0.176692

O -0.389633 1.528593 -2.606901

C 3.125491 0.928683 -1.365896

O 1.661843 2.718246 -3.390912

C 2.143232 0.146617 0.900124

O 2.352687 -1.252370 0.678121

C 7.172453 0.950588 -2.611490

O 5.000258 -1.338525 0.837776

C 7.796030 2.280457 -2.991058

O 7.616178 -0.116198 -3.059395

C 3.157647 -1.034841 -3.999393

O 4.082174 -0.198997 -3.298577

H 3.517215 -1.186180 -1.570849

H 5.952500 -1.033157 -1.578713

H 3.527856 1.846316 -1.808467

H 6.315211 0.165470 0.643351

H 5.841676 1.918475 -1.402427

H 2.833322 2.079030 0.421495

H 2.242502 0.321171 1.976073

H 1.130622 0.422614 0.596253

H 7.340320 3.139181 -2.489842

H 8.863658 2.250539 -2.751481

H 7.701666 2.412764 -4.074134

H 2.122121 -0.742941 -3.796581

H 0.150787 -6.013364 -3.749582

H 3.375671 -0.909169 -5.062963

H 3.297363 -2.089790 -3.725062

H 4.028385 -1.499386 0.730569

C -6.021623 3.138210 2.580320

H -5.597750 4.148628 2.592177

H -6.674910 3.006773 3.444507

H -6.591466 2.983878 1.658143

H 1.801951 -1.618690 -0.085657

C 2.664049 5.958565 4.834571

H 3.599661 5.881961 4.265997

H 2.661524 5.158340 5.585476

C 1.442556 5.848900 3.909480

H 0.528675 5.959298 4.510316

H 1.446917 6.686307 3.197783

C 1.361000 4.525794 3.123442

H 2.223936 4.428857 2.451144

H 1.397652 3.677812 3.821705

C 0.059063 4.468980 2.325323

H -0.800307 4.576915 2.992631

H 0.010975 5.270598 1.583166

N -0.161449 3.184238 1.584787

H 0.017331 2.350916 2.161729

H -1.180620 3.115427 1.357636

H 0.367424 3.098278 0.699947

H 2.667582 6.918836 5.362989

C -7.855668 -2.139337 -4.984949

H -8.461828 -1.320776 -4.578320

H -8.118434 -3.057056 -4.444745

C -6.355614 -1.830994 -4.857691

H -5.767750 -2.645637 -5.301406

H -6.112013 -0.915605 -5.412058

C -5.953361 -1.649236 -3.392782

H -6.555957 -0.845077 -2.955324

H -6.157000 -2.572261 -2.833275

N -4.534984 -1.284396 -3.258961

H -3.911687 -1.521689 -4.021423

C -4.002316 -0.718742 -2.164190

N -4.709125 -0.639063 -1.022874

N -2.749395 -0.247785 -2.173716

H -5.635476 -1.041751 -1.000364

H -4.585502 0.203785 -0.437102

H -2.252036 -0.069612 -3.035729

H -2.310800 0.100073 -1.316453

H -8.132895 -2.275686 -6.035711

C -3.280950 3.286475 -2.836215

H -2.717176 2.349914 -2.912074

C -2.511540 4.275618 -1.963873

O -4.603745 3.031917 -2.339818

H -3.032346 5.240151 -1.915220

H -2.407815 3.887161 -0.943902

H -1.504833 4.438626 -2.367152

H -4.527014 2.640187 -1.440565

H -3.412903 3.685838 -3.848588

C -0.530596 -6.530295 2.642602

H -0.088489 -7.288120 3.304003

H -0.415667 -6.880208 1.610498

C 0.110933 -5.187002 2.829567

C 0.785788 -4.446092 1.884513

C 0.143379 -4.409154 4.043194

H 0.977767 -4.671826 0.843637

N 1.248314 -3.265471 2.435680

C 0.857848 -3.207300 3.753482

C -0.364069 -4.605021 5.342363

H 1.689003 -2.508826 1.909004

C 1.062385 -2.211096 4.718132

C -0.161262 -3.617189 6.304551

H -0.909371 -5.513431 5.589119

H 1.601750 -1.298835 4.475753

C 0.544436 -2.430505 5.993732

H -0.550334 -3.756138 7.310388

H 0.684423 -1.675644 6.763635

H -1.605476 -6.500363 2.867950

------------------------------------

PC-W213

*G*(Water) =-4151.333252 Hartree

------------------------------------

C 0.855959 5.961732 0.002712

C 0.291512 4.586660 -0.115489

C -0.836855 4.119964 -0.733305

N 0.894533 3.468636 0.453084

C 0.174174 2.371679 0.193530

N -0.880274 2.754026 -0.523642

H 0.931205 6.267765 1.052338

H 0.207263 6.670214 -0.517866

H 1.758791 3.470419 0.983542

H -1.618974 4.628626 -1.278022

H 0.401196 1.366689 0.507886

P 6.393053 -0.500558 -0.924780

S -1.516235 -0.340152 -2.271281

O 7.501056 0.618709 -1.248969

O 6.169647 -0.583848 0.590676

O 5.183985 -0.277185 -1.812017

O 7.059468 -1.918220 -1.432362

O -0.289889 -0.119538 -1.460795

O -1.987801 -1.735242 -2.321922

O -1.553246 0.398925 -3.547182

C -3.928818 1.902249 1.358943

N -5.943670 1.076375 2.562331

O -5.460643 -0.128761 -0.033590

S -4.665600 4.311047 -1.615051

C -4.617346 0.727254 2.076520

O -3.854986 3.150395 -0.690331

C -4.733488 -0.479924 1.132798

O -5.504373 3.532790 -2.560592

C -5.016425 0.997440 -0.819159

O -3.514266 5.011747 -2.230186

C -4.672485 2.234191 0.058219

O -5.440775 5.112574 -0.637380

C -3.966010 0.624993 -1.860002

O -2.645477 0.474286 -1.265931

C -6.259378 1.212355 3.878467

O -3.476067 -1.063582 0.864308

C -7.702911 1.567369 4.178536

O -5.431505 1.063132 4.787069

C -2.833912 3.918378 2.124169

O -3.926296 3.004565 2.251021

H -2.898851 1.601684 1.130599

H -4.018352 0.446047 2.943578

H -5.603252 2.735827 0.343658

H -5.331969 -1.258867 1.609326

H -6.687239 1.158853 1.880386

H -5.894541 1.256167 -1.415796

H -4.260331 -0.322881 -2.320032

H -3.915724 1.408718 -2.616241

H -8.337561 1.616185 3.289133

H -8.111748 0.824793 4.870930

H -7.724744 2.539731 4.681999

H -2.884260 4.477869 1.186407

H 1.856828 6.017931 -0.440168

H -2.913817 4.606081 2.969391

H -1.871951 3.390227 2.182545

H -2.987237 -0.535080 0.196020

C 8.226789 -2.413776 -0.757960

H 9.078305 -1.738111 -0.904552

H 8.459825 -3.383792 -1.202466

H 8.037966 -2.536930 0.314163

H -1.614683 2.123032 -0.852096

C 14.533883 1.617390 -0.637671

H 14.839784 0.766330 -0.015401

H 14.584299 2.520110 -0.015072

C 13.122041 1.411862 -1.192307

H 12.851375 2.262433 -1.834408

H 13.105121 0.520257 -1.835547

C 12.068728 1.257729 -0.088260

H 12.325691 0.400648 0.551714

H 12.075444 2.148453 0.557280

C 10.663711 1.058200 -0.657388

H 10.390071 1.910556 -1.291068

H 10.643254 0.168495 -1.298806

N 9.589880 0.899591 0.346097

H 9.543141 1.719817 0.951019

H 8.244746 0.707030 -0.534740

H 9.784234 0.105366 0.956410

H 15.269645 1.724702 -1.443982

C -1.015115 -3.101217 3.154908

H -0.688809 -2.637214 4.093846

H -0.552369 -4.094007 3.091539

C -0.639623 -2.235384 1.952478

H -0.981443 -2.714333 1.025315

H -1.148517 -1.266895 2.011080

C 0.868362 -2.013601 1.872039

H 1.219025 -1.489498 2.770204

H 1.386022 -2.981974 1.821619

N 1.186337 -1.214557 0.689807

H 0.455324 -1.069836 -0.006913

C 2.417882 -0.804943 0.358202

N 3.462582 -1.064962 1.150923

N 2.597922 -0.056396 -0.749012

H 3.411473 -1.764824 1.879054

H 4.418007 -0.790538 0.877838

H 1.808180 0.034709 -1.380228

H 3.541067 -0.045509 -1.171508

H -2.100740 -3.238124 3.210765

C 6.211495 -2.158869 3.724182

H 5.739449 -1.193015 3.964401

C 7.730882 -2.036249 3.812318

O 5.771506 -2.612696 2.438821

H 8.209159 -3.000014 3.601258

H 8.102061 -1.301585 3.087232

H 8.034174 -1.709454 4.815188

H 6.037996 -1.945041 1.764012

H 5.842581 -2.895146 4.446601

C -2.960359 -7.466604 -0.276072

H -2.142974 -7.571003 0.446201

H -2.588449 -7.803652 -1.253268

C -3.454398 -6.051062 -0.331712

C -3.004303 -4.978017 0.406281

C -4.507433 -5.540877 -1.174180

H -2.221852 -4.941632 1.153139

N -3.716300 -3.837588 0.084497

C -4.637541 -4.148777 -0.889283

C -5.350936 -6.120068 -2.142513

H -3.535959 -2.893161 0.425965

C -5.574197 -3.334462 -1.542088

C -6.282876 -5.315490 -2.795444

H -5.273842 -7.179441 -2.377337

H -5.657438 -2.277771 -1.302986

C -6.392007 -3.936013 -2.497290

H -6.938739 -5.750489 -3.545808

H -7.129107 -3.333692 -3.022982

H -3.757439 -8.163190 0.017937

------------------------------------

TS-W213

*G*(Water) =-4151.282828 Hartree

------------------------------------

C 3.843419 5.540164 -2.475129

C 3.154979 4.222013 -2.342002

C 3.283188 3.214021 -1.421986

N 2.189966 3.780719 -3.239682

C 1.756960 2.561914 -2.878518

N 2.411576 2.201433 -1.780281

H 4.393061 5.606445 -3.421321

H 4.554961 5.667559 -1.655358

H 1.856694 4.293595 -4.047484

H 3.915597 3.139250 -0.550764

H 1.000488 1.973905 -3.374167

P -3.679361 0.071605 -0.906831

S -0.398116 0.018059 -1.242618

O -4.312412 -1.334040 -0.965486

O -3.408394 0.566863 0.526799

O -2.472097 0.229439 -1.878147

O -4.764216 1.143135 -1.580121

O 0.001151 -0.013900 -2.656685

O -0.650400 -1.222701 -0.510046

O -0.429296 1.309145 -0.532047

C 4.867011 -0.803397 -1.092718

N 5.888855 -3.008785 -0.565751

O 3.295754 -2.475719 0.650053

S 5.175078 1.745576 1.819524

C 4.796772 -2.332107 -1.255179

O 4.564124 1.005635 0.446098

C 3.452256 -2.871957 -0.724892

O 4.362072 1.231153 2.951779

C 3.350836 -1.055821 0.969548

O 4.936308 3.169455 1.477206

C 4.634973 -0.430498 0.374403

O 6.606518 1.352097 1.884834

C 2.057799 -0.301111 0.631742

O 1.832406 -0.139992 -0.748003

C 6.907879 -3.652011 -1.191338

O 2.371383 -2.533266 -1.531414

C 7.929136 -4.316050 -0.286536

O 7.019326 -3.701907 -2.425133

C 6.139285 0.896504 -2.220572

O 6.142748 -0.361219 -1.542261

H 4.075645 -0.358268 -1.704607

H 4.882750 -2.580229 -2.313743

H 5.506739 -0.769278 0.945264

H 3.503961 -3.963740 -0.677755

H 5.836359 -3.062989 0.443738

H 3.444614 -1.042482 2.058666

H 1.228546 -0.859288 1.089096

H 2.117356 0.673458 1.137471

H 7.685884 -4.247257 0.777648

H 8.015209 -5.370631 -0.566844

H 8.903636 -3.846014 -0.456939

H 5.854914 1.712631 -1.549111

H 3.127059 6.369274 -2.441759

H 7.158347 1.054193 -2.583447

H 5.450455 0.878465 -3.077713

H 2.108366 -1.567323 -1.343983

C -5.903012 1.528483 -0.806072

H -6.528731 0.663978 -0.547717

H -6.490758 2.215671 -1.420609

H -5.596388 2.032167 0.117935

H 2.242011 1.252643 -1.285206

C -11.231841 -3.094741 -2.349173

H -11.725064 -2.634289 -1.483737

H -11.201296 -4.178661 -2.180398

C -9.819941 -2.527949 -2.543093

H -9.359984 -2.980752 -3.432375

H -9.881336 -1.448275 -2.738327

C -8.914189 -2.770307 -1.326287

H -9.369605 -2.317682 -0.434410

H -8.834201 -3.849275 -1.134813

C -7.521698 -2.183582 -1.538939

H -7.018009 -2.640084 -2.395037

H -7.567249 -1.104758 -1.708539

N -6.619025 -2.390244 -0.358092

H -6.461453 -3.385917 -0.179867

H -5.653719 -1.918973 -0.534594

H -7.023582 -1.997431 0.496049

H -11.855663 -2.908572 -3.231129

C -4.125830 8.029750 2.664759

H -3.592999 7.773925 3.588771

H -5.146828 7.636570 2.743079

C -3.407769 7.460937 1.437813

H -3.943952 7.743851 0.522591

H -2.396565 7.883100 1.366694

C -3.315022 5.938411 1.517228

H -2.774456 5.649735 2.428799

H -4.324857 5.510724 1.568256

N -2.624627 5.395632 0.344215

H -2.210911 6.053063 -0.304407

C -2.458693 4.085713 0.106942

N -2.858219 3.176685 0.996180

N -1.905121 3.670892 -1.051822

H -3.333352 3.477906 1.834793

H -2.969448 2.166750 0.742941

H -1.471319 4.356354 -1.656663

H -1.478901 2.740463 -1.062316

H -4.189834 9.121619 2.605014

C -2.352616 0.726669 3.720901

H -2.741358 1.758190 3.685035

C -0.981197 0.662486 3.050846

O -3.295802 -0.176520 3.137571

H -0.576316 -0.354148 3.105331

H -1.048680 0.950130 1.996558

H -0.279568 1.344363 3.550200

H -3.355759 0.023666 2.170143

H -2.276860 0.446549 4.778033

C -1.472010 -2.819149 4.763984

H -0.784291 -2.369388 5.489171

H -1.936594 -3.693369 5.240703

C -0.754942 -3.190048 3.500376

C 0.575806 -3.000025 3.204336

C -1.340312 -3.791002 2.327304

H 1.355414 -2.555164 3.809714

N 0.863442 -3.468363 1.933806

C -0.298757 -3.938627 1.363546

C -2.645513 -4.200708 1.991180

H 1.728403 -3.263807 1.432629

C -0.530941 -4.478314 0.092124

C -2.881172 -4.735821 0.726322

H -3.457881 -4.092077 2.706502

H 0.273646 -4.559490 -0.633923

C -1.831793 -4.873663 -0.213564

H -3.885703 -5.049437 0.451799

H -2.047310 -5.287591 -1.195760

H -2.269952 -2.093883 4.560043

------------------------------------

RC-W213G(M5)

*G*(Water) =-4151.335123 Hartree

------------------------------------

C -1.819694 -1.185247 -5.543624

C -1.261541 -0.472103 -4.354648

C -1.781882 -0.211159 -3.108833

N 0.000773 0.100213 -4.331958

C 0.196256 0.671117 -3.112591

N -0.870110 0.499653 -2.346685

H -1.891313 -0.522397 -6.415015

H -2.823778 -1.553010 -5.313453

H 0.669594 0.093501 -5.092321

H -2.736279 -0.505821 -2.703304

H 1.108720 1.176070 -2.824122

P 4.594460 -1.475092 -0.420515

S 3.066436 1.075016 -0.409166

O 6.108836 -1.415130 -0.492273

O 3.954499 -1.674474 0.938358

O 4.019031 -0.100658 -1.154603

O 3.986026 -2.527173 -1.501206

O 2.815747 1.994522 -1.536013

O 3.933608 1.639845 0.662241

O 1.869834 0.370903 0.100875

C -4.368575 1.665239 -0.198159

N -5.916106 2.519493 1.546632

O -3.181424 1.990756 2.423114

S -3.692913 -2.114693 -0.002433

C -4.734689 2.824027 0.748641

O -3.351421 -0.511379 -0.303485

C -3.568486 3.160397 1.700242

O -3.297210 -2.362533 1.405071

C -2.811198 0.809311 1.673529

O -2.803370 -2.764510 -1.009216

C -3.891464 0.460054 0.618187

O -5.139552 -2.283888 -0.271589

C -1.380828 0.864152 1.127276

O -1.210549 1.766632 0.037272

C -7.118961 3.130892 1.394625

O -2.509308 3.815465 1.057576

C -8.220388 2.668886 2.330179

O -7.326837 4.010558 0.546419

C -5.312314 0.771681 -2.243189

O -5.529465 1.373049 -0.964595

H -3.556632 1.993566 -0.856006

H -4.971038 3.704917 0.150702

H -4.764881 0.032276 1.122003

H -3.932230 3.843555 2.471381

H -5.811336 1.847587 2.296505

H -2.815283 0.014157 2.424572

H -0.724622 1.181341 1.945181

H -1.085243 -0.144662 0.829450

H -7.885990 1.946824 3.080574

H -8.643600 3.542312 2.835947

H -9.016346 2.212304 1.731964

H -5.070738 -0.291267 -2.148819

H -1.199364 -2.044093 -5.828924

H -6.248475 0.885896 -2.796675

H -4.507512 1.282028 -2.787979

H -2.012485 3.164757 0.495485

C 4.217017 -3.936421 -1.285954

H 5.289898 -4.156264 -1.273272

H 3.745956 -4.452990 -2.123617

H 3.755427 -4.260782 -0.347546

H -1.117782 1.264679 -0.829740

C 9.433864 5.188685 0.640688

H 9.858115 4.993111 1.633672

H 8.555195 5.832855 0.772643

C 9.056538 3.880575 -0.061992

H 8.668037 4.097390 -1.066595

H 9.956818 3.265708 -0.200233

C 8.011421 3.076484 0.723705

H 8.387099 2.885494 1.738934

H 7.089484 3.664621 0.826511

C 7.702059 1.748662 0.041883

H 7.270539 1.893901 -0.951859

H 8.602552 1.137033 -0.059641

N 6.712243 0.934214 0.819485

H 5.768044 1.364687 0.831667

H 6.585135 -0.015386 0.381008

H 7.011745 0.802913 1.789257

H 10.177234 5.748574 0.061265

C -1.556690 -7.046350 2.115369

H -1.564616 -6.675519 3.147891

H -0.638047 -7.628926 1.973163

C -1.638517 -5.891342 1.113616

H -1.643337 -6.280049 0.086934

H -2.572698 -5.332358 1.253312

C -0.455738 -4.940642 1.284332

H -0.461185 -4.525097 2.302514

H 0.480060 -5.499583 1.152299

N -0.512939 -3.860238 0.301428

H -1.404999 -3.677900 -0.164596

C 0.393483 -2.879563 0.229488

N 1.469808 -2.886537 1.022905

N 0.231584 -1.885782 -0.665798

H 1.642686 -3.674664 1.629786

H 2.277692 -2.268213 0.869958

H -0.674807 -1.780452 -1.110140

H 0.789734 -1.036872 -0.564220

H -2.407356 -7.726615 1.997199

C 1.957597 -0.747051 3.833769

H 1.256046 -1.263856 3.163475

C 1.871223 0.762280 3.625205

O 3.285904 -1.254681 3.638994

H 2.560814 1.284300 4.300008

H 2.125059 1.020952 2.592443

H 0.852890 1.117618 3.831350

H 3.503406 -1.251689 2.683092

H 1.688701 -1.008863 4.863532

C -2.669666 3.832091 -2.770661

H -3.727487 3.712499 -3.025368

H -2.585448 4.221124 -1.751451

H -2.165735 2.863261 -2.831005

H -2.203841 4.533243 -3.470560

------------------------------------

PC-W213G(M5)

*G*(Water) =-4151.333252 Hartree

------------------------------------

C -5.674580 -3.192072 3.220796

C -4.510014 -2.274328 3.077098

C -4.430321 -0.911554 2.973468

N -3.200678 -2.730151 2.973520

C -2.361575 -1.697908 2.810627

N -3.098800 -0.590805 2.808859

H -5.767403 -3.846327 2.346279

H -6.594502 -2.609713 3.312992

H -2.911853 -3.701075 3.011139

H -5.192465 -0.150243 2.971728

H -1.287776 -1.740143 2.691143

P 3.981777 -0.104539 -1.747606

S 0.273274 0.683866 1.998033

O 3.346926 1.305315 -2.219510

O 3.620047 -0.347996 -0.279314

O 3.683577 -1.216093 -2.732862

O 5.610323 0.140377 -1.841480

O 0.602097 -0.748137 2.193790

O 1.422486 1.615909 1.998643

O -0.872655 1.157209 2.817546

C -2.725174 -0.479892 -1.440322

N -2.806391 -0.152921 -3.902274

O -0.831142 1.429705 -2.522963

S -4.238834 2.345172 0.753408

C -2.017154 -0.665529 -2.790722

O -3.231592 1.089697 0.285447

C -0.651489 0.038961 -2.778534

O -3.523555 3.591244 0.378016

C -1.544909 1.836325 -1.337037

O -4.314175 2.082377 2.211630

C -2.846158 1.011087 -1.097966

O -5.508953 2.132881 0.014754

C -0.644718 1.968471 -0.117356

O -0.301925 0.668420 0.419879

C -3.376857 -0.933629 -4.856542

O 0.271390 -0.596469 -1.935521

C -4.140197 -0.199701 -5.942742

O -3.294146 -2.170043 -4.854232

C -4.593342 -1.544434 -0.332786

O -3.994605 -1.105006 -1.552284

H -2.129075 -0.974513 -0.661501

H -1.867924 -1.731529 -2.965767

H -3.647419 1.423378 -1.721171

H -0.220915 0.003820 -3.781976

H -2.871830 0.852231 -4.002999

H -1.845308 2.863971 -1.558354

H 0.272787 2.488982 -0.405665

H -1.175130 2.543804 0.640999

H -4.075458 0.889468 -5.865672

H -3.755163 -0.513913 -6.917882

H -5.193489 -0.495413 -5.889533

H -4.976608 -0.704805 0.251584

H -5.575231 -3.825355 4.109652

H -5.417379 -2.204943 -0.615148

H -3.872954 -2.109350 0.273870

H 0.112638 -0.365909 -0.996314

C 6.202624 1.169939 -1.034730

H 5.856278 2.160605 -1.353941

H 7.283112 1.103618 -1.180782

H 5.968867 1.019934 0.025630

H -2.705445 0.342036 2.664291

C 3.143548 8.001529 1.400795

H 4.205353 7.979174 1.677836

H 2.557093 7.787937 2.303713

C 2.841041 6.975216 0.292424

H 1.780445 7.048475 0.011740

H 3.415813 7.237000 -0.607929

C 3.154403 5.518773 0.679901

H 4.210073 5.437961 0.980337

H 2.553931 5.230904 1.553712

C 2.888211 4.526187 -0.466294

H 1.840576 4.592513 -0.784035

H 3.499585 4.799825 -1.335683

N 3.164951 3.105194 -0.155690

H 2.521998 2.754630 0.558608

H 3.225499 1.970152 -1.447251

H 4.095866 3.023118 0.255759

H 2.900465 9.018963 1.072358

C 2.095011 -8.643105 0.876958

H 3.181325 -8.789345 0.911175

H 1.738001 -8.972869 -0.106193

C 1.724181 -7.171236 1.139133

H 0.631522 -7.061400 1.133135

H 2.070395 -6.883418 2.140967

C 2.323237 -6.194649 0.114562

H 3.417344 -6.284015 0.112732

H 1.958108 -6.431263 -0.892752

N 1.945096 -4.812633 0.459417

H 1.403827 -4.687178 1.305703

C 2.322069 -3.690562 -0.181247

N 3.127784 -3.740273 -1.251726

N 1.880945 -2.492397 0.226804

H 3.494471 -4.617737 -1.590515

H 3.339384 -2.886355 -1.787336

H 1.325233 -2.387491 1.068146

H 2.421626 -1.650748 -0.032571

H 1.640953 -9.287424 1.636967

C 4.636194 -1.963266 2.412281

H 3.727411 -2.528231 2.156872

C 4.258100 -0.683029 3.155452

O 5.408010 -1.712735 1.237166

H 5.158188 -0.117560 3.427046

H 3.617843 -0.048005 2.534686

H 3.708953 -0.922905 4.075676

H 4.826366 -1.198416 0.623943

H 5.252262 -2.606454 3.052280

C -0.267007 4.942080 1.808985

H -0.003282 5.754099 2.493957

H 0.303036 4.044012 2.062283

H -1.337395 4.726882 1.879596

H -0.028276 5.242662 0.784235

------------------------------------

TS-W213G(M5)

*G*(Water) =-4151.282828 Hartree

------------------------------------

C 0.852978 -5.109337 3.640271

C 0.751521 -3.723279 3.091184

C 1.005987 -3.213678 1.844533

N 0.368682 -2.632494 3.863525

C 0.394696 -1.515437 3.119709

N 0.781691 -1.849200 1.892820

H 1.586658 -5.158230 4.453305

H 1.169340 -5.794818 2.850063

H 0.107787 -2.665134 4.842182

H 1.336187 -3.704590 0.941916

H 0.134563 -0.519620 3.441637

P -3.985923 2.116084 0.535207

S -0.842743 1.115156 0.679594

O -4.388907 3.578873 0.256128

O -4.048638 1.219319 -0.714256

O -2.627974 2.050309 1.311446

O -5.038436 1.480142 1.644132

O -0.295384 1.309178 2.029912

O -0.615396 2.133916 -0.367506

O -1.515525 -0.145923 0.317732

C 4.111006 -0.716650 0.380033

N 5.989499 0.483782 -0.721525

O 3.332863 1.035426 -1.778539

S 2.765133 -3.699486 -1.707616

C 4.803172 0.631448 0.113979

O 2.766151 -2.439443 -0.604766

C 3.828136 1.615933 -0.567592

O 2.196860 -3.142757 -2.962403

C 2.662403 -0.247679 -1.695485

O 1.886798 -4.665245 -1.001338

C 3.539900 -1.268143 -0.929744

O 4.183277 -4.125635 -1.832773

C 1.214629 -0.148593 -1.197056

O 1.098686 0.153376 0.173865

C 7.260801 0.686803 -0.291701

O 2.830439 2.069376 0.297220

C 8.349301 0.519090 -1.335719

O 7.539854 0.985526 0.879113

C 4.550590 -2.510497 1.925047

O 5.067429 -1.605092 0.948144

H 3.291347 -0.544881 1.085613

H 5.130372 1.055454 1.064049

H 4.385347 -1.568685 -1.558687

H 4.393162 2.490508 -0.903334

H 5.840757 0.308636 -1.707452

H 2.603745 -0.576400 -2.736686

H 0.726735 0.632459 -1.798535

H 0.720583 -1.101696 -1.430585

H 7.968122 0.314076 -2.340243

H 8.955923 1.429829 -1.361701

H 9.001405 -0.306276 -1.030449

H 3.862529 -3.233782 1.477490

H -0.110634 -5.454851 4.032174

H 5.412910 -3.032663 2.348001

H 4.029295 -1.968078 2.727216

H 2.112533 1.355891 0.375069

C -6.396949 1.266316 1.236712

H -6.874924 2.209289 0.942696

H -6.924554 0.849110 2.098307

H -6.444931 0.557649 0.401571

H 0.923275 -1.122831 1.115091

C 0.965048 8.662995 -0.443282

H 0.700660 8.951148 -1.468583

H 1.885710 8.067343 -0.489218

C -0.170814 7.870116 0.214477

H 0.099589 7.625786 1.250669

H -1.071198 8.498139 0.266622

C -0.504633 6.574849 -0.540378

H -0.725679 6.812932 -1.590623

H 0.366150 5.905579 -0.540416

C -1.705502 5.863412 0.076318

H -1.511993 5.564764 1.109397

H -2.586962 6.510603 0.066722

N -2.079274 4.617457 -0.664315

H -1.407855 3.844066 -0.518247

H -3.028369 4.248186 -0.330835

H -2.142370 4.786881 -1.671361

H 1.184112 9.578315 0.118789

C -7.465083 -5.649400 -1.521486

H -6.998458 -5.728854 -2.510943

H -8.267753 -4.904556 -1.584336

C -6.435521 -5.262614 -0.454139

H -6.917210 -5.205477 0.530597

H -5.654435 -6.031787 -0.390528

C -5.794830 -3.914906 -0.780352

H -5.301902 -3.968638 -1.760609

H -6.574783 -3.144507 -0.832330

N -4.816742 -3.530362 0.242142

H -4.566419 -4.219240 0.940277

C -4.190299 -2.343411 0.268598

N -4.373291 -1.459167 -0.710931

N -3.385885 -2.020689 1.303137

H -5.020316 -1.672236 -1.456132

H -4.110909 -0.451503 -0.616094

H -3.134784 -2.736479 1.972193

H -2.693838 -1.282769 1.152210

H -7.921612 -6.616927 -1.286436

C -3.219295 0.225467 -3.769652

H -4.102155 -0.365264 -3.476906

C -1.946716 -0.441865 -3.251141

O -3.356685 1.572628 -3.309054

H -1.060111 0.106454 -3.591786

H -1.935108 -0.460801 -2.156913

H -1.875396 -1.474896 -3.617581

H -3.558474 1.533578 -2.339241

H -3.209535 0.271866 -4.865171

C 0.854407 4.702861 2.524280

H 0.367084 3.782377 2.191724

H 1.678181 4.453116 3.200828

H 1.250269 5.244432 1.659874

H 0.133284 5.336137 3.051035

------------------------------------

RC-D214

*G*(Water) =-4017.359454 Hartree

------------------------------------

C 2.525806 -2.441048 -5.703570

C 2.088281 -2.584213 -4.284014

C 1.617075 -1.677626 -3.365772

N 2.140390 -3.788559 -3.598115

C 1.716395 -3.577905 -2.327775

N 1.391440 -2.303850 -2.150128

H 3.590060 -2.681431 -5.820361

H 2.374526 -1.409262 -6.033287

H 2.446040 -4.675863 -3.978825

H 1.450365 -0.616655 -3.482119

H 1.662348 -4.358419 -1.581833

P -4.986177 0.011971 0.933503

S -2.431419 1.498982 0.642592

O -5.718952 0.986823 0.033003

O -4.654365 -1.364618 0.374335

O -3.623367 0.699928 1.557556

O -5.730544 -0.133607 2.377034

O -1.245527 1.297597 1.497114

O -2.903898 2.894706 0.536779

O -2.393947 0.767867 -0.653689

C 4.631651 -0.080935 -0.805371

N 6.403838 1.037900 0.521513

O 3.854080 0.474383 1.929015

S 1.751380 2.159521 -2.062120

C 5.615379 -0.177955 0.367855

O 2.497062 0.768552 -1.502488

C 4.868190 -0.491330 1.683639

O 1.303210 2.888689 -0.846890

C 2.868983 0.722973 0.903857

O 0.662211 1.573599 -2.880456

C 3.516900 0.924977 -0.492271

O 2.786284 2.884857 -2.843817

C 1.729058 -0.296234 0.923010

O 2.094778 -1.582727 0.392891

C 7.642362 1.007139 1.056683

O 4.409080 -1.823608 1.745128

C 8.334578 2.348365 1.210433

O 8.201716 -0.046722 1.419438

C 4.856612 -0.212979 -3.205362

O 5.371522 0.280084 -1.965790

H 4.187053 -1.072596 -0.953658

H 6.305908 -1.002495 0.175999

H 3.944959 1.929369 -0.548285

H 5.563807 -0.374797 2.514950

H 5.997888 1.960336 0.287773

H 2.419479 1.675734 1.198144

H 1.428665 -0.456078 1.962575

H 0.871514 0.103700 0.378771

H 7.749024 3.175657 0.801839

H 8.515291 2.526358 2.276208

H 9.308709 2.303722 0.711959

H 3.928767 0.293198 -3.484500

H 1.959085 -3.100032 -6.372853

H 5.627795 -0.017751 -3.955463

H 4.668838 -1.294407 -3.150386

H 3.630899 -1.926466 1.147843

C -6.887342 -0.991191 2.466424

H -7.719337 -0.583708 1.880787

H -7.166378 -1.016875 3.521200

H -6.650086 -2.001804 2.119299

H 1.914471 -1.627454 -0.585939

C -12.149996 4.405344 -0.604931

H -12.855932 3.566490 -0.558156

H -12.073445 4.721707 -1.652891

C -10.778021 4.001204 -0.051111

H -10.099360 4.864708 -0.082788

H -10.875816 3.718179 1.006029

C -10.150643 2.836328 -0.831163

H -10.818105 1.964544 -0.791048

H -10.048685 3.116276 -1.888844

C -8.782943 2.458351 -0.271666

H -8.078954 3.292174 -0.330575

H -8.846662 2.133096 0.769742

N -8.156058 1.322000 -1.031118

H -8.047470 1.550593 -2.023386

H -7.196198 1.097892 -0.636230

H -8.730225 0.475839 -0.978540

H -12.576363 5.236969 -0.032070

C 1.389851 -6.043464 3.174179

H 0.577933 -6.780288 3.133636

H 1.361501 -5.565044 4.161125

C 1.253786 -5.009368 2.054158

H 2.091332 -4.300127 2.100631

H 1.308316 -5.502936 1.075306

C -0.063034 -4.231714 2.139162

H -0.913107 -4.917727 2.046898

H -0.138100 -3.727813 3.114208

N -0.120427 -3.260137 1.047197

H 0.769925 -2.832311 0.774140

C -1.214560 -2.578565 0.671051

N -2.357537 -2.658264 1.358945

N -1.166511 -1.851803 -0.459843

H -2.358536 -3.012853 2.304800

H -3.212624 -2.192908 1.017839

H -0.324122 -1.901792 -1.036048

H -1.748011 -1.015774 -0.556379

H 2.339098 -6.583950 3.090173

C -5.306575 -4.649356 0.310814

H -4.892018 -4.412125 1.302818

C -4.174156 -4.901360 -0.682909

O -6.186741 -3.603924 -0.114809

H -4.577582 -5.180933 -1.663486

H -3.557846 -4.003528 -0.803743

H -3.524426 -5.712535 -0.329272

H -5.703163 -2.752567 -0.009007

H -5.925569 -5.547342 0.418622

C 6.647443 1.380397 4.555081

H 7.344278 0.805413 3.938304

H 5.755901 1.617880 3.968452

H 6.363621 0.792124 5.433522

H 7.126829 2.310234 4.877225

C 4.839386 3.881009 2.143629

H 4.188580 4.503253 2.764562

H 5.848539 3.891563 2.574711

C 4.908246 4.396571 0.700443

O 4.453319 5.544971 0.446189

O 5.450557 3.616673 -0.152323

H 4.496011 2.842316 2.167890

------------------------------------

PC-D214

*G*(Water) =-4017.369779 Hartree

------------------------------------

C 6.472980 -1.435962 4.360551

C 5.047367 -1.354717 3.926775

C 4.174094 -2.314142 3.488619

N 4.331477 -0.162390 3.893370

C 3.082660 -0.382173 3.458095

N 2.976369 -1.684406 3.211667

H 7.113699 -0.816764 3.722455

H 6.818629 -2.470542 4.296693

H 4.691841 0.747833 4.156845

H 4.298534 -3.372089 3.330425

H 2.299644 0.348271 3.307075

P -3.658476 2.496499 -0.767165

S -0.378297 -0.454928 2.070932

O -3.448264 0.973739 -0.930800

O -2.377298 3.204619 -0.205963

O -4.278189 3.207297 -1.970188

O -4.818319 2.670650 0.439925

O 0.330304 0.791949 2.456522

O -1.855996 -0.371820 2.090545

O 0.159591 -1.676105 2.726887

C 2.711635 -0.706875 -1.109378

N 2.998312 -0.751514 -3.575519

O 0.312607 -1.379496 -2.580063

S 2.448455 -4.238435 0.538494

C 2.355349 -0.063939 -2.463272

O 2.289076 -2.580881 0.326725

C 0.832504 -0.059175 -2.697130

O 1.256104 -4.843999 -0.107826

C 0.578192 -2.126781 -1.377288

O 2.457957 -4.316884 2.019565

C 2.088278 -2.104511 -1.015759

O 3.733203 -4.600070 -0.112138

C -0.352296 -1.801370 -0.214302

O 0.058010 -0.580995 0.457997

C 3.999518 -0.218893 -4.323445

O 0.161067 0.894127 -1.920793

C 4.519987 -1.090737 -5.450508

O 4.474370 0.906039 -4.111584

C 4.669598 -0.513295 0.276355

O 4.128840 -0.766178 -1.020359

H 2.310295 -0.081257 -0.304123

H 2.717948 0.964682 -2.469501

H 2.630840 -2.754734 -1.711268

H 0.634098 0.214759 -3.734618

H 2.628739 -1.655227 -3.842884

H 0.317513 -3.150620 -1.652898

H -1.378106 -1.696770 -0.573383

H -0.298811 -2.628961 0.494868

H 3.976315 -2.032944 -5.564720

H 4.461814 -0.527499 -6.387220

H 5.575550 -1.312780 -5.260595

H 4.403345 -1.309136 0.976935

H 6.591628 -1.094446 5.395052

H 5.755449 -0.478820 0.157708

H 4.320397 0.453433 0.666398

H 0.211671 0.678983 -0.967830

C -4.499437 2.227990 1.762876

H -3.862582 1.336101 1.755133

H -5.441541 1.990484 2.269028

H -3.981440 3.018082 2.316205

H 2.129491 -2.114898 2.830180

C -8.942109 -3.150643 1.771415

H -8.698283 -3.198261 2.840444

H -8.692447 -4.122549 1.326947

C -8.171285 -2.013592 1.079428

H -8.463685 -1.970241 0.020633

H -8.469105 -1.052060 1.521332

C -6.640361 -2.151347 1.167734

H -6.330713 -2.178483 2.222258

H -6.325750 -3.102310 0.716357

C -5.936228 -0.994885 0.454181

H -6.210488 -0.970960 -0.603899

H -6.207816 -0.033237 0.899551

N -4.447129 -1.097805 0.483579

H -4.101870 -1.972465 -0.007589

H -4.066017 -1.099416 1.432898

H -10.024327 -3.003584 1.677549

C 4.875378 6.719161 -0.788825

H 4.305270 7.634265 -0.587826

H 4.995859 6.625520 -1.874947

C 4.167909 5.485530 -0.200250

H 4.775690 4.589823 -0.387215

H 4.079430 5.592593 0.888741

C 2.768073 5.272950 -0.790215

H 2.146210 6.154216 -0.592383

H 2.836180 5.142265 -1.878634

N 2.119710 4.098953 -0.184526

H 2.705778 3.448993 0.324680

C 0.807567 3.816480 -0.250878

N -0.024451 4.574393 -0.987984

N 0.276040 2.799941 0.431139

H 0.327668 5.157474 -1.733273

H -1.008678 4.278484 -1.009499

H 0.756481 2.296301 1.168900

H -0.744287 2.670072 0.346107

H 5.870373 6.835098 -0.346332

C -1.447020 5.062663 2.485218

H -0.542801 5.085299 1.858540

C -1.423317 3.825555 3.381318

O -2.614469 5.150141 1.669571

H -2.305793 3.803116 4.032036

H -1.403391 2.905563 2.789405

H -0.526412 3.834697 4.014998

H -2.576720 4.425413 0.988538

H -1.435939 5.969839 3.101954

C -2.273172 -0.024609 -4.654778

H -1.503625 -0.603005 -5.175757

H -1.794011 0.680658 -3.970181

H -2.876257 0.523995 -5.385745

H -2.912703 -0.704098 -4.083656

H -4.019071 -0.267880 -0.026918

C -2.546346 -3.573633 -3.082487

H -2.978851 -3.718914 -4.076795

H -2.120744 -4.513925 -2.717569

C -3.580931 -3.017537 -2.100347

O -4.557336 -2.363209 -2.553126

O -3.354389 -3.220511 -0.857020

H -1.728259 -2.847736 -3.170259

------------------------------------

TS-D214

*G*(Water) =-4017.320926 Hartree

------------------------------------

C -4.654982 4.237777 3.347065

C -3.885768 2.997826 3.024870

C -3.779351 2.264127 1.871515

N -3.088153 2.339671 3.954164

C -2.526069 1.260077 3.385359

N -2.938218 1.195973 2.125116

H -5.383681 4.057044 4.145876

H -5.195944 4.574706 2.459133

H -2.944609 2.624186 4.916093

H -4.234464 2.413608 0.904326

H -1.837926 0.572651 3.852687

P 3.475066 0.283377 1.543474

S 0.197693 -0.449478 1.488732

O 4.476667 -0.869836 1.372247

O 3.185473 1.053786 0.228384

O 2.197171 -0.111056 2.339381

O 4.166875 1.394494 2.580218

O -0.261896 -0.972566 2.782143

O 0.706966 -1.367347 0.465966

O 0.025038 0.976660 1.195650

C -4.961089 -1.627421 0.490502

N -5.748125 -3.705099 -0.622544

O -3.081451 -2.794265 -1.361936

S -5.116825 1.485553 -1.827451

C -4.792104 -3.148526 0.328215

O -4.618482 0.491537 -0.576499

C -3.359770 -3.489419 -0.141215

O -4.138432 1.281462 -2.927119

C -3.203274 -1.349037 -1.364405

O -5.034117 2.808787 -1.160151

C -4.589838 -0.928667 -0.820538

O -6.495293 1.045423 -2.166958

C -2.017115 -0.633569 -0.703441

O -1.965136 -0.770908 0.696652

C -6.788105 -4.509164 -0.286262

O -2.404244 -3.293089 0.856243

C -7.650044 -4.994404 -1.437363

O -7.040156 -4.837524 0.882825

C -6.481286 -0.281797 1.779095

O -6.311190 -1.359377 0.856518

H -4.283172 -1.291930 1.281680

H -4.976249 -3.631344 1.288747

H -5.363435 -1.173373 -1.556980

H -3.328438 -4.545773 -0.424641

H -5.576453 -3.530992 -1.604883

H -3.167918 -1.088118 -2.425732

H -1.104893 -1.044633 -1.159615

H -2.077892 0.426460 -0.990276

H -7.300343 -4.662073 -2.419112

H -7.679932 -6.088552 -1.419420

H -8.671942 -4.631223 -1.284908

H -6.180047 0.673816 1.338978

H -3.989397 5.045564 3.672804

H -7.545079 -0.254824 2.029723

H -5.899640 -0.454365 2.696494

H -2.197699 -2.295460 0.922559

C 5.486144 1.851949 2.272473

H 6.215096 1.035994 2.355923

H 5.735420 2.631171 2.997955

H 5.539066 2.276435 1.261116

H -2.601130 0.416328 1.447861

C 11.281611 -3.637876 0.999123

H 11.862262 -2.708176 0.944581

H 11.269553 -4.084702 -0.003211

C 9.855124 -3.366794 1.498270

H 9.306974 -4.316208 1.576232

H 9.896997 -2.947600 2.513519

C 9.073216 -2.407025 0.588327

H 9.620872 -1.457532 0.500692

H 8.999754 -2.828909 -0.423113

C 7.668517 -2.131141 1.123521

H 7.082954 -3.053038 1.188146

H 7.707107 -1.683996 2.121477

N 6.900831 -1.202941 0.238820

H 6.736622 -1.636050 -0.723571

H 5.951490 -0.983259 0.659566

H 7.403099 -0.321973 0.109054

H 11.809549 -4.326567 1.669059

C 2.079299 8.680510 -2.224010

H 2.607191 8.520034 -3.172167

H 2.820411 8.974992 -1.470768

C 1.323334 7.423982 -1.792181

H 0.787633 7.611379 -0.852472

H 0.570087 7.164894 -2.548554

C 2.267496 6.237465 -1.596853

H 2.801450 6.022668 -2.532523

H 3.011054 6.473334 -0.826039

N 1.502041 5.063101 -1.173658

H 0.495088 5.105899 -1.269009

C 2.017717 3.935916 -0.667105

N 3.350157 3.751813 -0.587345

N 1.222508 2.976106 -0.199142

H 3.969244 4.272264 -1.193238

H 3.647680 2.815447 -0.286032

H 0.227037 3.104484 -0.090345

H 1.643053 2.095251 0.119988

H 1.389153 9.519887 -2.362255

C 2.313936 1.148297 -3.121375

H 2.402914 2.213911 -2.860533

C 0.895016 0.656801 -2.841518

O 3.308731 0.386322 -2.430525

H 0.779798 -0.388104 -3.149877

H 0.662023 0.723452 -1.774431

H 0.162140 1.264600 -3.388684

H 3.232568 0.559958 -1.458569

H 2.545930 1.054366 -4.188728

C 0.832423 -3.219724 -2.581042

H -0.089719 -2.999341 -3.128139

H 0.889507 -4.294825 -2.381221

H 1.694932 -2.915829 -3.181924

H 0.833295 -2.668670 -1.637317

C 4.532840 -3.306962 -1.215221

H 4.325887 -2.401309 -0.634702

H 5.061278 -4.000569 -0.547565

C 5.424714 -2.998699 -2.423884

O 6.479930 -2.306554 -2.194823

O 5.096160 -3.445272 -3.552321

H 3.590173 -3.766131 -1.522881

------------------------------------

RC-D214A(M6)

*G*(Water) =-4271.621937 Hartree

------------------------------------

C -3.567346 -6.923128 0.331813

C -2.613713 -5.775567 0.397211

C -1.497179 -5.455383 -0.340063

N -2.723201 -4.750706 1.321901

C -1.699678 -3.875531 1.123376

N -0.937189 -4.274692 0.117243

H -3.574100 -7.494878 1.268089

H -3.276827 -7.599605 -0.476995

H -3.431935 -4.674693 2.041346

H -1.067330 -6.003513 -1.167576

H -1.569832 -2.983138 1.719706

P -2.542053 2.818969 1.406119

S -1.594446 0.041396 1.802809

O -1.372570 3.643648 1.910580

O -2.690293 2.617538 -0.084257

O -2.585396 1.341112 2.185910

O -3.935815 3.333188 2.079957

O -1.950128 -0.922670 2.863164

O -0.212037 0.554197 1.852149

O -2.013722 -0.389498 0.438868

C 3.998769 -1.606351 -1.619317

N 5.882753 -0.905540 -0.157249

O 3.269159 -1.258603 1.149024

S 1.621261 1.326844 -2.503689

C 4.945569 -1.979976 -0.462624

O 2.126797 -0.243278 -2.238096

C 4.147610 -2.330755 0.811706

O 0.729353 1.652783 -1.350339

C 2.327721 -0.800067 0.150844

O 0.903319 1.182784 -3.786572

C 3.064636 -0.480509 -1.170344

O 2.849383 2.157888 -2.541038

C 1.084215 -1.670266 0.021647

O 1.333768 -2.919682 -0.638554

C 7.214300 -0.961275 -0.420729

O 3.515696 -3.579951 0.726647

C 8.020958 0.261301 -0.027119

O 7.755012 -1.938828 -0.957922

C 4.295331 -1.630730 -4.003365

O 4.786287 -1.201328 -2.731998

H 3.401025 -2.487309 -1.878980

H 5.539535 -2.845737 -0.757621

H 3.671607 0.420339 -1.031694

H 4.839658 -2.387192 1.655654

H 5.522584 -0.091547 0.325414

H 1.952813 0.134833 0.566257

H 0.709182 -1.848573 1.034329

H 0.329900 -1.098924 -0.525232

H 7.455062 0.990774 0.557334

H 8.894816 -0.062027 0.546309

H 8.381746 0.749400 -0.939313

H 3.310804 -1.200709 -4.216496

H -4.593310 -6.584694 0.140723

H 5.017515 -1.282533 -4.746379

H 4.233235 -2.727773 -4.049163

H 2.704406 -3.497105 0.156277

C -4.427620 4.627226 1.674887

H -3.709645 5.413864 1.931969

H -5.357339 4.787007 2.223646

H -4.629446 4.644452 0.598017

H 0.535394 -3.492931 -0.440725

C 6.093426 3.666521 0.539391

H 6.109916 3.108974 -0.405571

H 6.341451 2.965284 1.346058

C 4.719740 4.303308 0.779777

H 4.730160 4.846522 1.735193

H 4.513044 5.047966 -0.001422

C 3.592951 3.262111 0.795349

H 3.532092 2.771466 -0.184364

H 3.826613 2.483471 1.534889

C 2.240636 3.888346 1.130305

H 2.252212 4.365991 2.114124

H 1.955195 4.638844 0.387889

N 1.156552 2.860793 1.146536

H 1.335275 2.116511 1.824704

H 0.213278 3.245466 1.407736

H 1.051248 2.400604 0.219628

H 6.883905 4.424909 0.498452

C -9.908839 0.121822 -1.467109

H -9.905657 0.499621 -2.496938

H -9.846161 0.983054 -0.790707

C -8.748849 -0.853053 -1.229484

H -8.784679 -1.231991 -0.199918

H -8.856784 -1.720535 -1.895130

C -7.378033 -0.207793 -1.466328

H -7.297863 0.158571 -2.497885

H -7.241135 0.644952 -0.792300

N -6.315744 -1.185562 -1.192946

H -6.563880 -2.164778 -1.267982

C -5.073073 -0.905103 -0.777196

N -4.601392 0.345546 -0.805688

N -4.283101 -1.899120 -0.323643

H -4.948350 1.047031 -1.456984

H -3.672276 0.535287 -0.430836

H -4.701534 -2.748530 0.032256

H -3.369919 -1.635490 0.051781

H -10.869122 -0.375373 -1.293161

C -3.134443 2.507424 -3.311131

H -2.605844 1.588184 -3.015961

C -2.141231 3.652612 -3.481434

O -4.155367 2.809353 -2.345839

H -2.654921 4.565435 -3.805640

H -1.629571 3.857856 -2.534152

H -1.381990 3.392860 -4.229327

H -3.697299 2.895428 -1.475715

H -3.657502 2.303606 -4.251774

C 1.055198 -2.692206 3.739066

H 1.887922 -2.172946 3.257500

H 0.927081 -3.675481 3.274206

H 1.263297 -2.817575 4.806923

H 0.141053 -2.106750 3.608447

C 1.214294 1.092818 4.810178

C 2.493905 0.899593 3.989854

H 2.959130 1.862430 3.741547

H 3.239260 0.315514 4.543658

H 2.292808 0.359097 3.058424

H 1.419850 1.621457 5.749153

H 0.466861 1.666580 4.251345

H 0.761221 0.125738 5.057742

------------------------------------

PC-D214A(M6)

*G*(Water) =-4271.632925 Hartree

------------------------------------

C 3.854158 -0.582754 6.285020

C 3.244505 -0.545092 4.925180

C 3.082882 -1.515682 3.973197

N 2.692028 0.606483 4.374879

C 2.212880 0.352640 3.149090

N 2.445071 -0.932234 2.897686

H 4.692109 0.119460 6.361013

H 4.227149 -1.588344 6.493613

H 2.649573 1.512525 4.827465

H 3.367869 -2.554505 3.956550

H 1.742479 1.055432 2.476167

P -6.202641 1.162735 -0.417198

S 0.886986 0.386436 -0.276687

O -7.489242 0.479907 0.125992

O -6.002998 2.587232 0.165605

O -4.948931 0.279056 -0.353183

O -6.438252 1.367822 -2.060794

O 1.083101 1.698125 0.394916

O -0.121205 0.416017 -1.366043

O 0.751127 -0.763007 0.644136

C 5.325607 0.004905 -0.230670

N 7.235800 0.060213 -1.815881

O 4.629864 -0.485580 -3.002767

S 3.867774 -3.528123 0.588720

C 6.005846 0.727292 -1.408607

O 3.967875 -1.865526 0.379883

C 5.070682 0.818582 -2.630426

O 3.382252 -4.066840 -0.706775

C 3.996355 -1.319531 -2.011638

O 2.881749 -3.581654 1.696256

C 4.800510 -1.365419 -0.680945

O 5.238183 -3.968522 0.948884

C 2.514859 -1.022704 -1.822312

O 2.356205 0.183399 -1.030686

C 8.479562 0.574833 -1.630390

O 4.032423 1.753231 -2.472784

C 9.629990 -0.281551 -2.124587

O 8.681448 1.670046 -1.087548

C 5.790910 -0.197154 2.135266

O 6.294757 -0.110002 0.800612

H 4.479966 0.611488 0.116030

H 6.278365 1.734904 -1.092452

H 5.663720 -2.028915 -0.804397

H 5.649365 1.161739 -3.490223

H 7.150330 -0.819520 -2.309098

H 4.026134 -2.317011 -2.456070

H 2.035465 -0.876622 -2.793321

H 2.050978 -1.859358 -1.298497

H 9.310439 -1.172191 -2.673353

H 10.271632 0.326934 -2.769145

H 10.226473 -0.595268 -1.260962

H 5.338637 -1.172427 2.330333

H 3.119731 -0.319308 7.054700

H 6.648874 -0.048779 2.795908

H 5.048668 0.590268 2.327428

H 3.371390 1.420526 -1.829322

C -7.550410 2.159850 -2.485425

H -8.498108 1.726705 -2.140011

H -7.539234 2.173542 -3.579317

H -7.467851 3.188440 -2.111036

H 2.199956 -1.391659 2.020113

C -10.965116 -5.982386 -0.404713

H -10.307643 -6.594584 0.225536

H -10.742070 -6.219907 -1.452617

C -10.760451 -4.486746 -0.123813

H -11.451162 -3.899093 -0.744388

H -11.019522 -4.270833 0.922054

C -9.320379 -4.023493 -0.393394

H -8.625982 -4.600386 0.233389

H -9.057468 -4.232474 -1.439944

C -9.136301 -2.532515 -0.113282

H -9.790541 -1.923936 -0.743712

H -9.349402 -2.292361 0.932019

N -7.735975 -2.069187 -0.381102

H -7.473568 -2.235444 -1.356299

H -7.061158 -2.578762 0.194992

H -12.000130 -6.282283 -0.203692

C 0.971238 6.618725 -1.098947

H 0.847786 7.175748 -0.161832

H 0.347489 7.099991 -1.862407

C 0.589472 5.143291 -0.921810

H 0.740383 4.601265 -1.864468

H 1.248622 4.675860 -0.178479

C -0.868105 4.963140 -0.480590

H -1.036815 5.481460 0.474469

H -1.539134 5.406924 -1.226175

N -1.177002 3.536699 -0.354661

H -0.390099 2.905258 -0.202762

C -2.409841 3.002604 -0.286193

N -3.499785 3.784486 -0.216257

N -2.545964 1.669423 -0.304400

H -3.400139 4.745382 0.081216

H -4.438494 3.350670 -0.100777

H -1.771663 1.117654 -0.671389

H -3.482008 1.215937 -0.301800

H 2.017399 6.711294 -1.410424

C -5.128038 3.723406 3.243157

H -4.865370 4.577706 2.598070

C -3.966113 2.730926 3.283737

O -6.347337 3.126941 2.800147

H -4.197031 1.894879 3.955077

H -3.767644 2.326865 2.285029

H -3.051070 3.221217 3.641530

H -6.240866 2.885377 1.840681

H -5.322597 4.120619 4.246503

C -0.437552 -1.624062 -4.315312

H 0.452316 -2.258301 -4.382097

H -0.409593 -1.052604 -3.384028

H -0.460441 -0.940084 -5.170076

H -1.331850 -2.255134 -4.327642

C -0.581644 -3.476580 -1.043112

C -1.887690 -2.674655 -1.018018

H -2.582510 -3.014533 -1.796488

H -2.396617 -2.774887 -0.050688

H -1.687471 -1.611281 -1.183318

H -0.080555 -3.379862 -2.013685

H 0.110420 -3.110875 -0.277543

H -0.759797 -4.544639 -0.864046

H -7.608925 -0.989510 -0.177909

------------------------------------

TS-D214A(M6)

*G*(Water) =-4271.585697 Hartree

------------------------------------

C 4.242675 4.353366 -3.218905

C 3.430166 3.173253 -2.799025

C 3.389433 2.466552 -1.625416

N 2.514005 2.548415 -3.636998

C 1.948499 1.510950 -2.997164

N 2.470374 1.444759 -1.777813

H 4.877738 4.111191 -4.079203

H 4.887355 4.666981 -2.393940

H 2.298524 2.825887 -4.587569

H 3.939983 2.603992 -0.707447

H 1.189913 0.850375 -3.387286

P -3.829445 -0.018514 -0.538711

S -0.561658 -0.296824 -0.811043

O -4.429295 -1.399416 -0.210743

O -3.606970 0.837445 0.724380

O -2.602362 -0.064186 -1.499306

O -4.926467 0.793234 -1.489149

O -0.166591 -0.705674 -2.165506

O -0.901464 -1.301085 0.200830

O -0.483839 1.124914 -0.434232

C 4.679869 -1.407599 -0.431101

N 5.556312 -3.522144 0.542414

O 2.990005 -2.614096 1.571326

S 5.125739 1.648864 1.909183

C 4.516696 -2.930978 -0.292233

O 4.459151 0.684563 0.715362

C 3.134067 -3.269152 0.305859

O 4.340088 1.375455 3.140630

C 3.137460 -1.172305 1.616683

O 4.906527 2.990940 1.314582

C 4.456514 -0.740543 0.929094

O 6.550999 1.239506 2.009798

C 1.890567 -0.418750 1.133029

O 1.666697 -0.507354 -0.252070

C 6.531687 -4.346746 0.083495

O 2.092740 -3.022066 -0.588546

C 7.493597 -4.883132 1.127534

O 6.652344 -4.652166 -1.112454

C 6.066109 -0.033491 -1.837462

O 5.984163 -1.137675 -0.934590

H 3.922844 -1.047529 -1.135576

H 4.599291 -3.387875 -1.279154

H 5.306240 -1.002478 1.569497

H 3.108996 -4.334271 0.555367

H 5.487129 -3.371961 1.541236

H 3.233661 -0.955193 2.684202

H 1.034166 -0.839431 1.682490

H 2.001115 0.629156 1.447539

H 7.242001 -4.588567 2.150470

H 7.510147 -5.975697 1.063175

H 8.500373 -4.520841 0.893400

H 5.838840 0.912134 -1.336039

H 3.603696 5.199292 -3.498206

H 7.093127 -0.016379 -2.211790

H 5.376461 -0.165986 -2.683928

H 1.878356 -2.027020 -0.578630

C -6.137157 1.246552 -0.873242

H -6.703376 0.413062 -0.437449

H -6.739244 1.713399 -1.657086

H -5.923540 1.980590 -0.087452

H 2.191009 0.671533 -1.063121

C -10.939008 -4.145184 0.930004

H -10.798233 -5.194613 0.641471

H -11.506754 -3.651226 0.131268

C -9.591228 -3.451345 1.155904

H -9.761107 -2.411982 1.469735

H -9.056497 -3.943232 1.980240

C -8.705491 -3.461125 -0.098608

H -8.508881 -4.497652 -0.405596

H -9.237396 -2.974820 -0.928189

C -7.384791 -2.740979 0.149442

H -7.547969 -1.698455 0.434979

H -6.805485 -3.221941 0.941950

N -6.500171 -2.712510 -1.060440

H -6.968104 -2.274168 -1.857958

H -5.598512 -2.148528 -0.826782

H -6.232194 -3.656340 -1.351362

H -11.550472 -4.125337 1.839655

C -3.238167 8.657311 1.432766

H -2.719811 8.461845 2.379428

H -4.310406 8.490956 1.593360

C -2.701582 7.759542 0.313899

H -3.216578 7.983937 -0.629287

H -1.632724 7.955205 0.156170

C -2.898874 6.285158 0.659343

H -2.382328 6.058505 1.601591

H -3.968169 6.078011 0.797403

N -2.374548 5.421113 -0.401772

H -1.921065 5.862435 -1.191594

C -2.421146 4.081201 -0.366874

N -2.918317 3.443714 0.690136

N -1.989620 3.357608 -1.424771

H -3.307554 3.969566 1.459035

H -3.127566 2.417519 0.659660

H -1.458435 3.834813 -2.142376

H -1.684100 2.399601 -1.238901

H -3.095767 9.714411 1.183578

C -2.367826 1.468591 3.704278

H -2.986097 2.337138 3.425921

C -0.964082 1.617300 3.119591

O -3.008517 0.258731 3.292307

H -0.325555 0.783702 3.436603

H -0.997131 1.624803 2.025652

H -0.502781 2.554544 3.459103

H -3.222298 0.356987 2.328890

H -2.325158 1.439974 4.799666

C -0.168025 -5.011072 1.464139

H 0.444225 -4.441815 0.758914

H -1.058544 -4.429940 1.724384

H -0.474531 -5.958536 1.008882

H 0.411174 -5.214031 2.371044

C -3.994376 -5.300776 -1.894477

C -2.866830 -4.352508 -1.468478

H -3.264723 -3.408780 -1.080356

H -2.247142 -4.802903 -0.683812

H -2.208671 -4.117229 -2.314496

H -4.603437 -4.862353 -2.696337

H -3.601421 -6.252606 -2.272504

H -4.656689 -5.536548 -1.050807

------------------------------------

RC-W304

*G*(Water) =-4151.329242 Hartree

------------------------------------

C 2.789096 4.489695 5.385733

C 2.502327 3.221080 4.645893

C 1.335384 2.662759 4.174731

N 3.485241 2.308188 4.298398

C 2.898363 1.262106 3.653643

N 1.592124 1.449355 3.558528

H 3.230913 4.289833 6.369787

H 1.860106 5.046738 5.537124

H 4.472661 2.393292 4.506911

H 0.331960 3.060875 4.243627

H 3.448001 0.411210 3.274354

P 4.232472 -1.818900 -1.899458

S 3.587386 -1.611628 0.994657

O 3.559990 -3.147223 -2.185762

O 3.487541 -0.564919 -2.322568

O 4.671859 -1.708502 -0.302295

O 5.765174 -1.803444 -2.446191

O 4.520325 -1.587129 2.138359

O 2.734461 -2.814992 0.898780

O 2.849656 -0.339580 0.784776

C -2.602010 0.833678 2.172014

N -4.850571 -0.202757 1.894929

O -2.590528 -2.056936 2.205587

S -0.599583 0.680747 -1.146312

C -3.736759 0.009839 2.810841

O -0.861052 0.756551 0.506796

C -3.221088 -1.367028 3.286161

O -0.323105 -0.757597 -1.438299

C -1.534572 -1.388952 1.472201

O 0.559979 1.582347 -1.287026

C -1.984828 0.022894 1.029210

O -1.852660 1.155932 -1.780379

C -0.174874 -1.441385 2.172153

O -0.113925 -0.677328 3.377493

C -6.090871 0.324871 2.059020

O -2.437747 -1.287430 4.446389

C -7.126918 -0.084058 1.031520

O -6.377230 1.095880 2.987499

C -2.273058 3.186395 1.830272

O -3.144043 2.061956 1.709035

H -1.839309 1.023694 2.935654

H -4.130199 0.554935 3.669679

H -2.739895 -0.066641 0.241390

H -4.081129 -1.992591 3.537297

H -4.711863 -0.855857 1.134593

H -1.429630 -1.996333 0.569705

H 0.031300 -2.488108 2.422085

H 0.592996 -1.103132 1.473921

H -6.783144 -0.868726 0.352196

H -8.023978 -0.426791 1.556385

H -7.392604 0.795174 0.437394

H -1.365244 3.057161 1.231725

H 3.485050 5.131553 4.831566

H -2.834442 4.050629 1.465302

H -1.995102 3.356496 2.881074

H -1.542444 -0.930777 4.208360

C 5.973516 -1.599718 -3.860224

H 5.486285 -2.388439 -4.444185

H 7.052376 -1.643256 -4.017672

H 5.594644 -0.619696 -4.167851

H 0.489127 0.118509 3.303065

C -3.027247 -6.440489 -1.840404

H -3.768215 -5.632438 -1.889925

H -2.961549 -6.771060 -0.796025

C -1.661060 -5.968706 -2.354330

H -0.951570 -6.807412 -2.319507

H -1.747057 -5.678478 -3.410802

C -1.077745 -4.790447 -1.555691

H -1.755107 -3.927421 -1.607755

H -0.991184 -5.068730 -0.496802

C 0.295845 -4.405023 -2.100261

H 0.977796 -5.259913 -2.075116

H 0.227882 -4.059874 -3.135496

N 0.963283 -3.309859 -1.329754

H 1.084379 -3.532839 -0.337145

H 1.943268 -3.167045 -1.689139

H 0.463963 -2.405204 -1.381722

H -3.402442 -7.279624 -2.437538

C 8.201434 5.647747 -2.516768

H 7.578666 6.161924 -3.259067

H 8.696093 4.805138 -3.015165

C 7.362656 5.168724 -1.325165

H 8.007609 4.670277 -0.590029

H 6.905438 6.031467 -0.821984

C 6.263173 4.195779 -1.758748

H 5.584018 4.684849 -2.470117

H 6.713937 3.330778 -2.259102

N 5.511740 3.714932 -0.592770

H 5.560563 4.267376 0.254614

C 4.753008 2.607435 -0.571093

N 4.491623 1.941715 -1.695088

N 4.238299 2.170863 0.593773

H 4.658669 2.342226 -2.608988

H 4.051113 1.010744 -1.702420

H 4.649258 2.483642 1.463193

H 3.723802 1.283824 0.631849

H 8.976967 6.346647 -2.185484

C 2.109045 1.842476 -4.357227

H 1.966341 2.241579 -3.343851

C 0.951281 0.913147 -4.708682

O 3.386999 1.192293 -4.453336

H 1.069934 0.508254 -5.721041

H 0.896936 0.079601 -3.999342

H -0.001181 1.455778 -4.658928

H 3.398059 0.458293 -3.798978

H 2.152511 2.686750 -5.054311

C 1.909719 -3.336631 4.424465

H 1.165621 -4.119366 4.604978

H 1.436767 -2.355334 4.522872

H 2.722392 -3.434665 5.152033

H 2.306693 -3.441554 3.411619

C 2.222756 -6.326538 2.287117

C 1.090625 -5.758640 1.425892

H 1.338001 -5.836869 0.360120

H 0.150328 -6.299888 1.586298

H 0.916113 -4.702829 1.661911

H 2.417379 -7.378436 2.044592

H 3.151719 -5.766141 2.130318

H 1.976472 -6.266994 3.353449

C -7.650015 -0.069427 -4.326420

H -8.524323 -0.280424 -4.952060

H -7.380053 -0.996953 -3.803442

C -7.930837 1.044539 -3.361746

C -9.102129 1.752197 -3.219477

C -7.012944 1.585790 -2.389611

H -10.035304 1.655671 -3.758930

N -8.967591 2.701103 -2.218756

C -7.696620 2.622099 -1.688735

C -5.681662 1.288546 -2.038639

H -9.689756 3.342463 -1.923665

C -7.088652 3.352492 -0.657808

C -5.074332 2.005846 -1.012243

H -5.139328 0.499595 -2.555181

H -7.628103 4.133618 -0.127928

C -5.773759 3.026695 -0.329439

H -4.056571 1.776788 -0.718283

H -5.275481 3.552070 0.480573

H -6.811876 0.175445 -4.992869

------------------------------------

PC-W304

*G*(Water) =-4151.335051 Hartree

------------------------------------

C -1.256758 -5.346823 0.158440

C -0.930828 -3.926746 0.470128

C -1.352586 -3.098205 1.474639

N -0.083899 -3.152526 -0.315995

C 0.007253 -1.911973 0.181664

N -0.758448 -1.868542 1.269067

H -1.777622 -5.426901 -0.802435

H -1.911902 -5.751258 0.931580

H 0.401300 -3.469456 -1.147728

H -2.037023 -3.258421 2.289609

H 0.569482 -1.082356 -0.223430

P 7.093774 0.750958 0.342189

S 0.205561 1.692389 0.990924

O 7.621937 -0.693463 0.547743

O 6.618898 1.024566 -1.113387

O 6.084460 1.212214 1.404030

O 8.406756 1.760842 0.591076

O 0.733385 1.085866 -0.246869

O 0.904821 2.938269 1.416632

O -0.035432 0.751318 2.112679

C -3.599434 0.698933 -0.879911

N -5.634736 1.740655 -1.837071

O -4.102991 3.407995 0.014251

S -3.849876 -0.320260 2.903351

C -4.178938 1.791655 -1.799459

O -3.231860 0.063209 1.395826

C -3.741597 3.198438 -1.349413

O -3.929090 0.952844 3.658466

C -3.665100 2.457712 1.006987

O -2.821809 -1.271278 3.381369

C -3.969439 0.994353 0.579781

O -5.185879 -0.927820 2.636809

C -2.233034 2.675459 1.478217

O -1.305765 2.181134 0.481756

C -6.356653 1.281814 -2.892617

O -2.393745 3.478500 -1.634170

C -7.862021 1.265772 -2.705652

O -5.837513 0.895200 -3.948801

C -3.250289 -1.657013 -1.240341

O -4.136941 -0.541074 -1.306620

H -2.508293 0.694829 -0.976887

H -3.825557 1.616982 -2.816356

H -5.040516 0.797990 0.700042

H -4.321112 3.943248 -1.898414

H -6.135598 2.065020 -1.019656

H -4.282599 2.693160 1.876392

H -2.052110 3.739740 1.637170

H -2.085827 2.126232 2.407052

H -8.192681 1.707172 -1.761125

H -8.327930 1.803615 -3.537046

H -8.206476 0.227049 -2.747287

H -3.034573 -1.927563 -0.204373

H -0.350673 -5.961069 0.108934

H -3.765478 -2.487385 -1.726613

H -2.312744 -1.445708 -1.773680

H -1.809249 2.948071 -1.052348

C 9.572040 1.534673 -0.204471

H 10.015546 0.554481 0.015344

H 10.293971 2.317769 0.045449

H 9.338504 1.589736 -1.276051

H -0.896301 -1.017102 1.819677

C 12.225515 -5.414961 1.080644

H 12.403509 -4.760245 1.943030

H 12.970860 -5.171583 0.313136

C 10.794252 -5.239808 0.535359

H 10.647794 -5.933028 -0.304915

H 10.072016 -5.535074 1.308971

C 10.460602 -3.809086 0.060610

H 10.516023 -3.108021 0.903598

H 11.207521 -3.487193 -0.678852

C 9.066511 -3.757256 -0.574819

H 9.004625 -4.464868 -1.406037

H 8.286301 -4.008015 0.148458

N 8.713807 -2.398048 -1.127168

H 9.502314 -1.990632 -1.636805

H 8.363433 -1.674834 -0.392253

H 12.391801 -6.449838 1.400273

C 0.186180 5.781591 -3.222434

H -0.105277 4.994932 -3.929169

H 0.905231 6.440243 -3.725145

C 0.779728 5.182033 -1.939193

H 1.031222 5.985908 -1.236082

H 0.017863 4.564354 -1.448986

C 2.036759 4.330068 -2.171675

H 1.814466 3.501200 -2.859020

H 2.824403 4.944754 -2.624220

N 2.504341 3.812985 -0.880294

H 1.802618 3.741991 -0.144129

C 3.617815 3.090009 -0.653979

N 4.562356 2.925271 -1.593652

N 3.809722 2.561308 0.560949

H 4.382642 3.218339 -2.543181

H 5.338327 2.253506 -1.433033

H 3.059955 2.632684 1.239321

H 4.676023 2.045446 0.819687

H -0.706754 6.370371 -2.986783

C 4.475225 -0.471216 -3.111244

H 4.547998 0.562840 -3.483229

C 3.458768 -0.541379 -1.972344

O 5.769344 -0.943083 -2.719364

H 3.338051 -1.575285 -1.625704

H 3.788094 0.070669 -1.126072

H 2.479107 -0.167483 -2.296059

H 6.137567 -0.267837 -2.083824

H 4.153079 -1.099710 -3.950093

C -0.831348 6.100481 1.127476

H -1.499194 5.860986 0.294479

H -0.156007 5.257917 1.298100

H -0.250392 6.995001 0.881831

H -1.421933 6.285474 2.030360

C -2.352887 4.205439 4.574622

C -0.825007 4.075264 4.567077

H -0.344422 5.042415 4.760971

H -0.485323 3.374142 5.339797

H -0.453621 3.711717 3.602770

H -2.695967 4.899637 3.797230

H -2.838577 3.239485 4.395048

H -2.713266 4.586240 5.538400

H 7.941075 -2.468089 -1.798656

C -4.534907 -6.917998 0.406038

H -5.382205 -7.606022 0.283095

H -4.023751 -7.183338 1.338189

C -4.983907 -5.487144 0.422228

C -4.789963 -4.557276 1.420384

C -5.717391 -4.807142 -0.615762

H -4.284261 -4.669267 2.371016

N -5.362758 -3.348241 1.074542

C -5.937598 -3.469385 -0.170039

C -6.199968 -5.194464 -1.881495

H -5.302509 -2.489404 1.624766

C -6.619906 -2.526546 -0.950565

C -6.876426 -4.258767 -2.661365

H -6.042487 -6.207857 -2.244283

H -6.750969 -1.505379 -0.606652

C -7.083191 -2.937104 -2.198299

H -7.250984 -4.543027 -3.641847

H -7.607151 -2.227024 -2.833431

H -3.841132 -7.115847 -0.422497

------------------------------------

TS-W304

*G*(Water) =-4151.289147 Hartree

------------------------------------

C 0.632673 -4.332795 4.437767

C 0.230793 -3.096371 3.701438

C 0.507895 -2.658990 2.432521

N -0.544349 -2.094590 4.274620

C -0.724057 -1.097280 3.394625

N -0.089983 -1.421501 2.272517

H 1.231844 -4.087504 5.322325

H 1.231340 -4.971369 3.783337

H -0.923273 -2.105878 5.214319

H 1.085469 -3.118559 1.645667

H -1.294400 -0.195599 3.550296

P -5.524404 0.629789 -0.250599

S -2.291391 0.736968 0.447174

O -6.315687 1.839055 -0.790774

O -5.097103 -0.350904 -1.358243

O -4.363867 1.094142 0.689040

O -6.493868 -0.209916 0.797577

O -2.077020 1.197114 1.826032

O -2.210532 1.696254 -0.675017

O -2.476422 -0.696197 0.154325

C 2.960670 0.520465 1.040286

N 4.575575 2.112261 0.021034

O 2.079201 1.712790 -1.440830

S 2.958317 -2.921740 -0.696606

C 3.273536 1.978379 0.661958

O 2.342102 -1.624140 0.149168

C 2.186131 2.542252 -0.279087

O 2.680474 -2.654139 -2.128688

C 1.795544 0.305686 -1.246545

O 2.189826 -4.034556 -0.093614

C 2.792197 -0.308297 -0.235372

O 4.415551 -2.947060 -0.374900

C 0.318792 0.014117 -0.947965

O -0.096947 0.407345 0.339161

C 5.621967 2.794981 0.551398

O 0.972254 2.766763 0.372739

C 6.866324 2.873314 -0.307575

O 5.578964 3.331544 1.669812

C 3.647711 -0.895004 2.864151

O 4.026830 0.030521 1.845000

H 2.022808 0.507315 1.605345

H 3.302917 2.581700 1.569949

H 3.777633 -0.389951 -0.705089

H 2.530187 3.504255 -0.669970

H 4.678243 1.739255 -0.914260

H 2.003243 -0.139427 -2.223786

H -0.267475 0.542995 -1.713449

H 0.157139 -1.062656 -1.097532

H 6.770268 2.359114 -1.267557

H 7.104989 3.927567 -0.483732

H 7.698661 2.423425 0.240724

H 3.287204 -1.836274 2.438974

H -0.243403 -4.902246 4.768932

H 4.545340 -1.079787 3.460457

H 2.866760 -0.470196 3.511562

H 0.484436 1.883838 0.474799

C -7.630652 -0.897188 0.257151

H -8.322050 -0.197662 -0.229446

H -8.138757 -1.379204 1.096426

H -7.316324 -1.659704 -0.465324

H -0.045867 -0.765451 1.424777

C -2.846801 8.366700 -1.305270

H -2.881583 8.425799 -2.400481

H -1.815245 8.129992 -1.014910

C -3.822540 7.306206 -0.782165

H -3.799036 7.291204 0.316227

H -4.847857 7.580937 -1.066867

C -3.511846 5.901128 -1.317975

H -3.498475 5.926929 -2.416905

H -2.511784 5.587491 -0.991202

C -4.548264 4.883791 -0.852267

H -4.533962 4.757800 0.232199

H -5.556122 5.190783 -1.146612

N -4.326619 3.527195 -1.442307

H -3.485681 3.050807 -1.074046

H -5.152639 2.880240 -1.221230

H -4.228532 3.577564 -2.459497

H -3.089796 9.358254 -0.905889

C -5.989995 -7.971629 -1.809213

H -5.392884 -7.930370 -2.728360

H -6.978025 -7.548569 -2.027829

C -5.303385 -7.210159 -0.669749

H -5.907877 -7.275911 0.244339

H -4.328096 -7.663908 -0.449318

C -5.107912 -5.741664 -1.041783

H -4.486091 -5.672014 -1.944177

H -6.083308 -5.289129 -1.263877

N -4.471676 -4.993896 0.047642

H -4.177749 -5.508164 0.868194

C -4.256031 -3.669621 0.015326

N -4.538020 -2.965802 -1.080451

N -3.769660 -3.030081 1.100489

H -4.985418 -3.426920 -1.859551

H -4.625875 -1.923286 -1.069723

H -3.396499 -3.577493 1.865160

H -3.336681 -2.115064 0.953622

H -6.126322 -9.025534 -1.543780

C -3.472383 -1.195918 -4.121424

H -4.158035 -2.022861 -3.876770

C -2.153093 -1.371147 -3.372255

O -4.098454 0.058728 -3.839632

H -1.442066 -0.587307 -3.659841

H -2.309144 -1.308752 -2.290919

H -1.703623 -2.346374 -3.603526

H -4.447594 0.011176 -2.912583

H -3.305001 -1.223482 -5.204874

C -1.861573 4.795892 1.823752

H -2.334142 3.823526 1.664680

H -1.026893 4.908938 1.125941

H -2.587711 5.597798 1.662780

H -1.486443 4.850607 2.850960

C -6.874690 4.258616 2.318471

C -5.472922 3.816238 2.754847

H -5.062752 3.062708 2.073301

H -4.778308 4.664914 2.780973

H -5.493385 3.378697 3.761420

H -7.566914 3.408031 2.289309

H -7.292481 5.005957 3.004556

H -6.857952 4.702050 1.315272

C 8.906844 0.139063 -3.707866

H 9.070364 1.225343 -3.728981

H 8.394244 -0.139272 -4.635405

C 8.112363 -0.276487 -2.505201

C 6.883482 -0.898486 -2.485121

C 8.486414 -0.090674 -1.125459

H 6.264516 -1.221891 -3.312503

N 6.457126 -1.089778 -1.182608

C 7.425903 -0.612904 -0.327056

C 9.605643 0.477533 -0.488234

H 5.669854 -1.680724 -0.908455

C 7.453727 -0.566855 1.073003

C 9.642552 0.516727 0.904356

H 10.424402 0.887647 -1.075238

H 6.617289 -0.936930 1.658973

C 8.573918 0.002292 1.676602

H 10.499460 0.956342 1.409045

H 8.624261 0.059036 2.761180

H 9.899142 -0.331961 -3.718867

------------------------------------

RC-W304R(M7)

*G*(Water) =-4071.964005Hartree

------------------------------------

C -2.419645 3.417294 5.021625

C -1.580634 2.495992 4.195544

C -1.814173 1.848893 3.004602

N -0.303048 2.099584 4.561176

C 0.175448 1.248759 3.613454

N -0.720629 1.076304 2.653907

H -2.619879 2.997431 6.015325

H -3.379539 3.586046 4.524948

H 0.192991 2.392066 5.394044

H -2.682736 1.902826 2.367020

H 1.158769 0.797646 3.651881

P 5.518641 0.682157 0.165376

S 3.422056 -0.474329 1.934561

O 6.475572 -0.467992 -0.084029

O 4.517304 0.999675 -0.928229

O 4.759288 0.527370 1.624783

O 6.337010 2.024952 0.602240

O 3.199596 -0.184423 3.367900

O 3.879557 -1.846591 1.633133

O 2.348222 0.012025 1.036879

C -3.822991 -0.884345 0.231352

N -4.885801 -2.300042 -1.505719

O -2.012353 -2.321518 -1.517322

S -2.429730 2.438738 -1.122888

C -4.091726 -2.311543 -0.278820

O -2.502700 1.089227 -0.146605

C -2.775313 -3.058663 -0.563992

O -1.536607 2.081317 -2.251268

C -1.672315 -0.947947 -1.206206

O -1.846241 3.422666 -0.166054

C -2.925412 -0.142896 -0.767219

O -3.827477 2.731961 -1.518961

C -0.478468 -0.828715 -0.256802

O -0.789626 -1.147027 1.097416

C -6.216792 -2.501731 -1.547990

O -2.082100 -3.396173 0.604344

C -6.884691 -2.409709 -2.900246

O -6.880496 -2.749167 -0.520835

C -5.157866 0.851628 1.279524

O -5.095962 -0.263396 0.384284

H -3.315083 -0.941739 1.201597

H -4.658136 -2.860048 0.474892

H -3.536578 0.085385 -1.647018

H -3.004655 -3.998972 -1.071151

H -4.392968 -2.128714 -2.374222

H -1.339748 -0.542935 -2.165930

H 0.298595 -1.513877 -0.605563

H -0.085423 0.187578 -0.320179

H -6.177835 -2.335273 -3.730790

H -7.522164 -3.287282 -3.040958

H -7.531181 -1.525001 -2.905963

H -4.657793 1.729175 0.863098

H -1.934242 4.390989 5.163833

H -6.219939 1.068033 1.417756

H -4.710563 0.597088 2.250331

H -1.661143 -2.585049 0.991359

C 7.240169 2.594385 -0.367533

H 8.020928 1.875984 -0.640719

H 7.693940 3.464293 0.110303

H 6.695024 2.909984 -1.264082

H -0.784833 -0.304422 1.645618

C 12.893971 -3.951157 0.045849

H 12.849876 -4.465810 1.014029

H 13.559692 -3.085747 0.155595

C 11.495584 -3.514710 -0.409580

H 11.564181 -3.028217 -1.392425

H 10.857925 -4.399571 -0.542265

C 10.826799 -2.553509 0.584471

H 10.737454 -3.041283 1.565071

H 11.461430 -1.666916 0.721484

C 9.445652 -2.119252 0.103292

H 9.498269 -1.599074 -0.856572

H 8.767448 -2.968755 -0.003110

N 8.786078 -1.168108 1.062735

H 9.362589 -0.335384 1.214270

H 7.840997 -0.847993 0.686362

H 8.640407 -1.601628 1.978682

H 13.348126 -4.636050 -0.679526

C 0.289496 7.250687 -3.406576

H 0.453981 6.747852 -4.367599

H 1.196480 7.817319 -3.162163

C -0.044341 6.239650 -2.303083

H -0.223805 6.763523 -1.355160

H -0.969954 5.704675 -2.554355

C 1.081905 5.222902 -2.101859

H 1.251857 4.669076 -3.036870

H 2.010059 5.750174 -1.847292

N 0.741013 4.299164 -1.018580

H -0.244294 4.210255 -0.760743

C 1.536491 3.312229 -0.582064

N 2.792753 3.189455 -1.037488

N 1.089823 2.478857 0.369568

H 3.078043 3.720327 -1.848156

H 3.366231 2.356192 -0.836487

H 0.117473 2.517449 0.657539

H 1.626842 1.653970 0.648545

H -0.529943 7.965879 -3.536879

C 2.249235 0.099987 -3.320789

H 1.738722 0.754257 -2.597323

C 2.118321 -1.359108 -2.894735

O 3.613341 0.506544 -3.473773

H 2.636960 -2.015112 -3.603249

H 2.554004 -1.517186 -1.902917

H 1.063117 -1.656824 -2.857421

H 4.007334 0.593616 -2.576422

H 1.773598 0.255676 -4.296021

C 1.529407 -4.034441 -0.179795

H 1.526900 -3.862620 -1.260505

H 0.502467 -3.987580 0.194495

H 1.954975 -5.021827 0.029152

H 2.136721 -3.265780 0.307141

C 6.271866 -4.085563 -1.371825

C 5.085823 -3.258043 -1.879114

H 4.141453 -3.611887 -1.450092

H 5.201999 -2.204354 -1.604976

H 4.994997 -3.318139 -2.970857

H 6.370582 -4.005969 -0.281829

H 6.157645 -5.148636 -1.618475

H 7.214289 -3.741887 -1.816061

C -12.194893 -2.073535 -1.826329

H -12.627366 -1.069851 -1.732044

H -12.819654 -2.766441 -1.249387

C -10.743835 -2.098392 -1.336321

H -10.324232 -3.105205 -1.461972

H -10.130510 -1.417558 -1.940382

C -10.638375 -1.690037 0.134487

H -11.024026 -0.670839 0.263916

H -11.246149 -2.365281 0.752689

N -9.240124 -1.738033 0.569064

H -8.544599 -2.177617 -0.044848

C -8.781540 -1.288909 1.735544

N -9.616785 -0.738875 2.637717

N -7.467619 -1.378323 2.008748

H -10.614944 -0.879986 2.569069

H -9.272666 -0.467463 3.548817

H -6.810586 -1.551989 1.248615

H -7.096225 -0.947676 2.843903

H -12.252002 -2.366892 -2.880148

------------------------------------

PC-W304R(M7)

*G*(Water) =-4071.963353 Hartree

------------------------------------

C 4.138257 -3.522409 4.751951

C 2.842584 -3.098605 4.145247

C 1.974509 -3.747253 3.308133

N 2.279240 -1.845206 4.360412

C 1.124956 -1.732540 3.688114

N 0.930097 -2.882161 3.048004

H 4.955191 -2.865119 4.433057

H 4.374314 -4.542238 4.438793

H 2.676773 -1.112377 4.937008

H 2.009772 -4.728351 2.865272

H 0.476982 -0.868951 3.634381

P -3.581401 3.207954 -1.459874

S -1.837518 -0.778021 1.653500

O -4.098695 1.746149 -1.498120

O -2.613944 3.454371 -0.257270

O -3.067314 3.745151 -2.792225

O -4.924496 4.165132 -1.129543

O -1.010027 0.168866 2.441811

O -3.240634 -0.357777 1.438680

O -1.691792 -2.194330 2.074396

C 1.748866 -1.008702 -0.831141

N 2.624256 -0.560859 -3.105380

O -0.284808 -0.940429 -2.903964

S 0.630950 -4.694643 -0.177192

C 1.793908 -0.032290 -2.027235

O 0.711231 -3.024276 -0.012695

C 0.394614 0.272397 -2.595134

O -0.400926 -4.947768 -1.213165

C -0.430389 -1.917403 -1.859668

O 0.239181 -5.072802 1.201903

C 0.936382 -2.256366 -1.207146

O 1.999612 -5.113448 -0.572035

C -1.541006 -1.619777 -0.852752

O -1.097965 -0.671859 0.151895

C 3.906938 -0.191317 -3.301886

O -0.344386 1.156713 -1.805061

C 4.668322 -0.896640 -4.399639

O 4.447345 0.692896 -2.609691

C 3.367350 -1.969806 0.715878

O 3.111195 -1.295504 -0.523529

H 1.273807 -0.516380 0.025343

H 2.247574 0.904744 -1.702034

H 1.533504 -2.844535 -1.913203

H 0.509729 0.767735 -3.561197

H 2.225496 -1.276432 -3.701310

H -0.771392 -2.809568 -2.388986

H -2.411584 -1.211904 -1.371243

H -1.805285 -2.555736 -0.360171

H 4.045538 -1.559024 -5.006675

H 5.138657 -0.147283 -5.042908

H 5.467514 -1.486924 -3.937738

H 3.090496 -3.023578 0.659925

H 4.089593 -3.498388 5.846452

H 4.441567 -1.876574 0.892149

H 2.822013 -1.489060 1.536648

H -0.488883 0.797964 -0.906864

C -5.765493 3.793189 -0.038178

H -6.361551 2.905256 -0.287928

H -6.447919 4.627890 0.149835

H -5.186984 3.597975 0.872905

H 0.137173 -3.050713 2.423475

C -10.297311 -1.909574 -0.577911

H -10.674615 -1.359426 0.293339

H -9.988100 -2.906171 -0.238141

C -9.125387 -1.161775 -1.236555

H -8.794290 -1.714240 -2.126951

H -9.476300 -0.182676 -1.591815

C -7.919736 -0.950505 -0.301647

H -8.244574 -0.416070 0.602178

H -7.525351 -1.922840 0.022934

C -6.816778 -0.148427 -0.995483

H -6.432692 -0.670986 -1.875041

H -7.191780 0.825699 -1.322044

N -5.631597 0.132501 -0.120383

H -5.093733 -0.706564 0.116271

H -5.910573 0.550837 0.770444

H -11.125752 -2.034779 -1.284450

C 4.612802 5.917323 2.253897

H 4.045676 6.807717 2.551044

H 5.050799 6.107430 1.266428

C 3.713696 4.670664 2.231199

H 4.312184 3.789303 1.966250

H 3.301056 4.492551 3.232905

C 2.556654 4.807508 1.235635

H 1.959599 5.693324 1.484944

H 2.951528 4.937693 0.219164

N 1.683947 3.625186 1.283471

H 2.013405 2.814691 1.792253

C 0.460597 3.546998 0.734733

N -0.023893 4.545471 -0.017981

N -0.319937 2.475958 0.922148

H 0.567622 5.281090 -0.374471

H -0.980827 4.432918 -0.376853

H -0.168442 1.797999 1.662615

H -1.276599 2.548417 0.542714

H 5.432990 5.783948 2.967238

C -2.483738 4.316334 2.969098

H -1.416800 4.121282 2.783068

C -3.211568 3.000297 3.238443

O -3.051480 5.050229 1.883482

H -4.274855 3.185255 3.435061

H -3.128670 2.322916 2.382699

H -2.779309 2.494347 4.111471

H -2.962207 4.493387 1.065316

H -2.546968 4.970518 3.847145

C -4.050546 -1.095875 -3.606030

H -3.026177 -1.269927 -3.950141

H -4.075907 -0.194910 -2.985203

H -4.711817 -0.968885 -4.469661

H -4.384325 -1.959290 -3.021206

C -3.839299 -4.819035 0.144054

C -4.570215 -3.471840 0.106870

H -4.568870 -3.053035 -0.907084

H -5.616199 -3.576095 0.421214

H -4.087010 -2.747744 0.771638

H -2.798213 -4.720514 -0.183612

H -3.830953 -5.233842 1.159774

H -4.326124 -5.552555 -0.510942

H -4.952595 0.826710 -0.612402

C 9.794604 0.512915 -3.033871

H 10.393481 -0.112376 -2.360289

H 10.183580 1.537146 -2.979439

C 8.310200 0.464351 -2.659265

H 7.727213 1.074955 -3.361054

H 7.933712 -0.563476 -2.740091

C 8.071463 0.969733 -1.234722

H 8.628348 0.348330 -0.522320

H 8.432429 2.003180 -1.140170

N 6.643042 0.904645 -0.914136

H 5.975239 0.771730 -1.683594

C 6.119949 1.078682 0.295800

N 6.903458 1.370764 1.350744

N 4.787475 0.957716 0.467269

H 7.839939 1.725566 1.215248

H 6.488807 1.557890 2.253721

H 4.248824 0.421668 -0.214534

H 4.420182 0.943689 1.409397

H 9.947524 0.148963 -4.055643

------------------------------------

TS-W304R(M7)

*G*(Water) =-4071.922829 Hartree

------------------------------------

C 0.869538 -4.758361 3.607922

C 0.536235 -3.454342 2.958566

C 0.613169 -3.037085 1.655378

N 0.074017 -2.353404 3.670969

C -0.116533 -1.318082 2.838630

N 0.208698 -1.714354 1.612042

H 1.675654 -4.638980 4.341131

H 1.198149 -5.473045 2.849173

H -0.098008 -2.326707 4.669264

H 0.928433 -3.565544 0.768969

H -0.484713 -0.338563 3.099169

P -5.149763 1.444283 0.466401

S -1.890679 0.890500 0.334911

O -5.793077 2.805703 0.130099

O -5.214441 0.442150 -0.701416

O -3.724094 1.634837 1.078618

O -5.974882 0.768527 1.733126

O -1.254900 1.273061 1.604081

O -1.910338 1.838976 -0.799737

O -2.418824 -0.472619 0.138788

C 3.186147 -0.332390 -0.349740

N 4.754428 0.932073 -1.801239

O 1.981974 1.087651 -2.563583

S 2.040184 -3.648520 -2.022435

C 3.671470 1.047294 -0.825561

O 1.975706 -2.293506 -1.037019

C 2.517349 1.841291 -1.472068

O 1.267330 -3.301002 -3.242259

C 1.494773 -0.250569 -2.289487

O 1.390798 -4.645339 -1.135689

C 2.565719 -1.079154 -1.535962

O 3.484156 -3.888321 -2.277342

C 0.103963 -0.270501 -1.643100

O 0.092342 0.149808 -0.297872

C 6.060222 1.069264 -1.506161

O 1.568036 2.257160 -0.540004

C 7.047247 0.890243 -2.636453

O 6.452396 1.327584 -0.349534

C 4.013981 -2.012662 1.178316

O 4.312713 -1.017248 0.193604

H 2.428925 -0.184124 0.427576

H 4.055249 1.606700 0.028660

H 3.379802 -1.337416 -2.222048

H 2.930291 2.738094 -1.943532

H 4.490866 0.752972 -2.763080

H 1.376476 -0.691110 -3.283129

H -0.536035 0.386308 -2.250061

H -0.288621 -1.291953 -1.736020

H 6.574845 0.808332 -3.618866

H 7.742945 1.734322 -2.635720

H 7.628215 -0.019143 -2.446475

H 3.492945 -2.867456 0.741490

H -0.000001 -5.179302 4.125738

H 4.975032 -2.334779 1.586350

H 3.396893 -1.591357 1.984711

H 0.956884 1.476220 -0.319385

C -7.334280 0.369062 1.513900

H -7.959503 1.228544 1.241205

H -7.696269 -0.055051 2.454107

H -7.394383 -0.390154 0.724999

H 0.179771 -1.056879 0.768301

C -1.326019 8.504479 -1.455768

H -1.751044 8.683304 -2.451534

H -0.340549 8.040441 -1.590291

C -2.246380 7.607957 -0.618951

H -1.820594 7.472740 0.384481

H -3.215925 8.107026 -0.481938

C -2.475885 6.231486 -1.259968

H -2.855618 6.364915 -2.283016

H -1.522894 5.691689 -1.339992

C -3.474237 5.403085 -0.457545

H -3.117361 5.207701 0.555414

H -4.436876 5.917338 -0.385673

N -3.744715 4.068597 -1.078564

H -2.960650 3.403588 -0.963933

H -4.595012 3.602875 -0.621757

H -3.935065 4.154928 -2.080146

H -1.176745 9.477221 -0.972816

C -7.680154 -6.862122 -0.704981

H -7.321126 -6.924804 -1.739507

H -8.591521 -6.251866 -0.699248

C -6.610315 -6.265115 0.215805

H -6.981405 -6.226670 1.248303

H -5.716199 -6.902403 0.214117

C -6.230590 -4.856689 -0.237123

H -5.846163 -4.892992 -1.265187

H -7.123892 -4.218506 -0.228581

N -5.215251 -4.269385 0.642811

H -4.828634 -4.851122 1.375093

C -4.758067 -3.013477 0.520830

N -5.167255 -2.240005 -0.484049

N -3.893372 -2.511753 1.428387

H -5.881742 -2.582550 -1.110095

H -5.032342 -1.202427 -0.486298

H -3.451295 -3.142872 2.084225

H -3.340681 -1.696132 1.153036

H -7.949976 -7.872988 -0.380789

C -4.517885 -0.675766 -3.758266

H -5.287920 -1.339654 -3.333999

C -3.127646 -1.160205 -3.350705

O -4.763869 0.676315 -3.361754

H -2.353056 -0.540027 -3.817672

H -2.999195 -1.104180 -2.265282

H -2.973266 -2.200967 -3.666144

H -4.882996 0.680399 -2.377310

H -4.629458 -0.704845 -4.848802

C -0.468832 4.820728 1.651012

H -0.903950 3.837962 1.451392

H 0.443218 4.699092 2.244395

H -0.220798 5.314274 0.706541

H -1.183925 5.433775 2.207403

C -5.085357 3.982121 4.039779

C -3.772825 4.366748 3.347282

H -3.565927 3.695881 2.506959

H -3.813827 5.394702 2.965078

H -2.924444 4.308261 4.040882

H -5.045728 2.952915 4.417791

H -5.301717 4.641476 4.889763

H -5.931692 4.044569 3.344812

C 11.931759 0.693237 -0.207843

H 12.332102 -0.300127 0.029354

H 12.376388 1.412583 0.490809

C 10.402637 0.708409 -0.119068

H 10.022557 1.703689 -0.384604

H 9.976757 -0.000700 -0.840435

C 9.915171 0.342924 1.284457

H 10.265591 -0.663123 1.548114

H 10.327663 1.050156 2.017326

N 8.451418 0.373934 1.329950

H 7.937531 0.773019 0.535570

C 7.706780 -0.026444 2.358648

N 8.283608 -0.516169 3.474160

N 6.367426 0.043536 2.274581

H 9.265240 -0.356870 3.653843

H 7.719030 -0.723039 4.287082

H 5.924435 0.214792 1.372240

H 5.790223 -0.338733 3.010218

H 12.262827 0.956861 -1.218176
